# *Oryza* genera-specific novel Histone H4 variant predisposes H4 Lysine5 Acetylation marks to modulate salt stress responses

**DOI:** 10.1038/s41477-025-01974-2

**Published:** 2025-04-08

**Authors:** Vivek Hari-Sundar Gandhivel, Paula Sotelo-Parrilla, Steffi Raju, Shaileshanand Jha, Anjitha Gireesh, Chitthavalli Y. Harshith, Fabian Gut, Kutti R. Vinothkumar, Frédéric Berger, A. Arockia Jeyaprakash, P.V. Shivaprasad

**Affiliations:** 1 National Centre for Biological Sciences, TIFR, GKVK Campus, Bangalore 560065, India; 2 SASTRA University, Thirumalaisamudram, Thanjavur 613 401, India; 3 Centre for Cell Biology, School of Biological Sciences, University of Edinburgh, Edinburgh, EH9 BF UK; 4 Gene Center and Department of Biochemistry, Ludwig-Maximilian-Universität, München, Feodor-Lynen Straße 25, 81377 Munich, Germany; 5 Gregor Mendel Institute of Molecular Plant Biology, Austrian Academy of Sciences, Dr. Bohr-Gasse 3 1030, Vienna, Austria

**Keywords:** epigenetics, histone variants, nucleosomes, histone modifications, salt stress, rice, H4 variant

## Abstract

Paralogous variants of canonical histones guide accessibility to DNA and function as additional layers of genome regulation. Across eukaryotes, mechanism of action and functional significance of several variants of core histones are well-known except that of histone H4. Here we show that, a novel variant of H4 (H4.V) expressing tissue-specifically among *Oryza* members, mediated specific epigenetic changes contributing to salt tolerance. H4.V was incorporated to specific heterochromatic sites where it blocked deposition of active histone marks. Stress dependent re-distribution of H4.V enabled incorporation of active H4 Lysine5 Acetylation (H4K5Ac) marks. Mis-expression of H4.V led to defects in reproductive development and in mounting salt stress responses. H4.V formed homotypic nucleosomes and mediated these alterations by conferring distinct molecular properties to the nucleosomes, as seen with cryo-EM structures and biochemical assays. These results not only uncovered the presence of a H4 variant among plants, but also of a novel chromatin regulation that might have contributed to the adaptation of semi-aquatic *Oryza* members.

## Introduction

Histones are modular proteins conserved across eukaryotes aiding in packaging the nuclear DNA as chromatin ^1^. Excluding archaea and few instances of eubacteria ^2–4^, roughly 147 bp DNA is wrapped around an octamer of two units of histones H2A, H2B, H3 and H4, forming nucleosomes in eukaryotes ^5^. In addition to DNA modifications, nucleosomal histones undergo several post-translational modifications (PTMs), predominantly at the tails, some of which reflect transcriptional activity and chromatin architecture ^6,7^. Several paralogs of H2A, H2B and H3 histones, named histone variants, have been identified across eukaryotes that are expressed and incorporated into the chromatin independent of cell cycle ^8^. H3 and H2A.Z variants are distinguished from their counterparts by specific histone chaperone complexes that assist their incorporation in chromatin and often perform essential roles ^9,11^.

By virtue of their specific residue modifications and variations, it has been shown that H2A variants confer unique properties to the nucleosomes thus regulating chromatin ^10,12^. In addition to altering the chromatin properties, specific histone variants facilitate essential cellular processes such as chromosome segregation during cell division (CENH3), DNA damage responses (H2A.X), and gametic inheritance of epigenetic states (H3.10) ^13–18^. H2A.W was identified as a plant specific histone variant that functions in efficient silencing of transposons at the heterochromatin, acting as a determinant of several repressive chromatin states ^17,19,20^.

Multiple functionally relevant H2A, H2B and H3 variants have been described among plants and animals ^21^. However, only a very few variants in H4 has been described and all were in parasites ^22^ with the exception of H4 variant (H4G) specific to the *Hominidae* family. H4G has been characterised as a regulator of rDNA expression in specific cancer cells ^23,24^. In contrast, histone H4 sequences are highly conserved and variants of histone H4 have not been functionally characterised in plants.

Evolution of monophyletic clade-specific histone variants having very specific functions have been documented in several cases ^18,25,26^. Most of the monophyletic histone variants repurpose the canonical histone chaperones for incorporation ^23^. Among such histone variants, functional outcome is dependent on the histone variant but usually not on the chaperone.

Plant responses are unique to different types of stresses and are distinct in different species ^27,29^. Tuned responses to stress is important for tolerance and plants have evolved several layers of regulatory modules to counteract aberrant activation of stress responsive genes ^28,30^. Chromatin level changes upon stress was identified as a powerful module of stress responses as several genes were regulated rapidly ^31,32^. Plants have a specific ability to adjust their capacity to respond to stress based on previous experiences of stress, therefore showing forms of epigenetic stress memory ^33^. Histone deacetylases and other modifiers were implicated in modulating global stress responses via genome-wide changes in reprogramming of histone modifications ^34,35^. These modifiers regulated histone marks such as H4K5Ac or H3K4me, thereby regulating stress responsive TFs ^36–38^. Histone variants were also found to initiate signaling during abiotic stress responses ^40^. Importance of H2A.Z has been well-documented during heat and other stresses, where H2A.Z containing nucleosomes poise transcription for stress tolerance and responsive genes ^39,41–44^. However, roles of other histone variants or mechanism by which the histone variants modulate the chromatin to facilitate stress responses are not understood. Most of the above studies were carried out in a seasonal model plant *Arabidopsis* having small homogenous compartmentalized genome, while the diversity, complexity, properties and regulation of chromatin, among plants with larger genomes such as crops, seem to be heterogenous and species-specific.

In this work, we identified and functionally characterized a novel *Oryza* genera specific histone H4 variant that exhibited sequence conservation within the genera. This is the first report to functionally characterize a variant of H4 in plants. Using molecular, genetic, and genomic approaches, we established the functional role of the H4.V in rice. Analyses of cryo-EM structures and biochemical analyses of the H4.V and H4 containing rice nucleosomes revealed unique properties of the H4.V that can aid in chromatin regulation. Genetic studies with mis-expression lines including mutants of the gene indicated its surprising and unique roles in synergistic modulation of H4K5Ac chromatin marks under salt stress conditions. These results indicate that the H4.V might have contributed significantly to the adaptation of semi-aquatic *Oryza* members to changing environments.

## Results

### Novel *Oryza* genera specific histone H4 variant predominantly occupied intergenic loci

**Extended Data Fig. 1:**
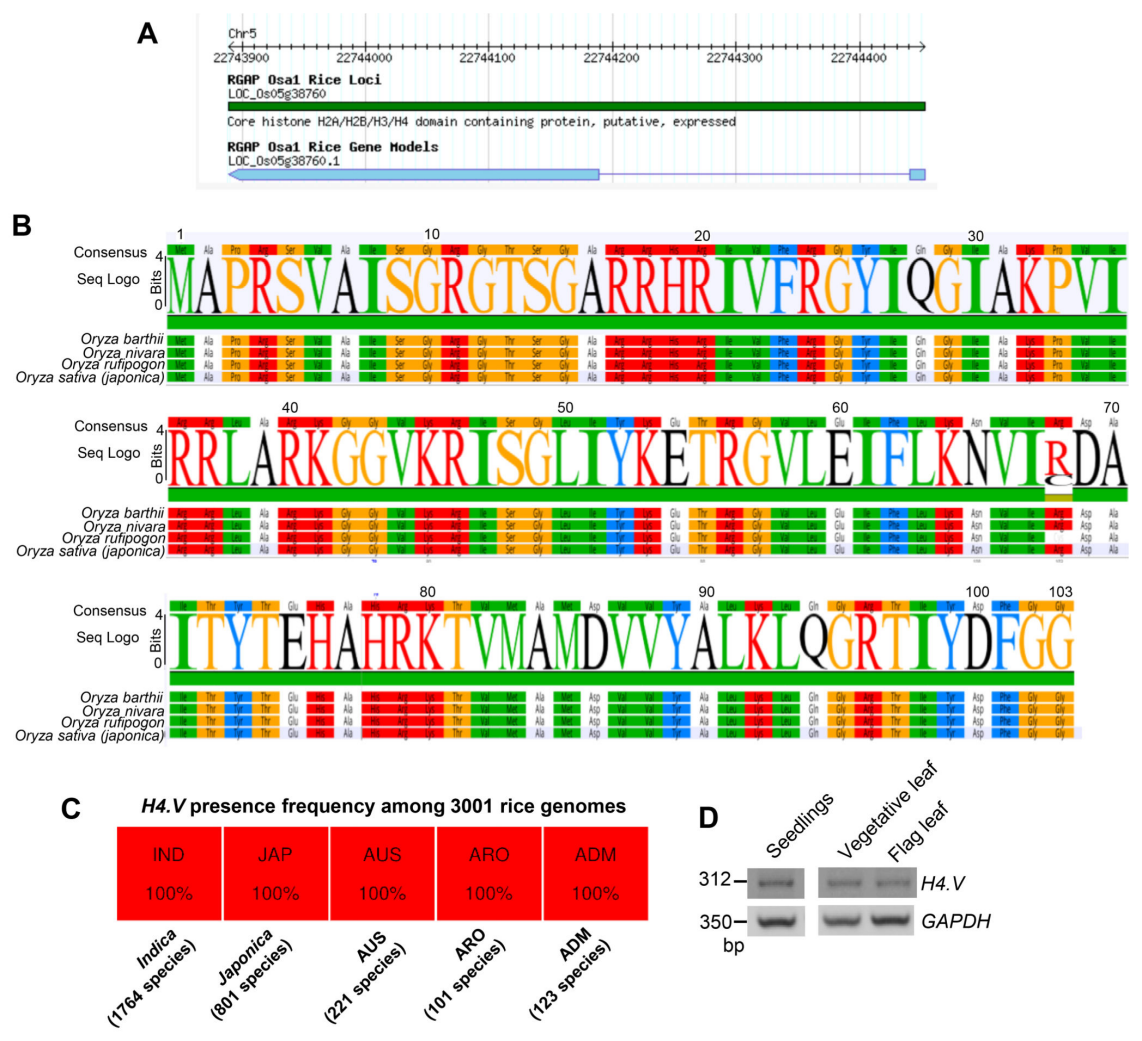
H4.V is conserved among *Oryza* genera members. (A) Gene model of the H4.V from RAPDB (https://rapdb.dna.affrc.go.jp/). (B) Seq-logo showing conservation of the H4.V among rice species. (C) H4.V presence frequency among the 3001-rice genome project from rice pan-genome browser (https://cgm.sjtu.edu.cn/3kricedb/index.php). (D) RT-PCR amplification of the H4.V transcript across three rice tissues. *GAPDH* served as control.

**Fig. 1:**
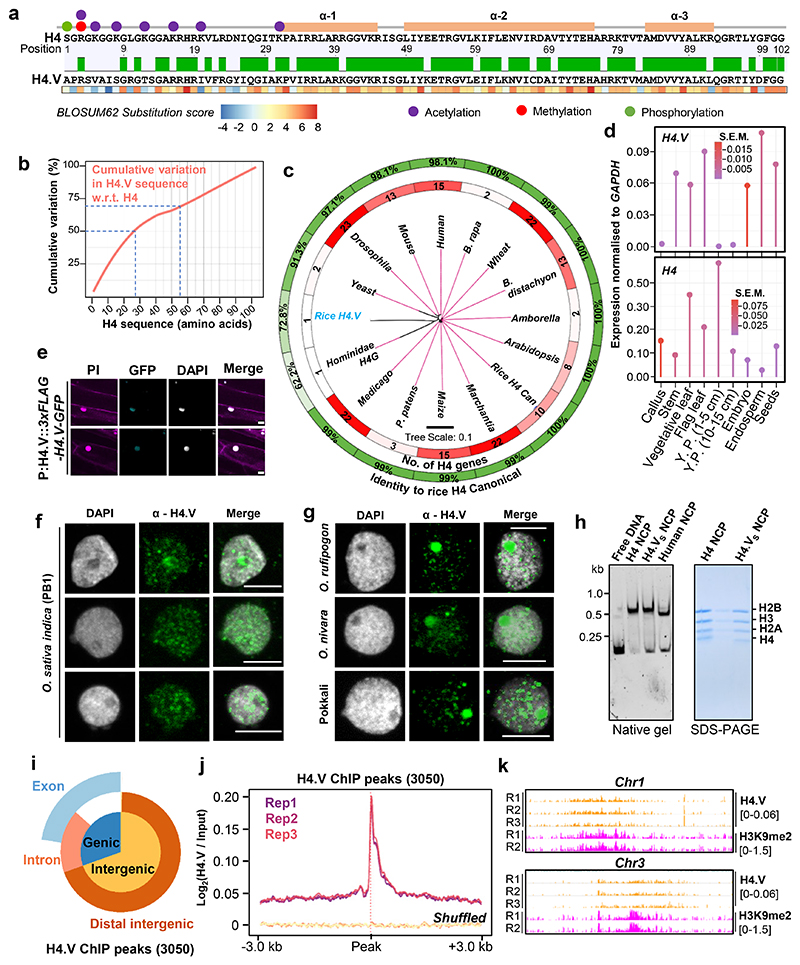
Rice specific histone H4.V is different from H4. (a) Pairwise sequence alignment of H4 and H4.V. Line diagram shows histone-fold domains (orange boxes) and H4 residues that attract PTMs (colour coded). Heatmap with BLOSUM62 scores for the substitutions observed. (b) Cumulative variations plot showing positional distribution of variations across protein length from N-terminal end (x-axis). Dotted lines represent the intercepts for 50% and 70% variation. (c) Phylogenetic tree with protein sequence variations of H4 across organisms. Inner track-number of H4 encoding genes; outer track-% identity w.r.t. rice H4. Tree distance is in black. (d) Lollipop plots showing RT-qPCR estimation of H4 and H4.V expression in different rice tissues. Circle diameters represent standard error of the mean (S.E.M.). Y.P.: young panicle. (e) Fluorescent micrographs of seedling roots expressing 3xFLAG-H4.V-GFP driven by H4.V promoter (P:H4.V). (f-g) IFL images of PB1 (f), *Oryza* species and pokkali (g) nuclei stained using α-H4.V antibody. (h) Gel-mobility shifts of *in vitro* reconstituted H4.V_S_ and H4 containing NCPs on a native gel (6% acrylamide) and Coomassie brilliant blue (CBB) stained SDS-PAGE gel (15% acrylamide). Human NCPs were used as control. (i) Vennpie chart showing proportion of genic and intergenic regions (beyond +/- 3 kb of genes) overlapped by H4.V peaks. (j) Metagene plots showing enrichment of H4.V ChIP-seq signal in 3 biological replicates of rice seedlings over H4.V peaks. Enrichment at the shuffled set of loci served as control. (k) Chromosome-wide genome browser screenshots showing ChIP enrichment (square brackets) of H4.V and H3K9me2 marks in seedlings. (e-j) DAPI stained DNA; propidium iodide (PI) stained DNA and cell walls. Scale: 5 μm.

The Rice genome comprises ten histone H4 genes of which nine encode identical H4 protein (named H4 throughout) ^45^. One exception is the gene locus LOC_Os05g38760 (MSU gene model) (Extended Data Fig. 1a). This gene locus encoded a previously uncharacterized histone H4-like protein that we named as H4.V. Sequence alignment indicated that H4.V resembled H4 in length (103 amino acids) with similar histone fold domains albeit with minor modifications (Fig. 1a). Majority of the variations in sequence were confined to the N-terminal tail, with the first 27 and 55 residues encompassing 50% and 70% of all the variations respectively (Fig. 1b). Particularly, the first 20 residues had the most disfavoured amino acid substitutions on a BLOSUM62 substitution scale (Fig. 1a). H4.V showed 72.8% sequence identity to H4 that was largely conserved across plants and metazoans (Fig. 1c and Supplementary Table 1). H4.V was almost identical among ancient rice species - *O. nivara, O. barthii, O. rufipogon*, and modern *japonica* and *indica* subspecies of *O. sativa* (Extended Data Fig. 1b). *H4.V* was present in all the 3001 sequenced rice (Extended Data Fig. 1c) but similar sequences were absent in other genera indicating that H4.V is likely *Oryza* specific.

**Extended Data Fig. 2:**
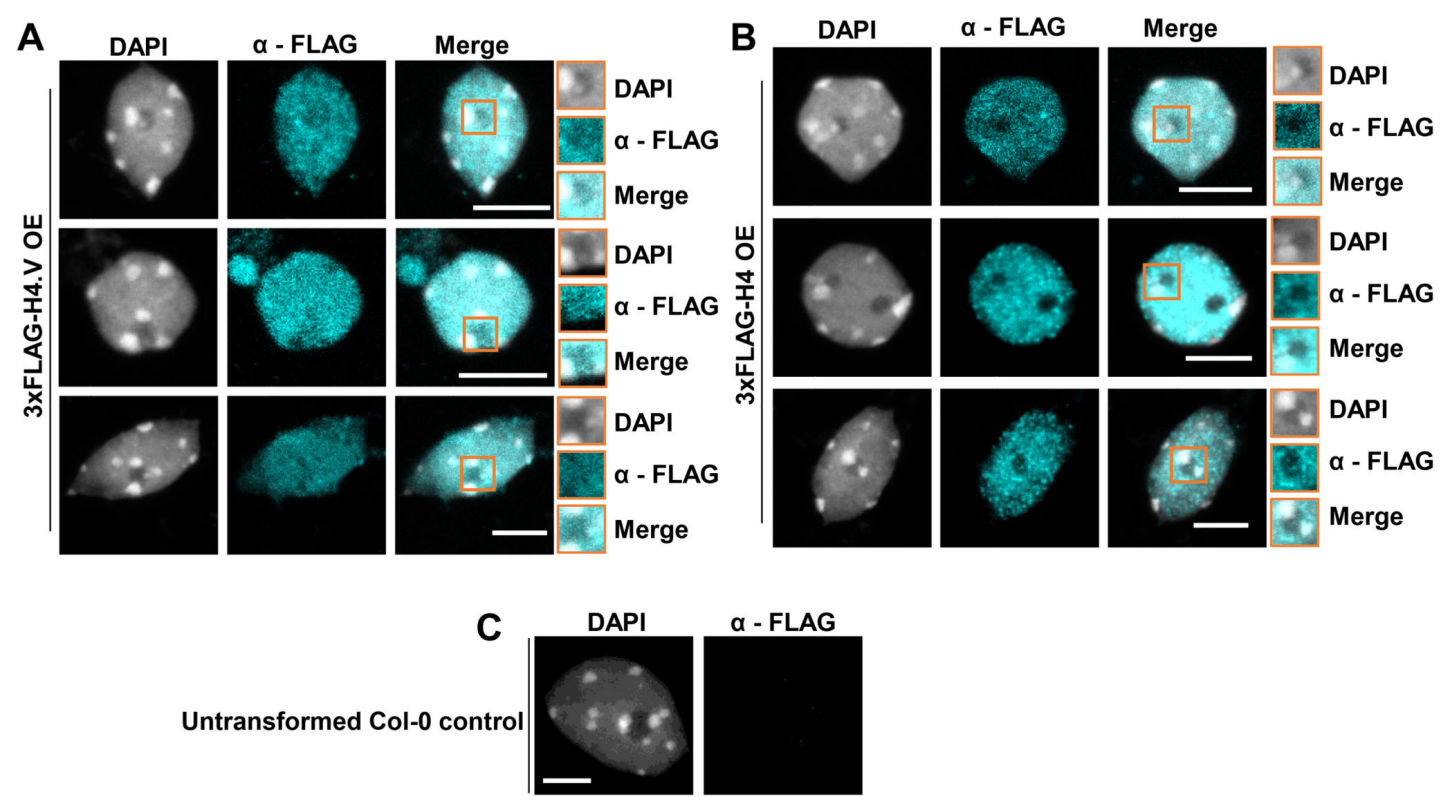
H4.V can be incorporated into the *Arabidopsis* chromatin. (A-C) Immunostaining of nuclei from *Arabidopsis* Col-0 plants heterologously over-expressing (CaMV 35S promoter) H4.V (A) or H4.V_S_ (B). Nucleolar regions are shown as insets. (C) Untransformed Col-0 control shows specificity of α − FLAG antibody in immunofluorescence imaging. Scale: 5 μm.

**Extended Data Fig. 3:**
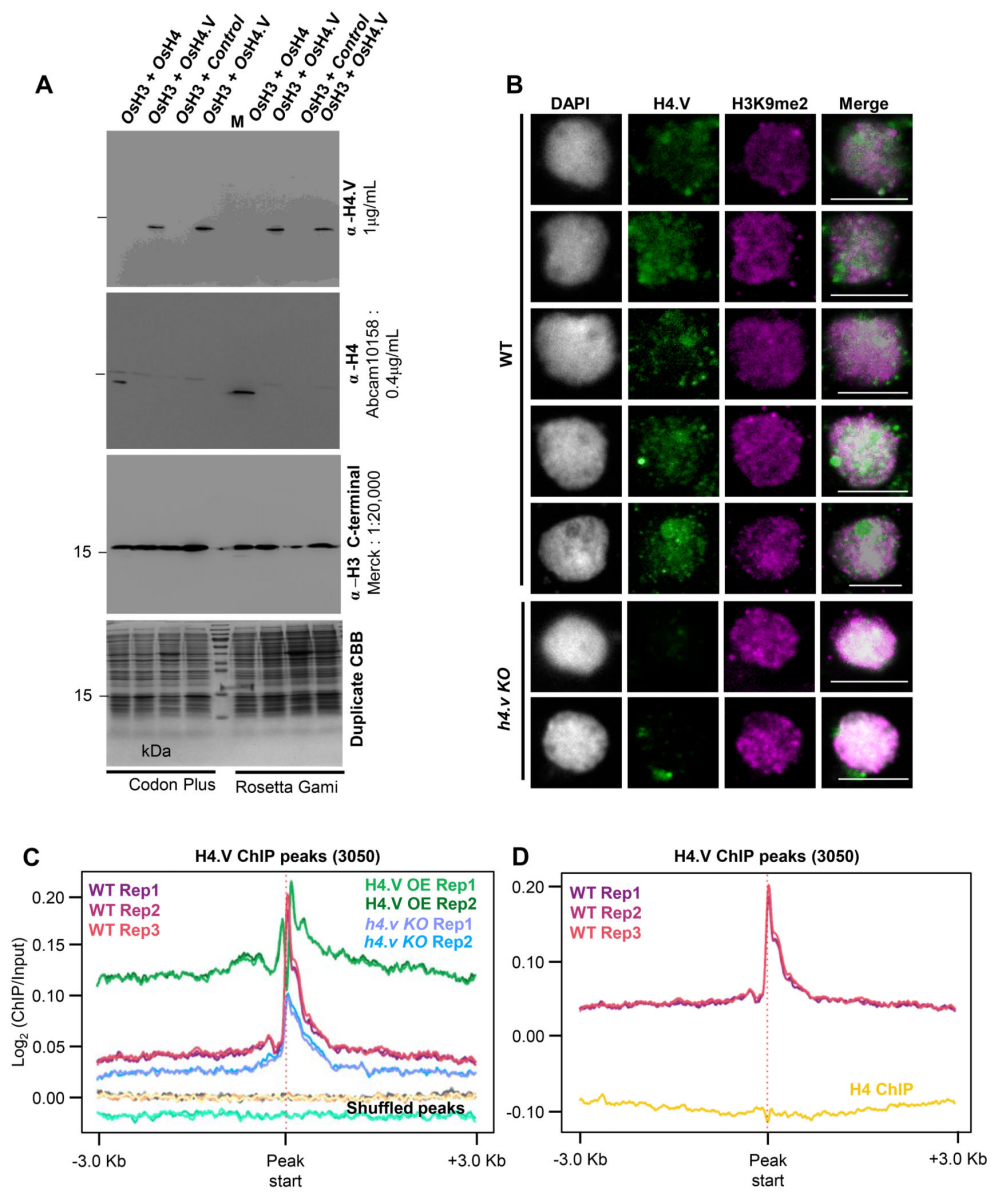
H4.V antibody is specific and does not cross react with H4. (A) Immunoblots showing reactivity of α-H4.V and α-H4 specifically to their epitopes. Total bacterial lysates from two different *E. coli* strains are shown. Blots were stripped and re-hybridized. CBB stained duplicate gel served as loading control. Both H3 and H4/H4.V were co-expressed in the same plasmid (Supplemental Table S5). M: size marker. (B) IFL images of nuclei from WT and *h4.v KO* plants stained using α-H4.V and α-H3K9me2. Scale: 5 μm. (C) ChIP enrichment profiles of H4.V at H4.V peaks from WT, OE-H4.V and *h4.v KO*. Enrichment profiles at shuffled H4.V peaks served as control. (D) ChIP enrichment profiles at H4.V peaks from H4.V ChIP-seq and H4 ChIP-seq.

*H4.V* was cloned from cDNA and its sequence matched the coding sequence in the reference genome (Extended Data Fig. 1d). Expression profiling revealed that *H4.V* expressed in a tissue-specific manner and overall transcript levels of *H4.V* were lower than *H4* (Fig. 1d). *H4.V* was expressed in most of the tissues that were tested and was not detected in young panicles and scutellar calli. GFP tagged H4.V fusion protein, driven by its native promoter, was localised to the nucleus in rice root cells (Fig. 1e). Expressing 3xFLAG-H4.V in *Arabidopsis* (that naturally lacks H4.V) indicated that H4.V is incorporated in the nucleus, suggesting that H4.V incorporation is dependent on conserved chaperones (Extended Data Fig. 2). In order to demonstrate that H4.V is a true variant capable of incorporation into the chromatin and to study its occupancy, we raised specific polyclonal antibodies (Methods) that did not cross react with H4 (Extended Data Fig. 3).

Immunofluorescence (IFL) studies with this antibody revealed that H4.V is expressed and deposited in the chromatin of *indica* rice, its wild relatives including *O. nivara, O. rufipogon* and the locally cultivated landrace pokkali, suggesting the conservation of the variant among *Oryza* genera members (Fig. 1f,g). We observed occupancy of H4.V at the perinucleolar rDNA rich regions as observed in the case of human H4G variant earlier^23^. When the H4.V was over-expressed heterologously in *Arabidopsis*, we observed similar perinucleolar occupancy in IFL microscopy, unlike H4 (Extended Data Fig. 2). To test if H4.V is incorporated into the nucleosomes, we purified rice histones H2A, H2B, H3, H4.V and H4 recombinantly in *E. coli* and performed gradual native buffer dialysis to enable refolding into octamers (Methods). The histone octamers were insoluble when folded *in vitro* with H4.V but were soluble when the N-terminal end (53 amino acids, accounting for most of the variation) of H4.V was fused with C-terminal 50 residues of H4 (named H4.V_S_ throughout). With recombinant H4.V_S_ or H4 and H2A, H2B and H3 from rice and Widom 601 nucleosome positioning DNA sequence (NPS) ^47^, we successfully reconstituted nucleosome core particles (NCPs) (Methods, Supplementary Table 5). NCPs of H4.V_S_ and H4 exhibited similar mobility shift profiles in gel shift assays, suggesting that H4.V can be incorporated into the nucleosomes *in vitro* and matches the profile of characterized human nucleosomes (Fig. 1h).

**Extended Data Fig. 4:**
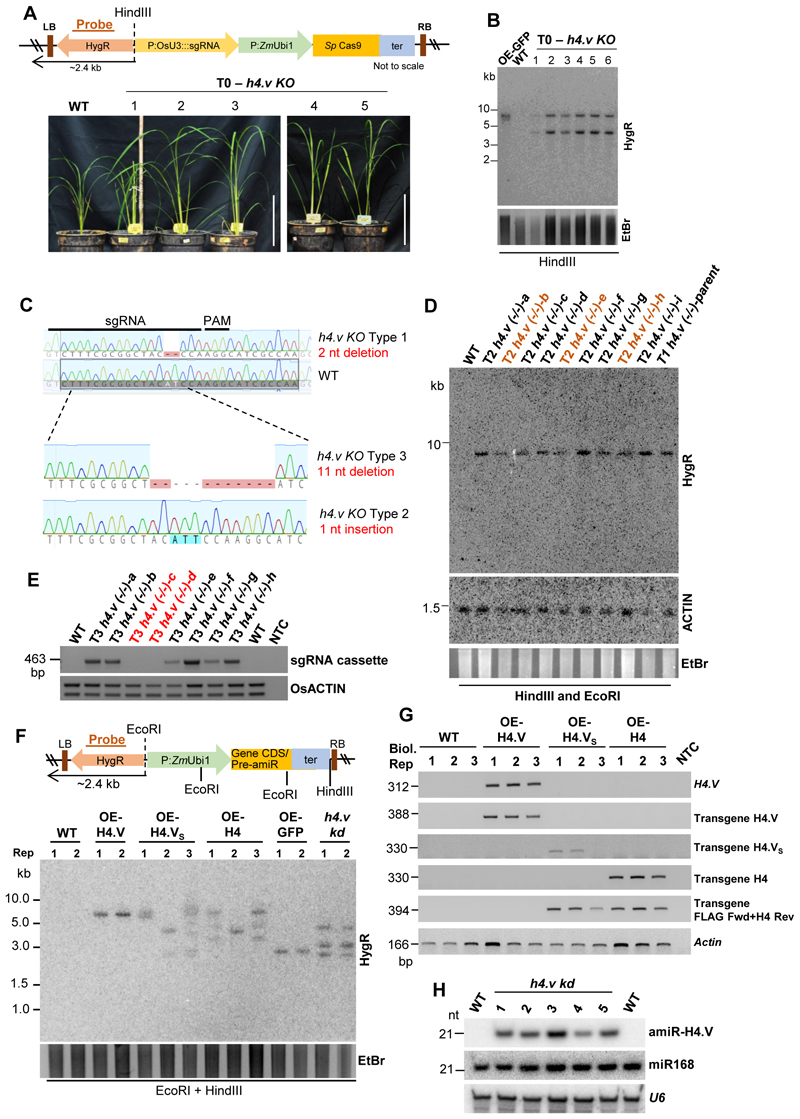
Generation of *h4.v KO* and H4.V perturbed lines in rice. (A) T-DNA map of the Cas9 construct used for generating the *h4.v KO*. T0 *h4.v KO* images of 5-weeks old plants are shown. Scale: 10 inches. (B) Southern blots showing the junction fragment profiles of T0 *h4.v KO* lines. OE-GFP served as positive control. (C) Mutation profiles of T1 *h4.v KO* plants compared to WT. Three types of mutations were obtained and the type 1 mutation was taken for the analysis. (D) Junction fragment Southern analysis of the homozygous (-/-) T1 *h4.v KO* parent (that segregated as single copy transgene) and its T2 progeny. Plants marked in brown are putative parents that are heterozygous for the transgene. (E) Genomic DNA PCR showing the T3 segregants of the T2 parents (marked brown in (D)). Actin was used a loading control and sgRNA region as transgene marker. (A, B and D) Probe (HygR) used for T-DNA junction fragment Southern blots is marked (brown). EtBr-stained gel served as loading control. (F) T-DNA map and the junction fragment (arrow) Southern analyses of transgenic plants overexpressing H4.V, H4.V_S_ and H4 or GFP (3x-FLAG tagged at the N-terminal) or the precursor of amiR. HygR was used as probe (brown bar). EtBr-stained gel is the loading control. (G) RT-PCR (semi-quantitative) gels showing the OE of the transgenes in leaves using specific primers. NTC-no template control. Actin was used as loading control. (H) sRNA northern blots showing the accumulation of the amiR targeting H4.V in *h4.v kd* plants. *U6* served as control.

Using chromatin immunoprecipitation followed by deep sequencing (ChIP-seq) with our specific antibody, binding sites of H4.V were characterized (Dataset S1, three biological replicates). We identified roughly 3050 high confidence peaks of H4.V enrichment that represented majorly intergenic regions (Fig. 1i,j). We also generated T-DNA free Cas9 mediated knockout lines of H4.V (*h4.v KO* − three types of mutants) (Extended Data Fig. 4) that showed reduced ChIP enrichment at the H4.V peaks in comparison to WT plants (Extended Data Fig. 3c). Also, *h4.v KO* nuclei did not show H4.V signals in IFL microscopy, suggesting specificity of the H4.V antibody (Extended Data Fig. 3b). H4.V peaks predominantly occupied heterochromatic regions enriched with repressive H3K9me2 marks (Fig. 1k). Taken together, expression and incorporation of *Oryza* genera specific histone H4.V at specific loci suggested its role in chromatin regulation.

### H4.V promoted structurally condensed and less-stable nucleosomes

**Extended Data Fig. 5:**
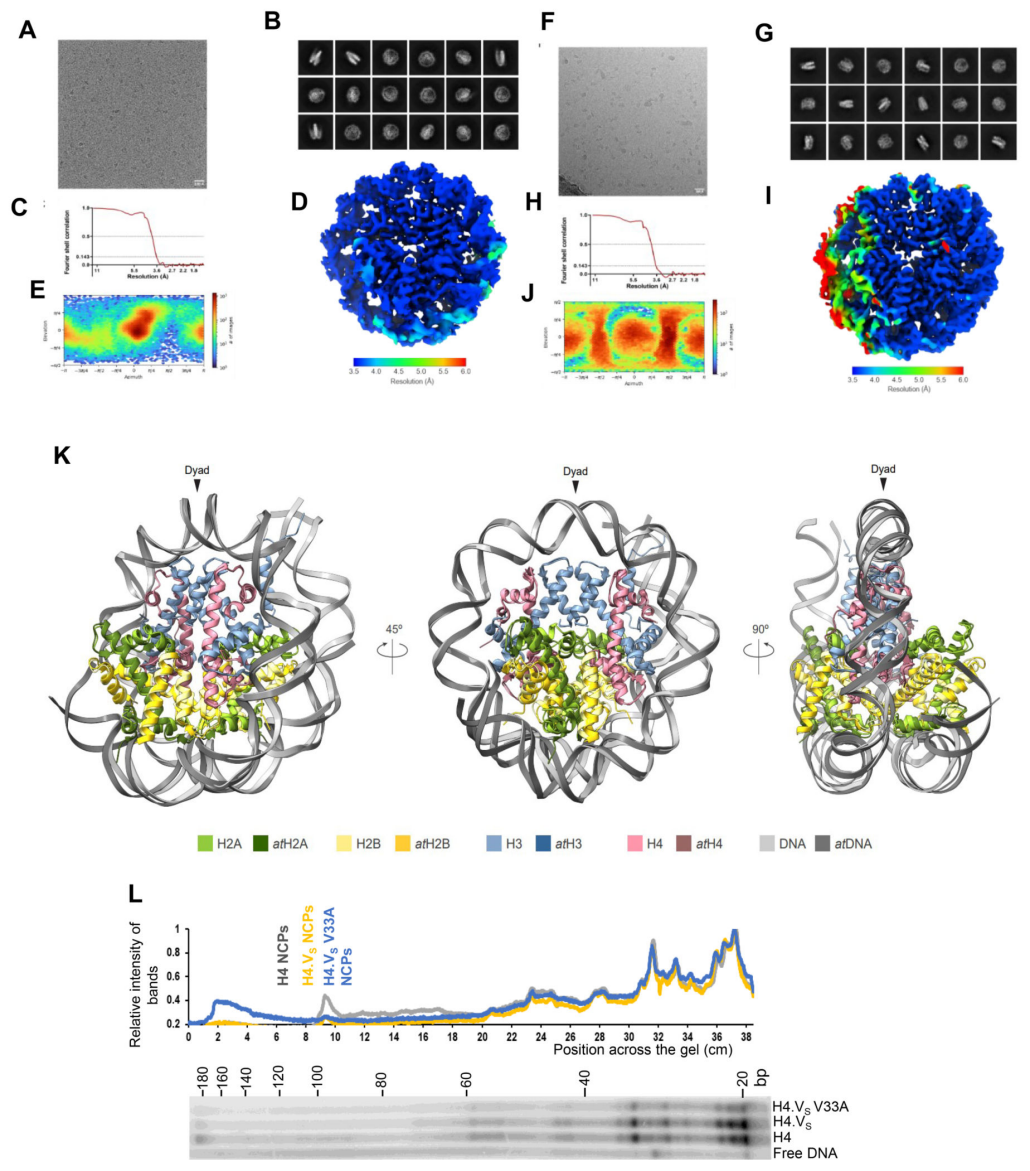
Cryo-EM data processing of NCPs and DNA interactions in rice NCPs. (A-J) Data collection and refinement for rice canonical NCP (A-E) and H4.Vs NCP (F-J). Representative cryo-EM micrographs (A, F), representative 2D classes (B, G), Fourier Shell Correlation curves (C, H) showing the resolution estimation of the maps, local resolution maps (D, J) and angular distribution of the particles (E,J) for the NCPs were depicted. (K) Overlay images of *Arabidopsis* and rice (H4) NCP depicting that the atypical DNA accessibility in plant nucleosomes is due to variations in the H2B. Magnified images depict the residue necessary for the variable DNA wrapping. (L) DNase foot-printing assay of the 188 bp NCPs with the corresponding band intensity profile showing no major distinction in the way DNA is protected by the octamers within the core of the NCPs..

**Fig. 2:**
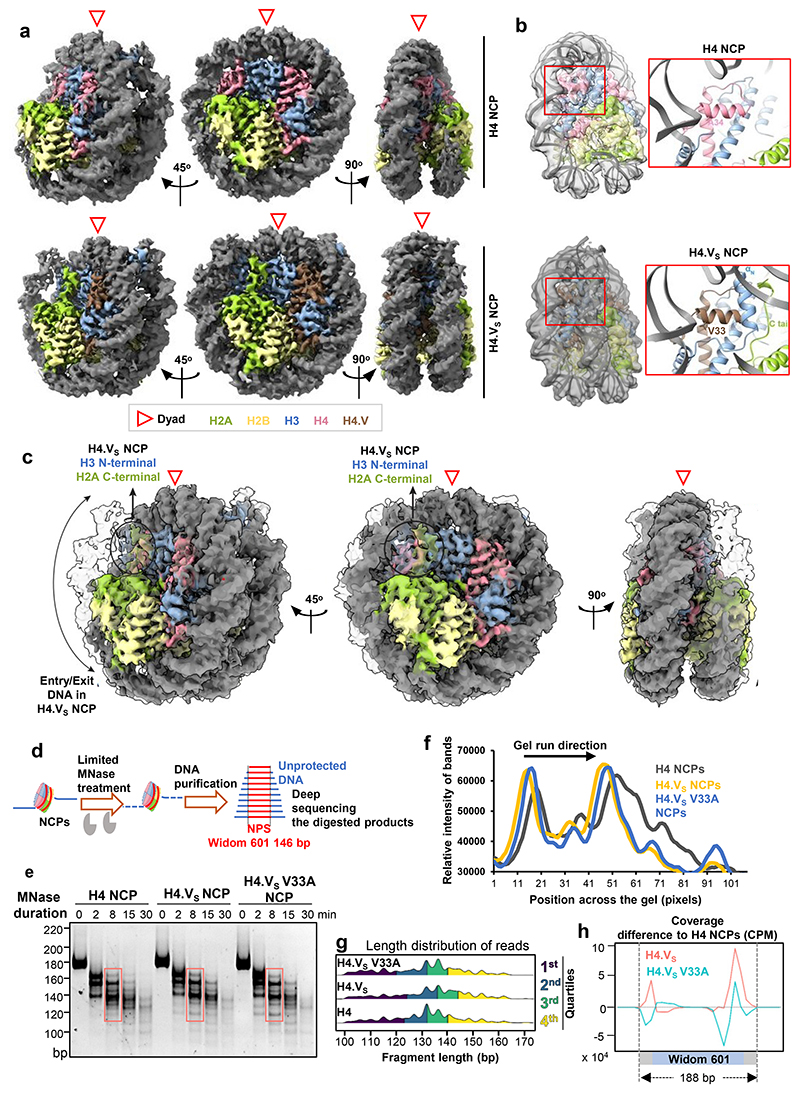
H4.V nucleosomes are structurally distinct to H4 nucleosomes. (a) The cryo-EM map of H4 NCP and H4.V_S_ NCP as visualized with Chimera X. Unlike the H4.V_S_ NCP, in H4 NCP about 20 bp of one of the entry/exit DNA regions were not resolved highlighting the contribution of H4.V_S_ in stabilizing the entry/exit DNA regions. (b) Close-up views of the H4.V_S_ amino acid variation at position 33, which contributed to the stabilization of H3 αN Helix, H2A C-terminal tail and DNA entry/exit region. (c) Representation of overlay of the H4 and H4.V_S_ NCP highlighting the region where the Valine 33 of H4.V_S_ stabilizes the H3 and H2A tails. (d) Schematic of the limited MNase digestion of the NCPs with extended (188 bp) Widom 601 sequence (NPS in red, extended DNA in blue). (e) Representative gel showing the DNA size profiles of the MNase digestion over time course. (f) Quantification of fragments highlighted in (e). (g) Length distribution of purified DNA fragments after limited MNase digestion. Quartiles of length are coloured. (h) Coverage plots (difference w.r.t. H4 NCPs) depicting distribution of differential MNase protection of DNA over the extended Widom 601 NPS for H4.V_S_ and H4.V_S_ V33A NCPs.

In order to investigate the differences brought about by the residue variations to the nucleosomes, we performed single particle cryo-EM analysis of nucleosomes reconstituted using recombinant histones with either canonical H4 or H4.V_S_ (Methods and Supplementary Table 6). Processing of the datasets using the standard CryoSPARC workflow yielded three-dimensional maps for H4 and H4.V_S_ containing nucleosomes at 3.6 Å resolution (Extended Data Fig. 5a-j). About 20 bp of DNA in NCP entry/exit positions in H4 nucleosome was not resolved (from about Super Helical Loop +5) suggesting the presence of an asymmetrically flexible DNA entry/exit regions (Fig. 2a). This observation was consistent with the recently published ^47^ *Arabidopsis* NCP structure (Extended Data Fig. 5k). Interestingly, when the structures of rice H4.V_S_ NCP and rice canonical NCP were compared, unlike H4 NCP, both entry/exit DNA regions of H4.V_S_ NCP were well resolved (Fig. 2c). DNA-octamer interaction profile within the core of the nucleosome was not drastically different between H4 and H4.V_S_ NCPs as revealed by DNase foot-printing assay (Extended Data Fig. 5l). In correlation with the stabilised entry/exit DNA regions, both H3 αN helix and the H2A C-terminal tail were well resolved in the H4.V_S_ NCP. Hence, we concluded that H4.V stabilizes the entry-exit DNA of the nucleosomes. Interestingly, H4.V has an Alanine to Valine substitution at position 33 which appears to stabilize the H3 loop connecting the H3 αN helix and α1 helix (Fig. 2b,c). It is possible that this residue plays a significant role in various structural and biochemical properties of H4.V.

In order to test if this structural feature is attributable to V33A variation in H4.V, we generated nucleosomes with H4, H4.V_S_ and H4.V_S_ V33A mutant containing octamers. We used Widom 601 NPS (146 bp) flanked by 21 bp free DNA on either side. NCPs were partially digested using micrococcal nuclease (MNase) which allowed estimation of accessibility of free DNA, and the undigested DNA from the reaction mix was deep sequenced (Fig. 2d). It was evident from the size distribution of DNA fragments that H4.V_S_ NCPs accumulated longer fragments, suggesting closer interaction of the DNA with the H4.V_S_ octamers when compared to H4 NCPs (Fig. 2e-g). As expected, H4.V_S_ V33A NCPs showed an intermediate MNase protection suggesting the contribution of that residue in wrapping the nucleosomal DNA. Further, alignment of the protected DNA fragments to the NPS revealed that the free DNA located at the termini of NPS was protected against MNase activity (Fig. 2h). Stabilization of entry/exit DNA regions observed in the H4.V_S_ NCP (Fig. 2a-c) are consistent with our biochemical finding that the H4.V_S_ NCP (due to the residue V33) is relatively more resistant to MNase digestion when compared to canonical NCP.

**Fig. 3:**
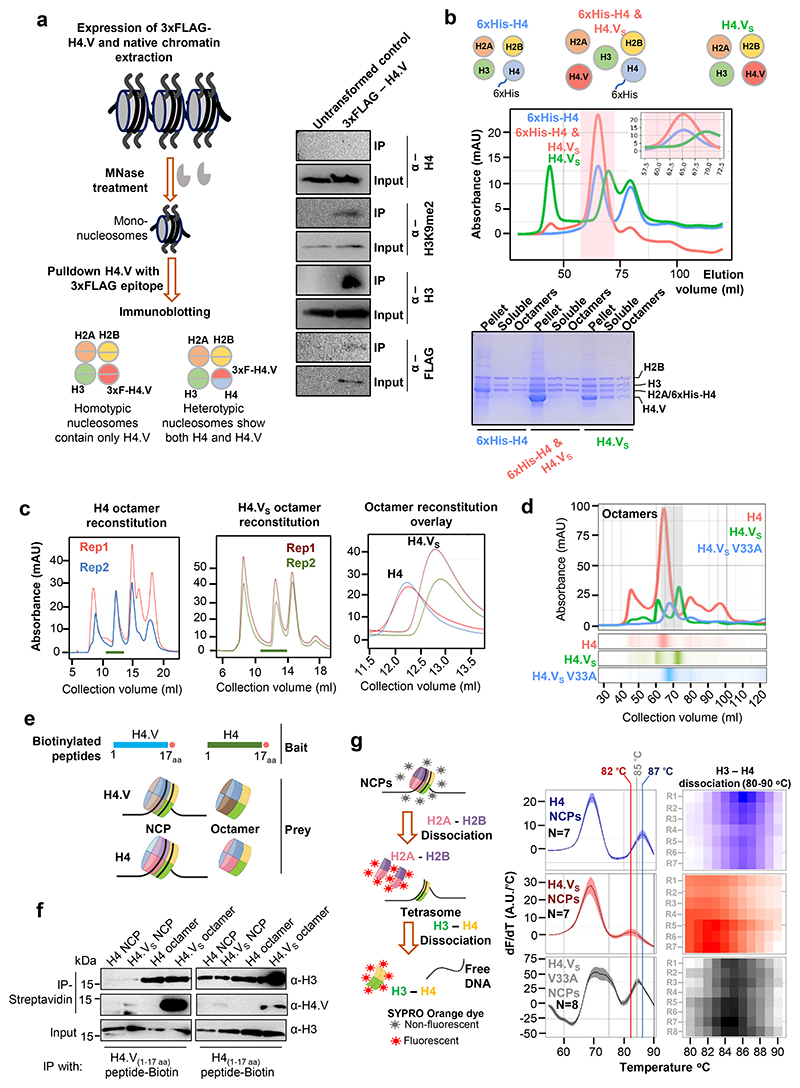
H4.V confers specific properties to nucleosomes. (a) Mononucleosome-IP assay scheme and immunoblots showing that H4.V occurs as homotypic nucleosomes *in planta*. H4.V was expressed as N-terminal 3xFLAG tagged transgene and pull downs were performed with untransformed WT as controls. (b) Histone refolding experiments with 6xHis tagged H4 and untagged H4.V_S_ in three combinations are shown. Post-refolding dialyses, pellet, soluble fraction of the dialysate and the SEC fraction corresponding to the octamers were analysed on an SDS-PAGE gel. The octamer fractions of the chromatograms are shaded in pink. SDS-PAGE gel showing the relative proportions of soluble octamers and pellet fractions. (c) Gel filtration chromatograms (24 ml S200 column) of the serially dialysed histone refolding mix containing H4 or H4.V_S_ to purify octamer fractions (green bars) from two independent experiments. Overlay of the chromatograms, zoomed at the octamer elution fraction (green bars) is shown in right. (d) Chromatogram of the octamer purification in a 120 ml S200 column with the H4.V_S_ V33A modification. (e) Schematic for the pulldown using biotinylated peptides (1-17 a.a. residues) of H4 and H4.V (bait) with the NCPs or octamers (prey). (f) Immunoblots showing pulldown products probed with α-H3 or α-H4.V. About 10% of the prey complexes were used as input control. (g) Schematic depicting the stepwise thermal decay of the NCPs. Fluorescent and non-fluorescent versions of the SYPRO Orange dye are shown in red and grey, respectively. Thermal decay rates plotted as a function of temperature. Shaded region signifies 95% confidence interval from 7 replicates. First peak at ~68 °C depicts H2A-H2B dissociation and second peak depicts H3-H4 dissociation.

Nucleosomes containing histone variants can be heterotypic or homotypic based on whether one or two units of the variant are present in each nucleosome. In order to test if the H4.V forms homotypic or heterotypic NCPs in plants, we performed mononucleosome-immunoprecipitations (IP) using transgenic lines expressing 3xFLAG-H4.V. Mononucleosome-IP assay (Fig. 3a) revealed that the H4.V is present as homotypic nucleosomes in plants as canonical H4 was not observed in the 3xFLAG-H4.V containing mononucleosomes. It is interesting to note that 3xFLAG-H4.V immunoprecipitated the H3K9me2 modified nucleosomes as seen in ChIP-seq (Fig. 1k). Further, we tested if the homotypic status of the H4.V is due to the inherent property of the variant. Towards this, we prepared 6xHis-H4 (for size distinction) and quantitatively assayed its ability to fold into octamers when stoichiometrically dialysed with H4.V_S_. In this competition assay, 6xHis-H4 effectively folded into octamers out-titrating the H4.V_S_ (Fig. 3b), suggesting that heterotypic nucleosomes with both H4 and H4.V are disfavoured inherently. This result directed us to use only the homotypic NCPs for further experiments.

In order to further probe implications of variations in the H4.V N-terminal tail, we assayed several biochemical properties of the H4.V_S_ (with and without V33A mutation) and H4 NCPs. Firstly, histone octamers (without DNA) prepared using H4.V_S_ were hydro-dynamically smaller than H4 containing octamers as they eluted slowly through a 24 ml size exclusion column (Fig. 3c). The same results were observed prominently in a 120 ml size exclusion column with intermediate effect in H4.V_S_ V33A NCP, signifying the variation of compaction between the H4.V_S_ and H4 containing histone octamers (Fig. 3d). This observation of compact H4.V_S_ octamers corroborated the cryo-EM observations of stabilisation in H4.V_S_ NCP (Fig. 2a-b).

To check if the H4.V tail variations modulated interactions between the tail and DNA or octamer, we performed a peptide pulldown assay, where the first 17 residues (comprising maximum variations) of H4.V or H4 (with a biotin label, used as bait) were allowed to interact with recombinantly purified H4.V_S_ and H4 containing octamers and NCPs (prey) (Fig. 3e). If the H4 variant tail peptide is competent in interacting with the octamer core or the DNA itself, it must pull down the prey molecules. Using H3 histone as the unbiased readout for the pulled-down species, we observed that even though H4 tail efficiently interacted with all the prey species, H4.V tail interacted only with the octamers but not with the nucleosomes (Fig. 3f). This indicated that H4.V tail residues were inefficient in surpassing the negatively charged DNA shielding to interact with the octamer core within, and pointed out a critical difference with H4 tail.

To test if the H4.V_S_ NCPs display varied degrees of stability, we traced a thermal decay profile using a fluorometric dye that quantitatively assessed the two-step denaturation of nucleosomes (Fig. 3g). H3-H4 tetramer complex of H4.V_S_ NCPs denatured at a 5 °C lower point when compared to H4 NCP tetramer (Fig. 3g) unlike the H2A-H2B dimers. We also verified that the lower denaturing point of H4.V_S_ NCPs is partly due to V33A modification, as H4.V_S_ V33A NCPs were more stable when compared to H4.V_S_ NCPs. These assays confirmed that the variations in the H4.V resulted in NCPs that are biochemically and structurally distinct in terms of their dimensions, intra- and inter-nucleosomal interactions and stability, all of which have the potential to translate to specific chromatin properties.

### Perturbation of H4.V resulted in growth and reproductive defects

**Extended Data Fig. 6:**
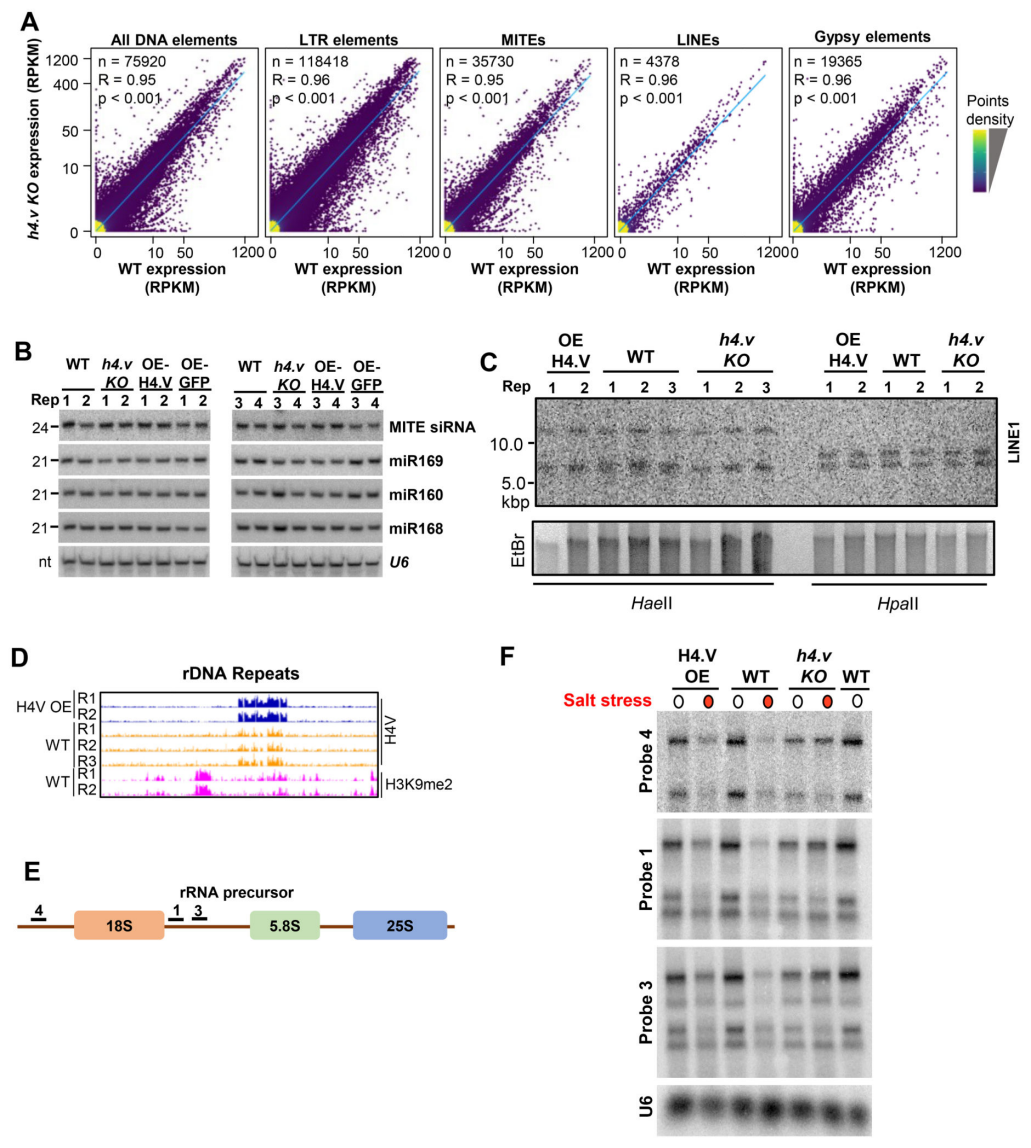
H4.V perturbation does not affect the silencing of transposons but mis-regulate rDNA expression. (A) Density scatter plots showing expression difference of different categories of transposons. Pearson correlation coefficient (R) and *p*-values are mentioned. Points density are mentioned with colour gradient. (B) sRNA northern blots showing abundance of repeat-derived sRNAs or other miRNAs. *U6* was the loading control. (C) Methylation sensitive Southern blot hybridised with LINE1 probe. EtBr-stained gel served as loading control. (D) IGV screenshots showing occupancy of H4.V at rDNA arrays in chromosome 1. (E - F) RNA blots showing the expression of rRNA precursor regions (precursor structure and probe regions marked in (E)). *U6* served as loading control.

**Extended Data Fig. 7:**
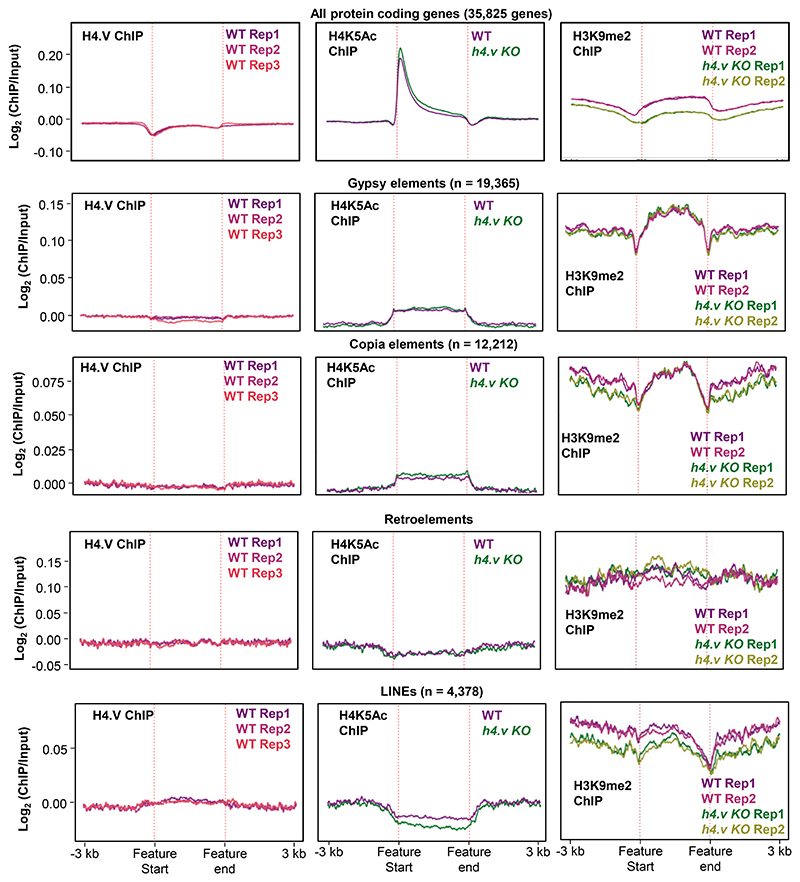
Occupancy of H4.V over protein coding genes and repeats Metaplots showing the occupancy of H4.V, H4K5Ac and H3K9me2 marks over the protein coding genes and other annotated transposons and repeats.

**Fig. 4:**
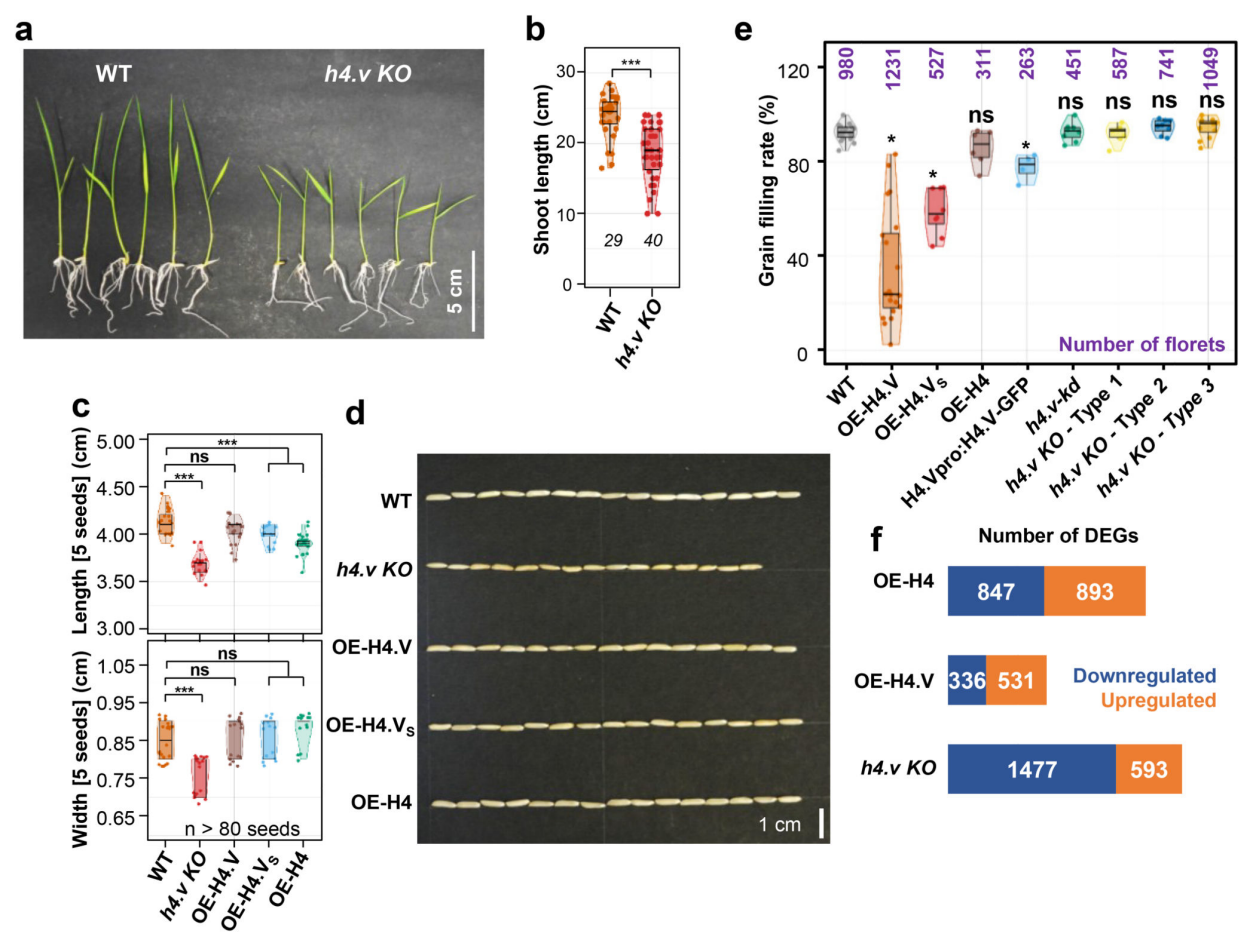
Histone H4.V perturbation leads to phenotypic defects largely attributable to gene mis-regulation. (a) Seedlings images of WT and *h4.v KO* plants. (b) Box-violin plots showing shoot length distribution of WT and *h4.v KO* plants. Number of plants taken is mentioned in italics. (c) Box-violin plots showing seed size distribution taken 5 at a time. Dots represent dimensions of 5 seeds. At least 80 seeds were taken for analysis. (d) Representative image of 15 seeds from different genotypes. (e) Box-violin plots showing percentage of filled grains. Dots represent data from individual panicles. (f) Stacked bar plots showing number of DEGs in different genotypes. (b, c and e) Two-tailed Student’s *t*-test was used for statistical comparison. (*) p-value< 0.05, (ns) non-significant.

To establish the importance of H4.V during plant development we compared wild type with mutant rice plants devoid of H4.V. Loss of function of H4.V (*h4.v KO*) showed growth defects in the early stages of development and the seedlings were stunted (Fig. 4a,b). Reproductive development of *h4.v KO* plants were comparable to WT plants but *h4.v KO* produced smaller mature seeds (Fig. 4c,d). On the contrary, over-expression (OE) of the H4.V led to reduction in seed filling, possibly due to effects of spurious incorporation of the variant in the young reproductive stages (Fig. 4e). OE-H4 did not show significant reduction in seed setting rate. Notably, OE of H4.V_S_ (N-terminal 53 amino acids of H4.V fused to C-terminal 50 amino acids of H4) showed significant reduction in seed setting rate similar to OE of H4.V suggesting variable N-terminal tail is sufficient to bring changes in regulatory properties of H4.V. Transcriptome profiling in seedlings and leaves of the *h4.v KO*, OE-H4.V and OE-H4 showed large number of mis-regulated genes in the H4.V perturbed lines (Fig. 4f). Although the H4.V occupancy sites showed overlap with the H3K9me2-rich repressed constitutive heterochromatin (Fig. 1k), a de-repression of transposons and repeats in these loci were not observed in the *h4.v KO* (Extended Data Fig. 6a), indicating H4.V might have specific roles in protein coding regions. In agreement with this, variations in neither the repeat derived small(s) RNAs nor DNA methylation status of LINE1 LTR-transposon that regulate the transcriptional silencing ^48^ were observed in sRNA northern blots or methylation sensitive Southern blot (Extended Data Fig. 6b,c). Presence of H4.V signal in the nucleolar region (Fig. 1f) prompted us to test if H4.V occupies rDNA regions. We observed occupancy of H4.V at the rDNA arrays (Extended Data Fig. 6d). We performed rRNA-precursor northern hybridisations (Extended Data Fig. 6e) and found that *h4.v KO* show significantly reduced rRNA precursor expression levels. When comparing the global levels of H4.V, H4K5Ac and H3K9me2 across all protein coding genes (PCGs) and different types of transposons/repeats, we found negligible occupancy of H4.V suggesting that H4.V occupancy is very specific and not seen in different features globally (Extended Data Fig. 7). Notably, there was no significant difference in global H4K5Ac and H3K9me2 marks upon *h4.v KO* over these features. Lack of variation in heterochromatic mark H3K9me2 over the transposons in *h4.v KO* supports lack of mis-regulation of transposons (Extended Data Fig. 7). These results suggest that although H4.V is predominantly located at heterochromatin it does not affect expression of transposons. However, it affects indirectly expression of selective protein coding genes and rDNA, resulting in the regulation of growth and reproductive development.

### H4.V prevented aberrant salt-stress like transcriptome

**Extended Data Fig. 8:**
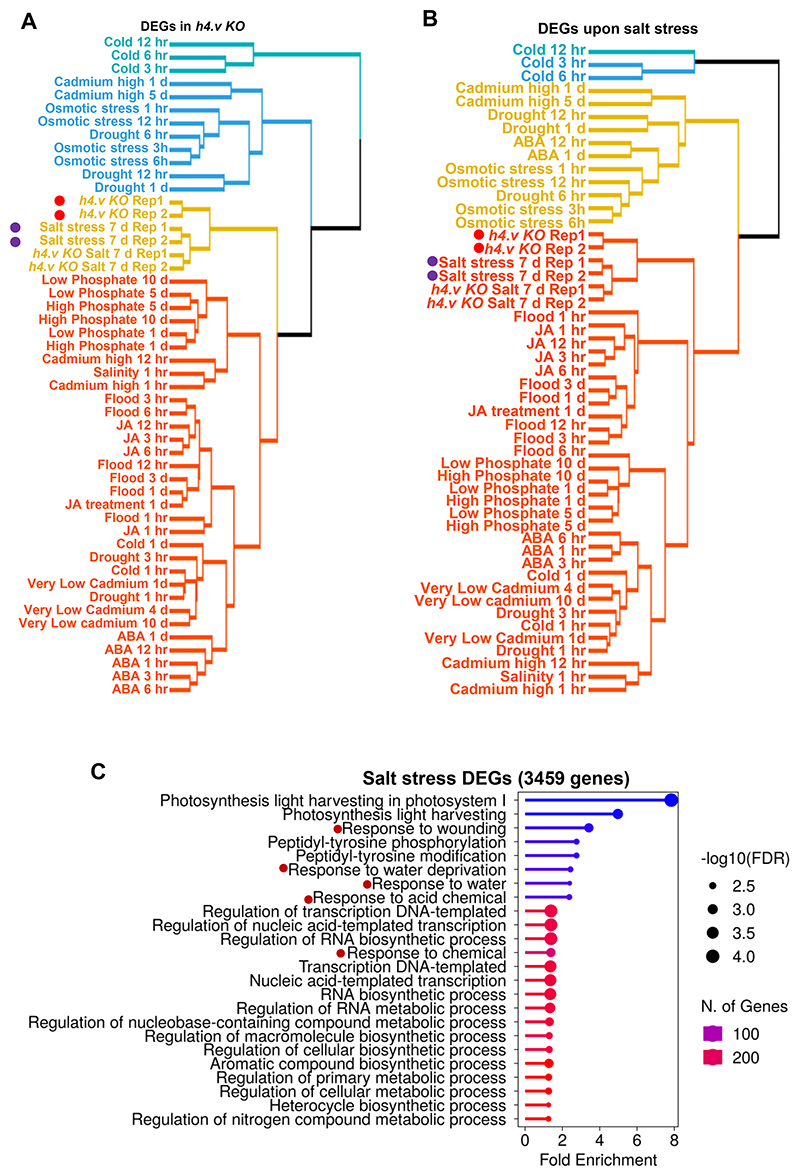
KO of H4.V exhibits a transcriptome similar to salt stressed state. (A-B) Clustered dendrograms comparing transcriptomes across different stress conditions from TENOR datasets, analyzed for the *h4.v KO* DEGs (A), or the salt stress DEGs (B). Red dots highlight the *h4.v KO* profiles and purple dots represent salt stress profiles. Figures 5C and D depict representative examples and replicates are shown here. The tree is clustered into four hierarchical types. (C) Gene Ontology enrichment analysis of salt stress DEGs. Red dots represent categories associated with salt stress responses.

**Extended Data Fig. 9:**
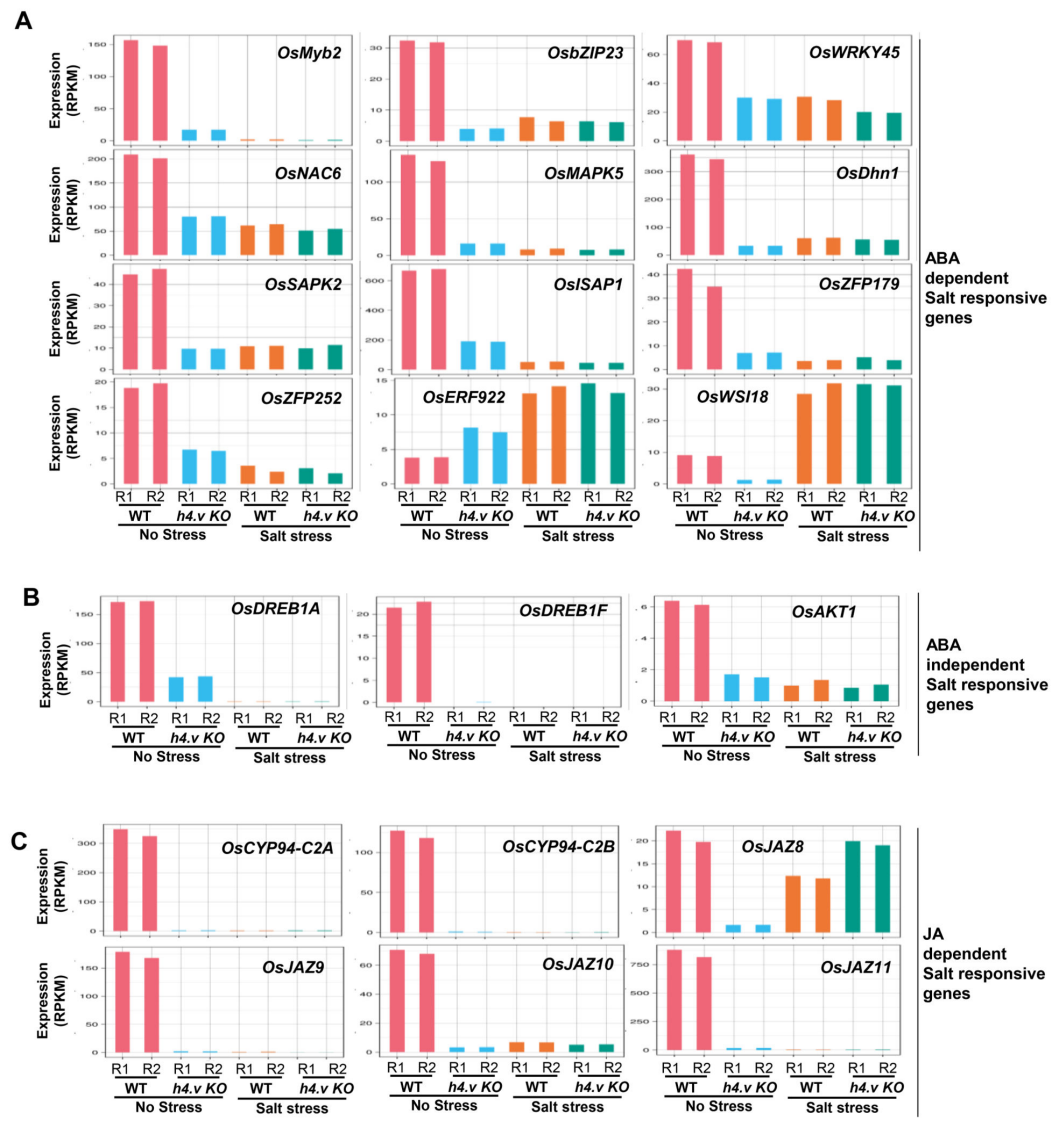
Knockout of H4.V results in misregulation of phytohormonal pathway genes responsive to salt stress. (A-C) Gene expression levels in WT and *h4.v KO* with and without salt stress. Gene names are mentioned.

**Fig. 5:**
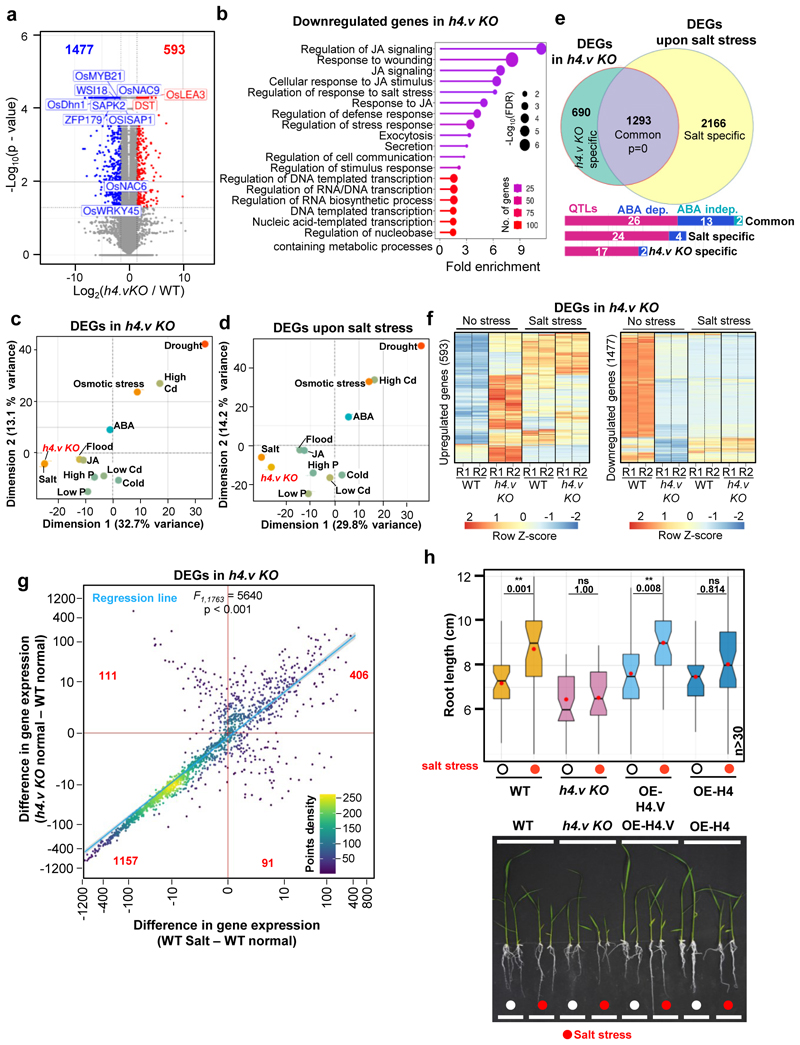
H4.V is necessary to prevent precocious salt stress like transcriptome. (a) Volcano plot showing the DEGs in *h4.v KO* seedlings. Blue dots represent downregulated and red dots represent upregulated genes. Labelled genes are stress-responsive. (b) GO enrichment categories among the downregulated genes. FDR-false discovery rate. (c-d) PCA plots of selected stress dependent transcriptomes (full list in Extended Data Fig. 8) for the *h4.v KO* DEGs (c) and salt stress DEGs (d). The first two principal dimensions are plotted and the percentage of variance explained is indicated. (e) Venn diagram representing the overlap between *h4.v KO* and salt stress DEGs. Hypergeometric test was used for statistical testing of overlaps. Bar plots show no. of genes represented in each category with salt-linked QTLs, ABA dependent/independent salt responsive genes. (f) Clustered heatmaps showing variation in expression across WT and *h4.v KO*, with and without salt stress across *h4.v KO* DEGs. (g) Density scatter plots showing resemblance of *h4.v KO* with salt stress transcriptomes over *h4.v KO* DEGs. Points density is color coded. Number of points in each quadrant is mentioned in red. Blue line represents the linear regression fit line, and grey shade represents the 95% confidence interval. Difference in gene expression is plotted and x- and y- axes are scaled to inverse sine hyperbolic function. F-test was used for statistical testing of the dependence of two perturbations (salt stress and *h4.v KO*). (h) Box plots showing root length distribution without and with salt stress (red circles) across genotypes (left panel). Red dots within the boxes represent mean of the values. At least 30 plants were phenotyped for each condition/genotype. Student’s *t*-test was used for statistical testing. p-values are mentioned across comparisons. Representative image of the plants used for phenotyping are shown (right panel).

Histone variants were implicated in stress responses in plants ^40^. Most of the stress responses in plants are mediated through regulation of specific master transcription factors, including WRKYs, MYBs (Myeloblastosis), NAC (NAM, ATAF, ATAF and CUC) etc. ^49^. Differentially expressed genes (DEGs) in the *h4.v KO* were enriched with major TFs involved in stress responses (Fig. 5a). Significantly downregulated genes were specifically enriched for the genes that respond to various stresses (Fig. 5b). In order to identify the specific type of stress that elicits a transcriptional program that resembles *h4.v KO*, we analysed publicly available datasets ^50^ and performed a principal component analysis (PCA) for the DEGs identified in *h4.v KO* (Fig. 5c). These analyses revealed that the *h4.v KO* DEGs respond most similarly to the salt stress response (Fig. 5c and Extended Data Fig. 8a). The same response was also evident in the DEGs identified upon salt stress treatment (Fig. 5d and Extended Data Fig. 8b,c). We performed salt stress on wild type and *h4.v KO* plants and observed that 65% of the DEGs in *h4.v KO* were commonly mis-regulated upon salt stress (Fig. 5e). Further, we compared the gene expression profiles of *h4.v KO* DEGs in WT and *h4.v KO* plants with and without salt stress. We observed that expression status of *h4.v KO* DEGs in *h4.v KO* resembled the status of wild type plants under salt stress (Fig. 5f). Surprisingly, salt stressed *h4.v KO* plants exhibited similar trend of gene expression as that of the stressed WT plants (Fig. 5f), suggesting absence of H4.V triggered a precocious salt-stress like transcriptional profile that persisted under salt-stress. Candidate genes that are well-established master-controllers of salt stress responses in rice ^51,52^ via various signaling modes have also been perturbed in *h4.v KO* similar to salt stress (Extended Data Fig. 9). Particularly, it is well known that abscisic acid (ABA) and Jasmonic acid (JA) are key phytohormonal pathways involved in salt stress responses ^51–53^. In *h4.v KO*, genes involved in ABA and JA metabolism and signalling were mis-regulated similar to salt stress response (Fig. 5e and Extended Data Fig. 9). In addition, it is well known that abiotic stressors suppress rRNA expression at the precursor level ^54^ and we found similar suppression of rRNA precursor levels in *h4.v KO* (Extended Data Fig. 6f). To quantitatively map the correlation between salt stressed state and *h4.v KO*, we plotted scatter plot of variation of the expression of DEGs across the two perturbations. We observed a statistically significant positive correlation effect (Fig. 5g) supporting the notion that H4.V is indeed involved in preventing salt stress like transcriptional state. As *h4.v KO* plants exhibited salt stress-like transcriptome, we subjected the H4.V perturbed lines to prolonged salt stress (Methods). Wild type rice plants, under 120 mM salt stress show extended primary roots but reduced secondary roots and stunted shoots and this behaviour is a proxy for the responses to the salt stress ^55^. Our assays showed that *h4.v KO* plants poorly responded to salt stress (Fig. 5h) in addition to being stunted in growth. In summary, our observations suggested that H4.V is necessary for controlling transcription of necessary salt-responsive TFs and genes aiding in coping with the stress.

### Salt stress induced increased H4.V occupancy in the protein coding regions

**Extended Data Fig. 10:**
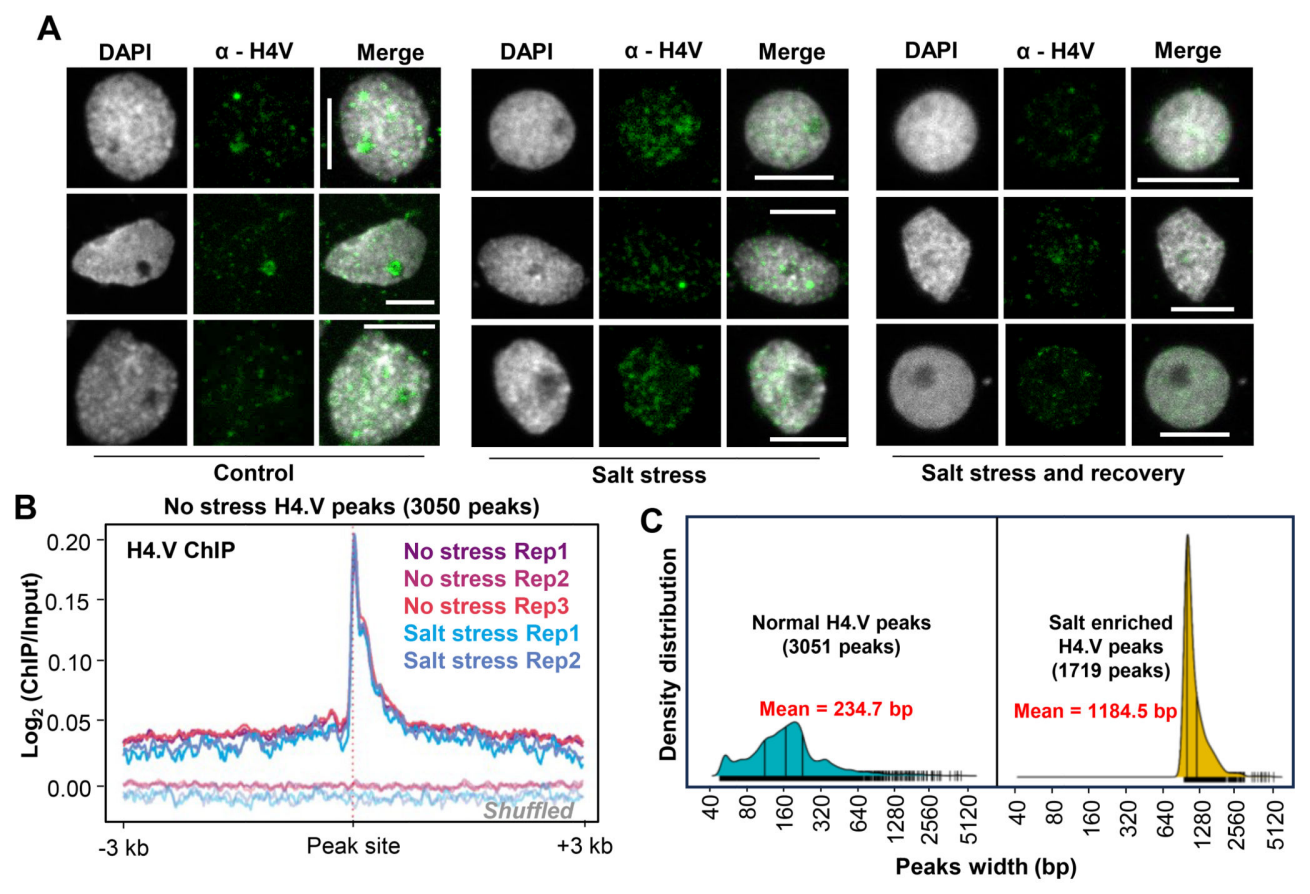
Salt-stress dependent variations in H4.V. (A) Immunostaining of H4.V showing the persistent occupancy of the H4.V upon salt stress followed by recovery. Salt stress is administered for 14 days followed by recovery. Scale: 5 μm. (B) Metaplots comparing the occupancy of H4.V with or without stress over the peaks identified without salt stress (3050 peaks). Enrichment over the shuffled set of peaks are shown in grey shade. (C) Density distribution plots of the H4.V peak widths. Mean peak width is shown in red and quartiles of size distribution is marked in black.

**Fig. 6:**
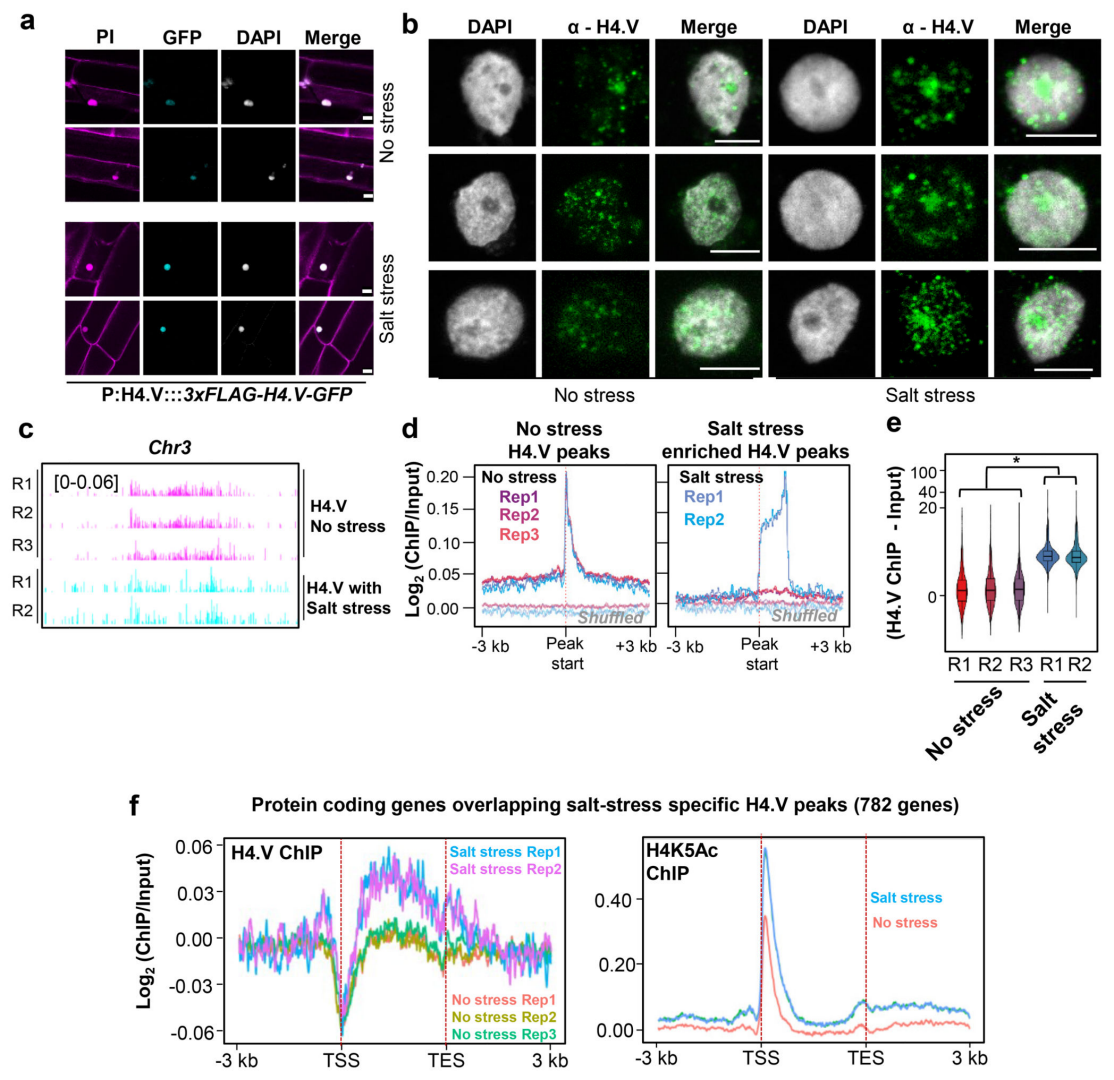
H4.V occupancy redistributes upon salt stress. (a) Fluorescent micrographs of seedling roots expressing 3xFLAG-H4.V-GFP. Seedlings grown without and with salt stress are shown. Scale: 5 μm. (b) IFL images of nuclei stained using α-H4.V antibody. Nuclei isolated from seedlings grown without and with salt stress are shown. Scale: 5 μm. (c) Chromosome-wide genome browser screenshots showing ChIP enrichment (square brackets) of H4.V without and with salt stress. (d) Metaplots showing enrichment of H4.V with and without salt stress. (e) Box-violin plots showing enrichment of H4.V over the salt-stress enriched peaks (n=1719). Y-axis is scaled to inverse sine hyperbolic function. Wilcoxon signed rank sum test was used for testing statistical significance. (*) p-value <0.001. (f) Metaplots showing the relative distribution of H4.V and H4K5Ac marks (with and without salt stress) over the protein coding genes that are occupied by H4.V upon salt stress.

To check the contribution of H4.V upon salt stress, we profiled the H4.V abundance and occupancy upon salt stress. We observed increased fluorescence signal form the H4.V-GFP translational fusion lines upon salt stress suggesting increased accumulation of the H4.V (Fig. 6a). Furthermore, spatial patterning of H4.V in the IFL micrographs revealed wider occupancy and signal in the salt stressed nuclei when compared with the unstressed state (Fig. 6b). Upon recovery of the salt stressed plants (Methods), the H4.V signals were reduced back to control levels (Extended Data Fig. 10a). To check if the H4.V occupancy genome-wide was altered upon salt stress, we analysed H4.V profiles with and without salt stress. This analysis revealed newer, stress-specific H4.V binding sites corroborating the microscopic observations (Fig. 6c). Notably, H4.V levels upon salt stress remains unchanged at the original peaks (Extended Data Fig. 10b), suggesting that salt-stress does not affect H4.V at the original sites. We also performed differential enrichment analyses of H4.V binding sites upon salt stress (Methods) which identified salt stress-specific H4.V sites (Fig. 6d and e).The salt stress specific peaks were wider when compared to the original peaks (Extended Data Fig. 10c).

**Extended Data Fig. 11:**
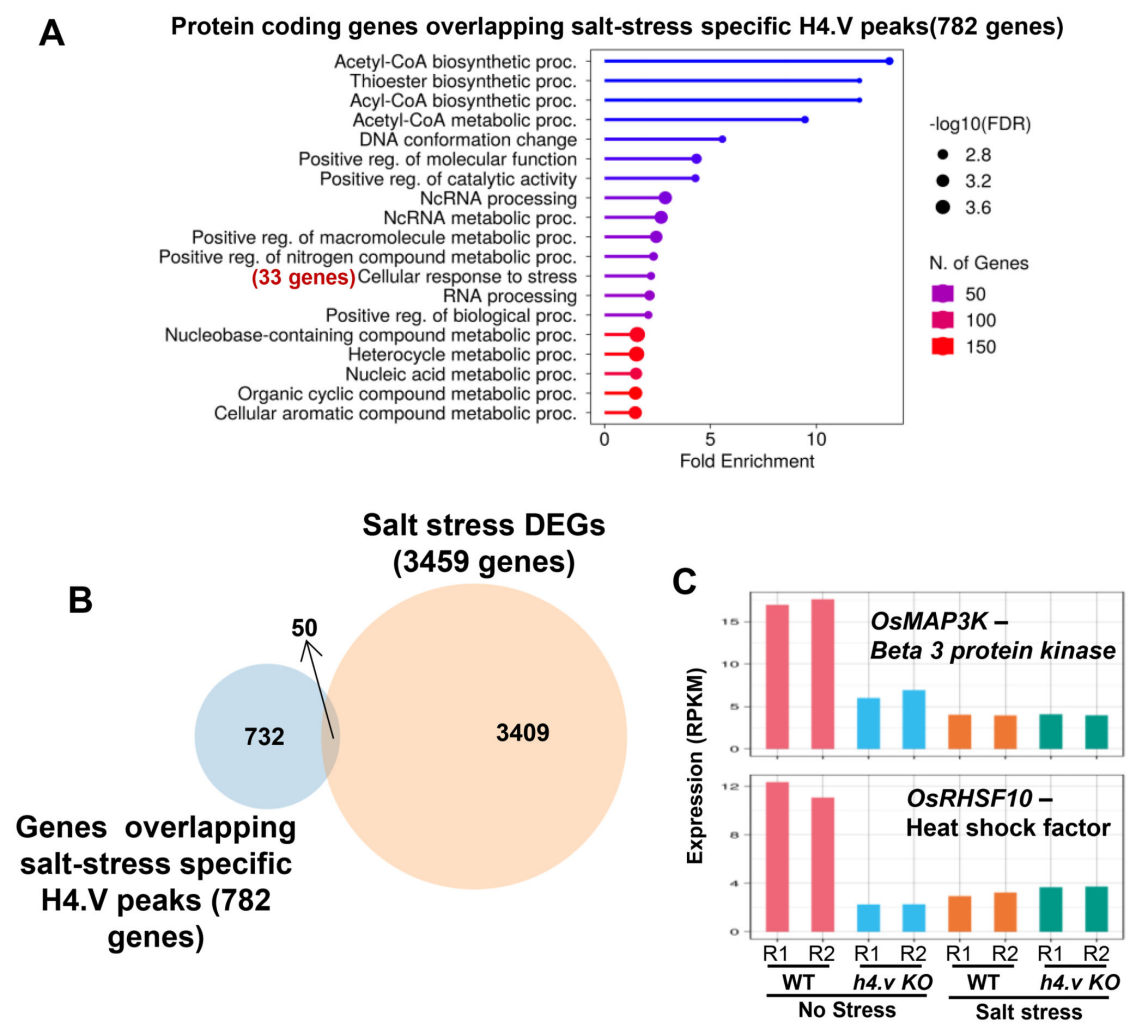
Salt-stress specific H4.V peaks occupy protein coding genes. (A) Gene Ontology enrichment analysis of salt stress DEGs. (B) Venn diagram representing the overlap of salt stress DEGs and the genes that are occupied by H4.V upon salt stress. (C) Gene expression levels in WT and *h4.v KO* with and without salt stress. Gene names are mentioned. These are representative genes that are master-regulators of stress responses that are occupied by H4.V upon salt stress.

Furthermore, salt stress-specific H4.V peaks overlapped with 782 protein coding genes. Around 33 of these genes are directly involved in stress-responses (Extended Data Fig. 11a) and they are mostly enriched for metabolic processes. Interestingly, 50 of these 782 genes were also mis-regulated upon salt stress and these include signalling proteins like MAP3K and heat shock factor RHSF10 (Extended Data Fig. 11b,c). Metagene plots of H4.V, over these genes that overlap with salt-stress specific H4.V peaks, showed that upon salt stress, H4.V occupies their gene bodies (Fig. 6f) while it is completely depleted at the TSS. On the contrary, the TSS region and +1 nucleosomes of these genes are occupied by H4K5Ac, whose levels go up upon salt stress (Fig. 6f). This modality of exclusion of H4.V and H4K5Ac is expected as H4.V exclusively forms homotypic nucleosomes. These results agree with the transcriptional mis-regulation profiles of *h4.v KO* (Fig. 5), and suggest that H4.V responds to the salt stress and its relative occupancy might modulate the gene expression pattern promoting salt dependent responses.

### H4.V affects H4K5Ac deposition in response to salt stress

**Fig. 7:**
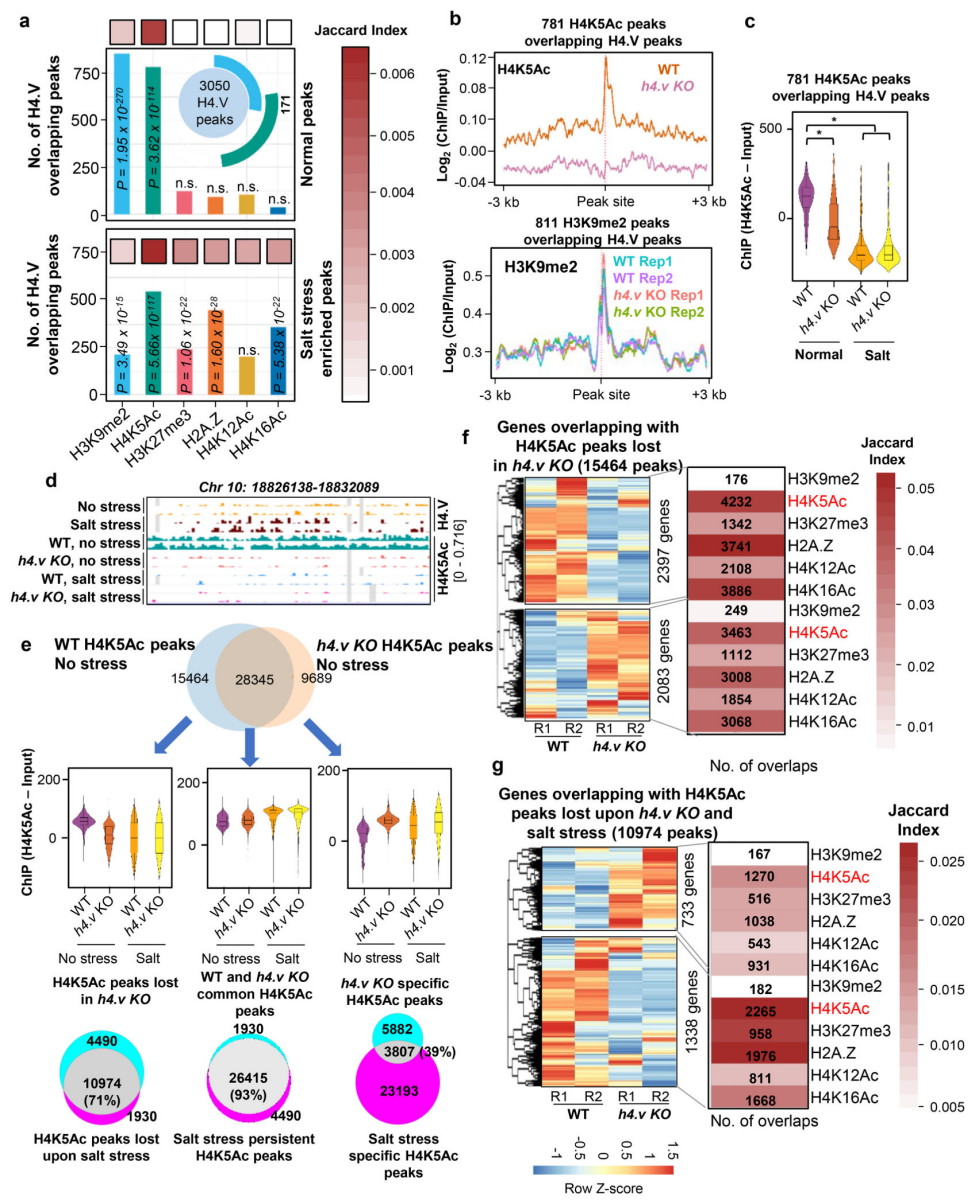
H4.V binding predisposes occupancy of H4K5Ac marks thereby regulating genes. (a) Bar plots showing number of overlaps of H4.V peaks (normal and under salt stress) with peak sets of other histone marks. Significance of overlap was tested using hyper-geometric test and p-values are mentioned. The inset plot shows number of peaks that are overlapping with H4.V and other histone marks (overlaps more than 20 are shown). Jaccard index (shown as heatmap) represents the strength of overlap. (b) Metaplot showing enrichment of the H4K5Ac and H3K9me2 marks over the respective peaks that overlap with the H4.V marks. (c) Box-violin plots depicting enrichment of H4K5Ac marks over the H4K5Ac peaks that overlap with the H4.V marks. (d) Genome browser screenshot showing ChIP enrichment (square brackets) of H4.V and H4K5Ac marks. (e) Venn diagrams and H4K5Ac enrichment boxplots for the overlaps and cross-overlaps of H4K5Ac peaks without and with stress and in *h4.v KO*. Percentage of overlap is shown. (f and g) Heatmaps showing the expression profiles of genes overlapping with perturbed H4K5Ac marks. Number of genes in each hierarchical cluster is mentioned. Adjacent heatmaps represent the number of overlaps and Jaccard indices of strength of overlap with other histone marks that co-occur with the genes.

Chromatin readouts, in terms of transcription or accessibility, is a combined effect of several layers of epigenetic modules and interaction partners. H4.V confers unique nucleosome properties (Fig. 2,3) that might facilitate or hinder other epigenetic layers by acting as guides of gene regulation. To test the link between H4.V and other chromatin modifications, we obtained several histone modification ChIP-seq datasets from rice seedlings and called for enriched peaks (Methods, Supplementary Dataset 1). We overlapped the H4.V peaks with the other histone modifications peaks identified. We observed significant overlap of H4.V peaks with distinct non-overlapping sets of H3K9me2 and H4K5Ac peaks (Fig. 7a, upper panel). Other known gene regulatory histone marks did not enrich significantly at the H4.V bound regions. Interestingly, salt stress-specific H4.V peaks overlapped significantly with stress responsive histone marks including H2A.Z and H3K27me3 (Fig. 7a, lower panel).

**Extended Data Fig. 12:**
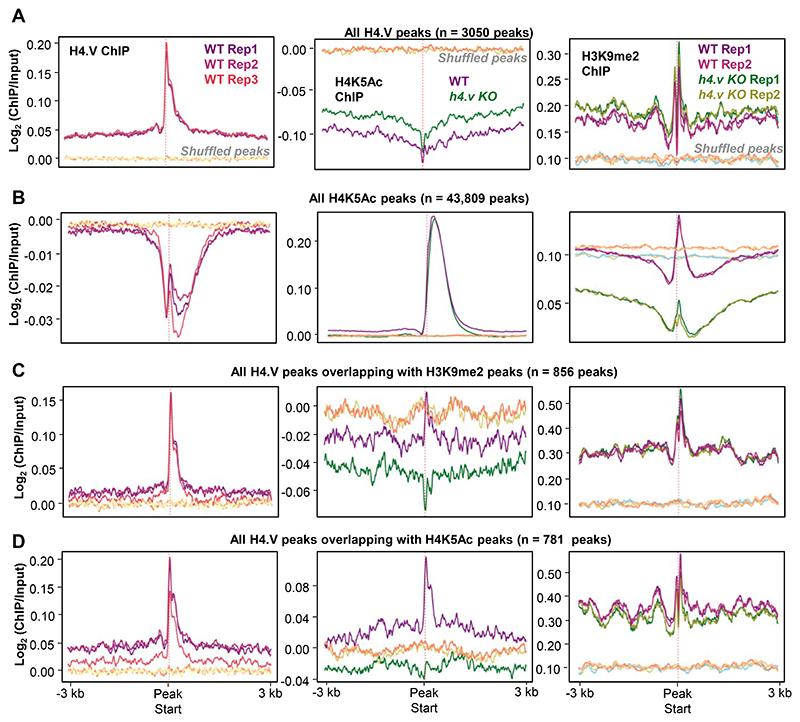
Co-occupancy and regulation of H4.V and other histone marks (A-D) Metaplots showing the occupancy of histone marks (H4.V, H4K5Ac and H3K9me2) over all H4.V peaks (A), all H4K5Ac peaks (B), H4.V peaks overlapping with H3K9me2 peaks (C) and H4.V peaks overlapping with H4K5Ac peaks (D). Enrichment over the shuffled set of peaks are shown as control for background enrichment.

It is important to note that H4.V peak overlap does not necessarily mean that these modifications co-exist on the same nucleosome. Moreover, H4.V itself is incapable of undergoing H4K5Ac modification as it lacks the 5^th^ Lysine residue (Fig. 1a) and it forms homotypic nucleosomes (Fig. 3a,b). This implied that H4.V might be regulating other histone marks proximally but not on the same nucleosome. To check the influence of H4.V, we performed ChIP-seq in the WT and *h4.v KO* seedlings with and without salt stress. H4K5Ac is a known pioneer regulator of salt stress response in plants and it is under the control of several stress-responsive histone deacetylases and acetyl transferases ^56–60^. We found that in *h4.v KO*, H4K5Ac marks were absent at the sites originally co-occupied by H4.V and H4K5Ac (Fig. 7b-d). This effect was seen only for H4K5Ac but not for H3K9me2 peaks. The loss of H4K5Ac in the sites overlapping with H4.V was specific as this was not seen in the shuffled peak sites used as controls (Extended Data Fig. 12). Upon salt stress in the WT conditions, the same sites lost H4K5Ac (Fig. 7b,d). This remarkable similarity of H4K5Ac dynamics in *h4.v KO* and upon salt stress pre-empted the notion that H4.V is necessary for the H4K5Ac modulation upon salt stress. *h4.v KO* did not cause global loss of the H4K5Ac marks and bulk of the marks were retained in the *h4.v KO* (Extended Data Fig. 12b,14).

To understand the genome-wide modulation of the H4K5Ac marks due to H4.V, we overlapped the H4K5Ac peaks in WT and *h4.v KO*. We found 35% of the H4K5Ac peaks in WT were lost in *h4.v KO* (H4K5Ac peaks lost in *h4.v KO*, 15,464 peaks) and 9689 new peaks emerged in *h4.v KO* (Fig. 7e, upper Venn-diagram), strongly suggesting the indirect effect of H4.V on the distribution of H4K5Ac marks. Surprisingly, the H4K5Ac peaks that were lost in *h4.v KO* (15,464 peaks) were also lost upon salt stress in WT plants and the newly emerged H4K5Ac peaks in *h4.v KO* (9,689 peaks) were the sites that showed H4K5Ac enrichment upon salt stress(Fig. 7e, box-violin plots). To further validate similarity of H4K5Ac profiles between salt stress and *h4.v KO*, we overlapped the differentially enriched H4K5Ac peaks upon salt stress with the mis-localised H4K5Ac peaks in *h4.v KO*. Even though salt stress specific peaks outnumbered and did not colocalize with the *h4.v KO* specific H4K5Ac peaks, 71% (10,974) of the salt stress eliminated H4K5Ac peaks colocalized with the peaks lost in *h4.v KO* (Fig. 7e, lower-left Venn-diagram). On the other hand, H4K5Ac peaks that were retained in *h4.v KO* overlapped 93% of the salt-persistent H4K5Ac peaks and showed no change upon salt stress (Fig. 7e, lower-right Venn-diagram). This data suggested that the localisation of H4K5Ac was dependent on the H4.V status and the H4.V perturbation sufficiently mimicked the H4K5Ac profile generated upon salt stress.

**Extended Data Fig. 13:**
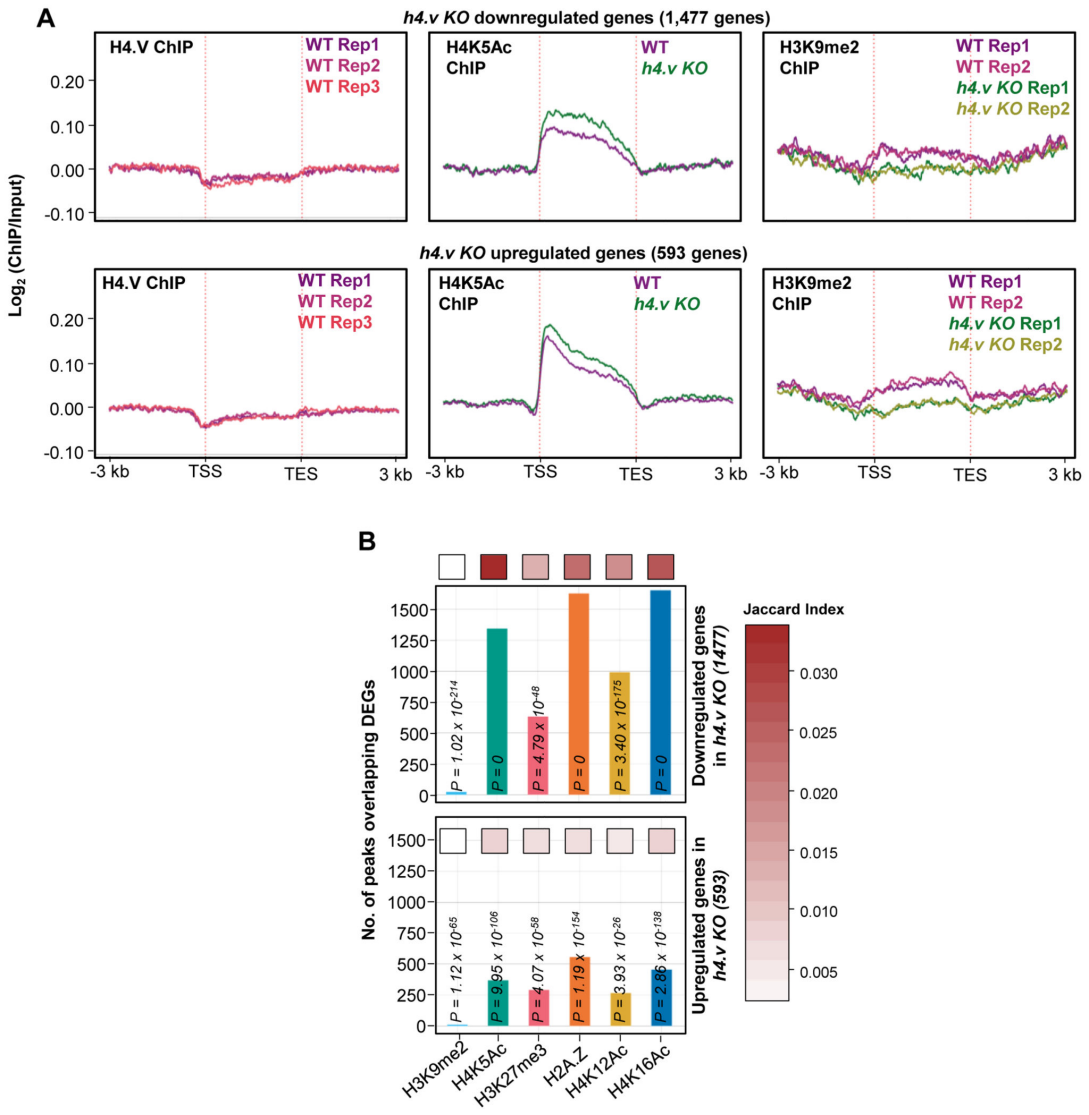
*h4.v KO* DEGs are not directly occupied by H4.V. (A) Metaplots showing the occupancy of H4.V, H4K5Ac and H3K9me2 marks over the *h4.v KO* DEGs. (B) Bar plots showing number of overlaps of *h4.v KO* DEGs with peak sets of other histone marks. Significance of overlap was tested using hyper-geometric test and p-values are mentioned. Jaccard index (shown as heatmap) represents the strength of overlap.

Since we observed a salt-stress like transcriptome in *h4.v KO* (Fig. 5), we speculated that the gene mis-expression was related to changes in the occupancy of H4K5Ac or the H4.V. Under homeostatic growth conditions, *h4.v KO* DEGs neither showed direct binding of H4.V, nor showed significant variation in H4K5Ac or H3K9me2 marks (Extended Data Fig. 13a). On the other hand, we identified the genes overlapping with the H4K5Ac peaks that were lost in *h4.v KO* (15,464 peaks overlapping 4,793 genes) and found they were mis-regulated upon *h4.v KO* (Fig. 7f). Further, of the 4793 genes, we picked the genes overlapping with H4K5Ac peaks that were also lost upon salt stress (2071 genes), which also showed similar but exacerbated response (Fig. 7g). These observations supported our model of gene mis-expression occurring via H4K5Ac marks. Taken together, these results suggested a previously uncharacterized regulator of H4K5Ac marks, H4.V, whose occupancy dictated the H4K5Ac marks and hence the gene expression necessary for salt stress responses. The above results demonstrated that the genes identified as salt stress mediators including well known TFs such as WRKY45, NACs and MYBs (Extended Data Fig. 9), regulation of which was previously ascribed to histone PTMs such as H4K5Ac ^56,61^, are also under an additional layer of control by the newly identified H4.V.

## Discussion

Histone variants are consequence of evolutionary events that resulted in novel modifications that acquired new properties. There have been several instances of independent evolution of histone variants with similar modifications in H2A and H3 in plants and animals implying the importance and utility of these modifications in conserved cellular roles ^19,21,62^. Histone variants can incorporate residue substitutions and motif additions to the canonical histones and innovate at least 4 specific downstream functions ^57^. These include a) abrogation/facilitation of PTMs, b) drive assembly of homotypic/heterotypic nucleosomes arising out of variable amino acid sequences, c) modification of the physico-chemical and structural properties of the nucleosomes and d) modulation of the spatio-temporal interactions with distant nucleosomes or chromatin regulators. Among all the histones, H4 is the slowest evolving histone as it contacts with several residues in the histone octamer complex and the H4 tail is a hotspot of several regulatory PTMs keeping it under strong negative selection ^62^. H4.V has most of the modifications in the N-terminal tail that affects PTMs but conserves the residues in the core of the histone enabling octamer’s core interactions. H4.V hence acts as an effective switch to globally change the histone PTMs. Among the H4.V specific modifications, PTM sites K5, K8, K14, K16 and K20 are abrogated and these are linked predominantly to abiotic stress responses ^39,63,64^. These modified N-terminal tail residues, if acetylated, directly modulates accessibility to DNA, thus promoting a combined histone-TF code ^65,66^.

Adding to the regulatory potential, the H4-tail residues have unique properties that contribute to the modulation of the spatio-temporal and structural interactions with other proximal or distal nucleosomes and DNA ^67,68^. A closer look at the amino acid compositions of human and rice histones showed amino acid variations in C-terminal region of rice H2A, which likely contributes to its weakened interactions with H3 αN helix as observed in human NCP ^5^. The surface charge properties of the core histone regions in the nucleosomes (like the H2A-H2B acidic patch) often mediates interactions with chromatin remodellers and histone readers/modifiers. Overall, our structural characterisations suggest that the H4.V_S_ NCP’s protection of entry/exit DNA from unwrapping and the distinct acidic patch features might contribute to modulating key interactions with chromatin remodellers and chromatin readers/writers involved in specific reprogramming of transcriptional regulation (Figure 2). It is interesting to note that a recent study uncovered that a chromatin remodeller, Decrease in DNA Methylation1 (DDM1), binds to the H4 tail residues anchoring *via* non-acetylated residues ^69,70^. Since H4.V lacks the residues that can be modified, it would be interesting to know if DDM1 can interact with H4.V NCPs and engage in epigenetic modifications. We observed atypical properties of H4.V containing nucleosomes (Figure 3) in terms of the stability, nucleosome-DNA interactions, condensed octamer core and accessibility, indicating H4.V might be contributing to major classes of alterations including interactions with remodellers and modifiers.

H4.V is found only in the *Oryza* genera among the sequenced species. Several histone variants are also restricted to specific clades as they are continuously evolving and are recent innovations necessitated by the unique environmental niches where these organisms evolve. For instance, *Hominidae* family restricted H4G and divergence of histones among kinetoplastids and Mycetazoans confer unique functional roles commensurate with the genomic divergence ^22,23,71^. Early-reproductive tissue specific exclusion of the H4.V is functionally significant as ectopic OE of the variant in reproductive tissues caused reproductive defects (Fig. 4). In the case of *Arabidopsis* seed and sperm cell specific H2B.S, when ectopically expressed in vegetative tissues, caused aberrant chromatin compaction ^16^. In addition, *h4.v KO* showed smaller seeds and stunted growth. Possible roles of H4.V in tissue specific chromatin regulation is an avenue for future investigation. Wild relatives of *Oryza* members propagate predominantly by vegetative means by having perennial rhizomes ^72^ and it is possible that exclusion of H4.V in sexual reproductive tissues is an indication of its specific role in vegetative tissues and in regulating chromatin under stresses. Whether H4.V incorporation in cell-cycle stage specific similar to canonical histones is an interesting area to explore using synchronised rice cell lines.

**Fig. 8:**
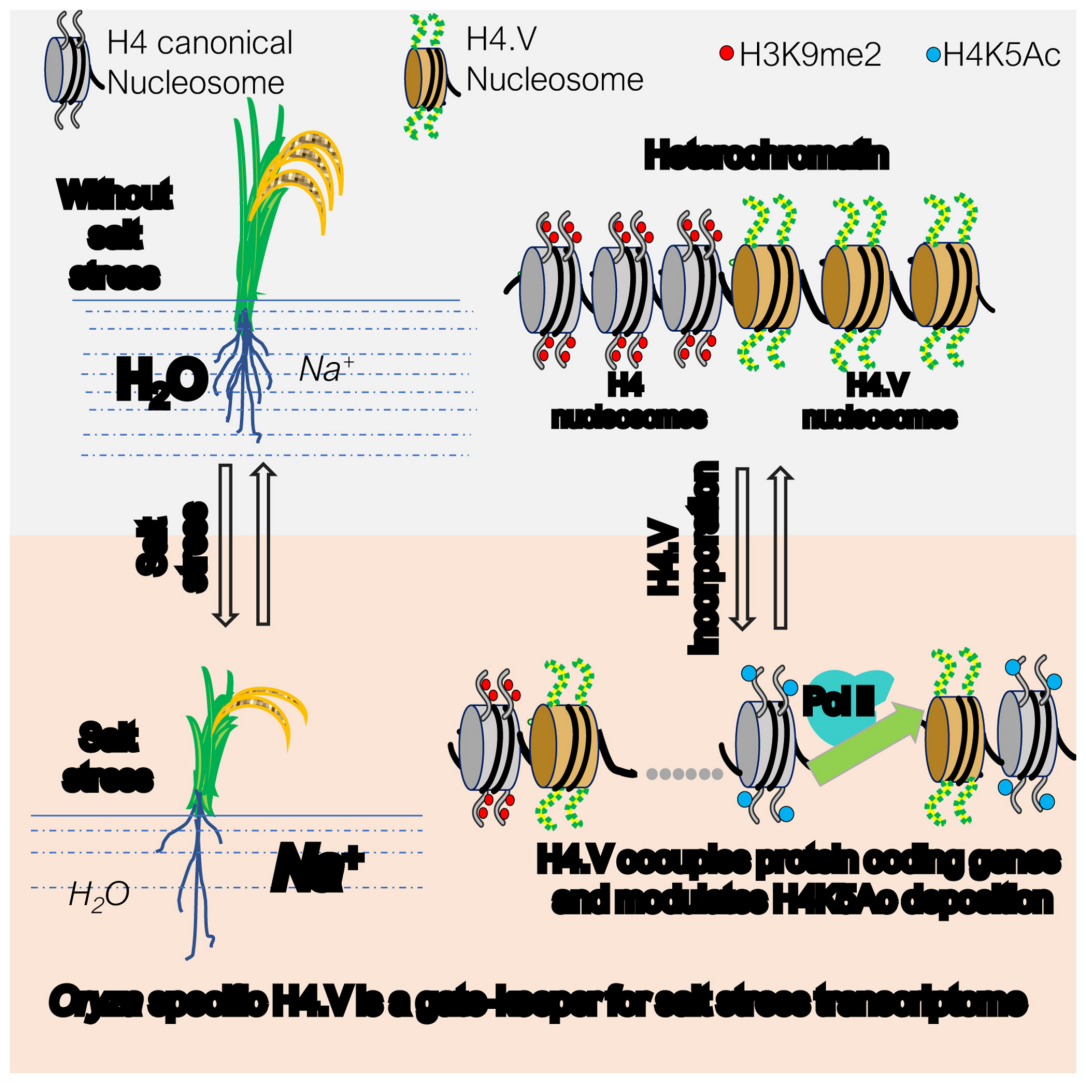
A model for the regulation of salt-stress transcriptome and H4K5Ac marks through the histone H4 variant H4.V. The rice specific H4.V naturally occupies the H3K9me2 marks enriched heterochromatic loci under normal conditions and forms homotypic nucleosomes. Upon salt stress, H4.V occupies the specific set of protein coding regions in addition to the original loci. At these new loci, H4.V occupies gene bodies and favours incorporation of H4K5Ac marks at the TSS sites, leading to salt stress specific responses.

About 20% of the arable land is already affected by salinity ^73^. Understanding the salt tolerance mechanism of crop plants that have strains or varieties with this ability in comparison with susceptible lines provides greater confidence to find a way to apply the knowledge to breed better crops. Most monocots including *Oryza* members have a mechanism mostly to exclude salt from their semi-aquatic environments. Key steps in salt tolerance mechanism include a) reduced uptake - apoplastic barriers such as casparian bands and suberin lamellae in roots b) exclusion - transporting Na+ out of the root or into the vacuoles by Na+/H+/K+ transporters; or c) tissue tolerance − osmotic adjustments by accumulation of ions, solutes and osmolytes ^52,74,75^. These steps in salt tolerance involve signaling both dependent and independent on ABA ^76^. The stunted growth phenotypes of the *h4.v KO* are likely due to the aberrant ABA signaling involved in salt like responses. The responses to salt stress are spatially and temporally distinct with shoot and root dynamically regulating transport (early) followed by metabolic biosynthesis and transport and finally structural and morphological changes. This regulated multi-pronged response requires interplay among several TFs belonging to WRKY, MYB, bZIP and NAC subtypes. These TFs can bind to hundreds of gene promoters synergistically and brings about the salt responses. In *h4.v KO*, we documented mis-regulation of NAC, MYB and WRKY type TFs that showed similar responses like that of plants under salt stress (Extended Data Fig. 9). Enrichment of H4K5Ac marks at these genes were also reduced, suggesting H4K5Ac mediated transcriptional mis-regulation poised by H4.V marking of these genes (Fig. 8). These results support the idea of evolution of H4.V as an upstream regulator of these salt exclusion mechanisms that need to be employed seasonally in a semi-aquatic species such as *Oryza* members. Such a mechanism operating at the chromatin level might have allowed successful adaptation of *Oryza* genera members to diverse water limiting conditions encountered routinely, both in their natural habitats or in the agricultural set up in which they were domesticated.

## Materials and methods

### Plant material

Unless specified otherwise, *indica* rice variety (*O. sativa indica sp*.) Pusa Basmati 1 (PB1) was used for analyses. Plants were grown in a greenhouse maintaining between 22 °C and 28 °C (ambient relative humidity) with a natural day-night cycle in black clay soil obtained from paddy field. Plants were watered daily maintaining the stagnant water levels. Other rice varieties − *O. nivara, O. rufipogon* and *O. sativa indica* variety pokkali were also grown in the same conditions.

### Cloning, plasmid construction and transgenic plant generation

For generation of CRISPR Cas9 mediated knockouts, gRNA (5’-gucuuucgcggcuacaucca-3’) coding fragment was cloned into pRGEB32 using HindIII and BsaI sites ^77^. Rice H4.V and H4 CDS were amplified (primers in Supplementary Table 4) from cDNA prepared from seedling RNA with Superscript III reverse transcriptase (Invitrogen). H4.V_S_ CDS was prepared by Gibson ligation of the PCR fragments from H4.V and H4. For the amiR-mediated *h4.v kd*, amiRNA (5’-uagacaauccgaucgugccua-3’) was encoded by swapping the *Osa*miR528 coding region in pNW55 using WMD3 tool ^78^. For OE lines and the *h4.v kd* lines, CDS or the precursor of amiR region were amplified and cloned into a derivative of pCAMBIA1300 plasmid between maize Ubiquitin promoter and 35S-terminator sequences. All the binary plasmids were mobilised into *Agrobacterium tumefaciens* strain LBA4404 harbouring extra-virulence plasmid pSB1. Embryogenic rice calli were infected with *Agrobacterium* strain containing binary plasmids using established methods ^79,80^ and the calli were selected and regenerated on hygromycin containing medium.

### Grid preparation and cryo-EM data collection

Samples were prepared as previously described in ^81^. Prior to sample vitrification, samples were concentrated up to a DNA concentration of 900 ng/μl in 10 mM Tris pH 8.0, 50 mM NaCl, 1 mM EDTA. H4 NCPs were crosslinked with 0.005 % (V/V) glutaraldehyde for 5 min. The crosslinking reaction was quenched by adding 50 mM Tris pH 8.0, allowing it to react for 1 h. Right before grid preparation, Tween-20 was added to a final concentration of 0.005 %. H4 NCPs were applied to Quantifoil R2/1 200-mesh grids coated with 2 nm of carbon (Quantifoil). H4.V_S_ were applied to unsupported Quantifoil R2/1 200-mesh grids (Quantifoil). Grids were glow-discharged for 20 s in the presence of air using a current of 20 mA. 4.5 μl of sample were used per grid. Grids were blotted for 2.5 s at 10 °C and 95 % humidity using a Leica EM GP plunger (Leica). Data for NCPs was acquired at the Cryo-EM facility of the Gene Centre in Munich. Datasets were acquired on a Titan Krios transmission electron microscope operating at 300 keV and equipped with a Falcon4i direct electron detector and a Selectris X Energy Filter (energy slit width of 5 eV) (ThermoFisher). The parameters used for data collection are provided in Supplementary Table 6. Automated data collection was done using the Smart EPU software (ThermoFisher). MotionCor2 was used to perform motion correction of the movies ^82^. Motion corrected micrographs were processed using CryoSPARC v4.0.2 ^83^. For both datasets, manual picking and further 2D classification were used to obtain 2D classes presenting defined secondary structure features. These particles were used as a template for template picking. After several rounds of 2D classification, resulting particles were used to train Topaz, which was subsequently utilised to enrich the particle dataset. Particles were extracted using a 356-px box size of (down sampled to 80-px box size). Further rounds of 2D classification were performed until 2D classes presented high resolution features. This pipeline yielded 177,136 particles for the H4 NCP dataset and 161,580 particles for the H4.V_S_ NCP dataset. Per dataset, several *ab initio* models were generated and subjected to heterogeneous refinement. Particles corresponding to the best model were re-extracted using a box size of 300 px for the H4 NCP dataset and 356 px for the H4.V_S_ NCP dataset. For the Canonical NCP dataset, two *ab initio* models were further generated and subjected to heterogeneous refinement. Particles for the best model were cleaned using 2D classification and a final model was generated using 140,877 particles by *ab initio* model generation followed by non-uniform refinement ^84^. This yielded a final 3D reconstruction at 3.62 Å resolution (Fourier shell correlation (FSC) = 0.143) for the H4 NCP dataset. For the H4.V_S_ dataset, one *ab initio* model was generated after particle re-extraction (148,277 particles). This model was refined using non-uniform refinement, which yielded a final 3D reconstruction at 3.64 Å resolution (FSC = 0.143). For both NCP datasets, the 3D Flex pipeline in CryoSPARC ^85^, was further used as a refinement tool to resolve flexible regions. This resulted in two final maps at 3.6 Å resolution (FSC = 0.143) for both the NCPs. Initial models comprising rice histone octamers were obtained using Alphafold2. NCPs were assembled combining AlphaFold2-generated rice histone octamers and Widom 601 DNA structure from human NCP (PDB: 7XD1). The models were placed in the density map obtained from the 3D Flex pipeline using the rigid fitting option available in ChimeraX. Models were refined using real-space refinement in the Phenix suite of programs. Model building was performed using Coot.

### Multiple sequence alignment and phylogeny

Histone H4 sequences (Supplementary Table 1) were aligned using ClustalW and the phylogeny was visualised using iTOL ^86^.

### Reverse transcriptase − quantitative polymerase chain reaction (RT-qPCR)

TRIzol reagent (Invitrogen) was used to extract total RNA from plant tissues and around 1.5 μg of DNAseA treated RNA was taken for cDNA synthesis using oligo-dT and random hexamers mix as primers (Invitrogen - SuperScript III RT kit). The cDNA template was used for qPCRs using SYBR green (Solis Biodyne − 5x HOT Firepol Evagreen qPCR master mix) with *GAPDH* (LOC_Os04g40950) or *Actin* (LOC_Os03g50885) as an internal control.

### sRNA northern hybridisation

sRNA northern hybridisation was performed as described earlier ^87,88^. Around 8 μg of total RNA was electrophoresed on a denaturing gel, blotted onto Hybond N+ membrane (Cytiva) and UV crosslinked. The membrane was hybridised with radioactively labelled probes (T4 PNK, M0201, New England Biolabs (NEB)) in Ultrahyb buffer at 35 °C and exposed to a phosphor screen that was scanned using Typhoon scanner (Cytiva).

### rRNA precursor northern hybridisation

rRNA northern hybridisation was performed as described earlier ^48^. Briefly, 20 μg of total RNA was electrophoresed on a MOPS-formaldehyde denaturing gel, blotted onto Hybond N+ membrane (Cytiva) using capillary transfer and UV crosslinked. The crosslinked membrane was hybridised using rRNA-precursor specific oligos ^54^ end-labelled using [γ-P32]-ATP in ultrahyb-buffer (Invitrogen) at 42 °C. After scanning the phosphor screen using Typhoon scanner, the blots were stripped and rehybridised with subsequent probes.

### Southern hybridisation

Southern hybridisation was performed as described earlier ^89,90^. Probes were PCR amplified and internally labelled using Rediprime labelling kit radioactively (Cytiva).

### Antibody generation

Polyclonal antibodies against H4.V were raised against a peptide CAPRSVAISGRGTSGA in rabbit and antigen-affinity purified by Lifetein LLC, USA.

### IFL and microscopy

IFL microscopy was performed as described earlier ^19,48^. Crosslinked seedlings were chopped and isolated nuclei were crosslinked on a positively charged slide. The nuclei are stained using α-H4.V (1:100), α-H4K5Ac (Merck 07-327, 1:200) and α-H3K9me2 (Abcam ab1220, 1:100) antibodies and detected using fluorescently tagged secondary antibodies (Invitrogen, goat raised anti-mouse 488 and Anti-rabbit 555). Nuclei were counterstained with DAPI and imaged on a confocal microscope (Olympus FV3000).

### ChIP-seq and sequence analysis

ChIP was performed from 1.5 g crosslinked rice seedlings as described earlier ^48,91^. Sheared chromatin was incubated with 10 μg of α-H4.V or 5 μg of α-H4K5Ac (Merck 07-327) or 4 μg of α-H4 (Abcam ab10158) overnight at 4 °C and further incubated with protein-G Dynabeads (Thermo Fisher Scientific) for four hours. The purified IP products were prepared into a library using NEBNext Ultra II DNA library prep kit (NEB E7103) using manufacturer’s protocol. The libraries were sequenced on an Illumina HiSeq 2500 or NovaSeq 6000 (Supplementary Table 2).

Cutadapt trimmed reads were mapped to IRGSP1.0 genome using Bowtie 2 ^92,93^ with the following parameters: -v 1 -k 1 -y -a -best -strata. Alignments depicting PCR duplicates were removed and the alignments were converted to enrichment (log_2_) coverage tracks normalising to sheared input DNA using deepTools ^94^. Coverage signals over regions of interest were computed using ComputeMatrix (deepTools) and plotted using custom ggplot2 scripts in R ^95^. ChIP peaks were called using MACS2 ^96^ with broad peak calling with respect to the input DNA control and significant peaks with 1.5 fold enrichment were considered as true peaks. Peaks obtained were analysed and merged across replicates using BEDTools ^97^. ChIPseekeR ^98^ was used to annotate the peaks, overlap Venn diagrams were generated using Intervene ^99^. ChIP-seq datasets that are publicly available (Supplementary Table 3) from previous studies were analysed in the same way and the peaks called from ChIP datasets are in Dataset S1. Differential enrichment of histone peaks across genotypes and conditions were performed using Diffreps ^100^ with the parameters described earlier ^48^ (Dataset S1).

### Recombinant histone purification and nucleosome reconstitution

Rice histones purification, octamer reconstitution and nucleosome titration were done as described previously with following modifications ^101,102^. Rice histones CDS were codon optimized and synthesised (Thermo Fisher Scientific). The histone sequences, inducible expression plasmids, *E. coli* expression strain, expression conditions for each histone and Widom 601 NPS (147 bp and 188 bp) are provided in Supplementary Table 5. The NCPs’ quality was estimated by electrophoresing on a native 6% acrylamide gel in 0.5x TBE buffer.

### NCPs MNase digestion, sequencing and analyses

For MNase digestion, 7 μg (DNA mass) of NCPs (with 188bp NPS) or free DNA was incubated with 5 Kunitz units of MNase (NEB M0247) in a reaction buffer (30 mM Tris pH 8.0, 5 mM CaCl2, 1.5 mM DTT, 0.1 mg/ml BSA) at 37 °C in a reaction volume of 60 μl. Reaction was allowed to proceed for the mentioned time points and from the reaction mix 15 μl was withdrawn periodically and mixed with equal volume of 2x deproteinization buffer (20 mM Tris pH 8.0, 80 mM EDTA, 80 mM EGTA, 0.25% SDS, 0.5 mg/ml proteinase K) to stop the reaction. About 5 μl from this sample was electrophoresed on a 10% Acrylamide gel and stained with EtBr for visualisation. The remaining digestion products were purified and libraries were prepared like ChIP-seq samples and deep sequenced on Illumina NovaSeq 6000 in a 2x100 bp format. The obtained reads were adapter trimmed (Cutadapt), the overlapping paired end reads were stitched using FLASH ^103^ and aligned to 188 bp NPS using Bowtie 2. Subsequent analyses and coverage plots were performed like ChIP-seq.

### Mononucleosome-IP assay

Chromatin digestion to mono-nucleosomes using micrococcal nuclease and subsequent immunoprecipitation was performed as described earlier ^15^.

### Histone peptide pull-down assay

MyOne Streptavidin beads (40 μl per reaction) (Invitrogen, 65001) and 25 μg of biotinylated peptides (1-17 amino acids) were allowed to bind in a binding buffer (10 mM Tris pH8.0, 50 mM NaCl and 5 mM MgCl_2_) and subsequently washed in the same buffer. Peptide-bound beads were allowed to interact with 60 pmoles of NCPs (DNA estimation) in 0.5 ml binding buffer for 6 hr at 4 °C. For the octamers, 250 pmoles of complex was allowed to interact in binding buffer with 2 M NaCl. Post-binding reaction, the beads were washed 4 times with corresponding binding buffers and eluted at 99 °C in 50 μl Laemmli loading buffer. 35 μl of the precipitated products were immunoblotted.

### RNA extraction and transcriptome profiling

Total RNA was extracted using TRIzol from 14 d old seedlings and poly(A) enriched before library preparation (NEB E7490 and E7765). Libraries were sequenced on Illumina Hiseq 2500 in a 2x100 bp format. The obtained reads were adapter trimmed using Trimmomatic ^104^ and mapped to IRGSP1.0 genome using HISAT2 ^105^.

Cufflinks ^106^ was used to perform transcript abundance estimation and differential gene expression analyses. The DEGs were called with a p-value cut-off of 0.05 and absolute log_2_ (fold change) expression cut-off greater than 1.5 (Dataset S3). Gene expression was quantified using BEDTools multicov and normalised to RPKM. Gene ontology enrichment was performed using ShinyGO ^107^.

### Stress treatment and phenotyping

For all the analyses and phenotyping at least 30 plants were used. Seeds obtained from genotype-confirmed T2 plants were sterilised using ethanol, bleach and 0.1% HgCl_2_ and germinated on 1/2 MS media with 0.3% Phytagel (Sigma Aldrich) and for salt stress 120 mM NaCl was supplemented. Seedlings were germinated in dark for 5 days and then grown in light for further 14 days before phenotyping.

### NCPs stability assay

NCP stability assay using SYPRO orange dye was performed as described earlier ^105^. Fluorescence was measured on a BioRad CFX96 real time system in a FRET mode following manufacturer’s protocol and the dF/dT computation was normalised to base fluorescence at 26 °C.

### Immunoblotting

Total nuclear protein from 0.4 g rice seedlings were extracted as described earlier ^106^. The proteins were electrophoresed on a 14% SDS-PAGE gel and blotted onto Protran supported nitrocellulose membrane as described ^108^. The membrane was hybridised with α-H4K5Ac (Merck 07-327, 1:2500), α-H3 (Merck 07-10254, 1:20000), α-H4 (Abcam ab10158, 1:2500) or α-H4.V (Custom generated, 1:1000) in a 5% milk containing 1x TBST buffer. For the bacterial proteins, 25 μl culture of 2.0 OD induced cells were lysed in equal volume 2x Laemmli buffer.

### Multi-stress transcriptome dataset analyses

Gene expression datasets were downloaded from TENOR ^50^ website (https://tenor.dna.affrc.go.jp/). Fold changes in seedling-shoot gene expression was calculated for all kinds of stresses by normalising to the corresponding control dataset. The fold change matrix was subset for the DEGs of interest, merged with the similarly processed *h4.v KO* and salt stress datasets and the non-zero entries were taken for principal component analyses using prcomp package in R. The data visualisation was done using the tools of factoextra package.

## Extended Data

**Extended Data Fig. 14:**
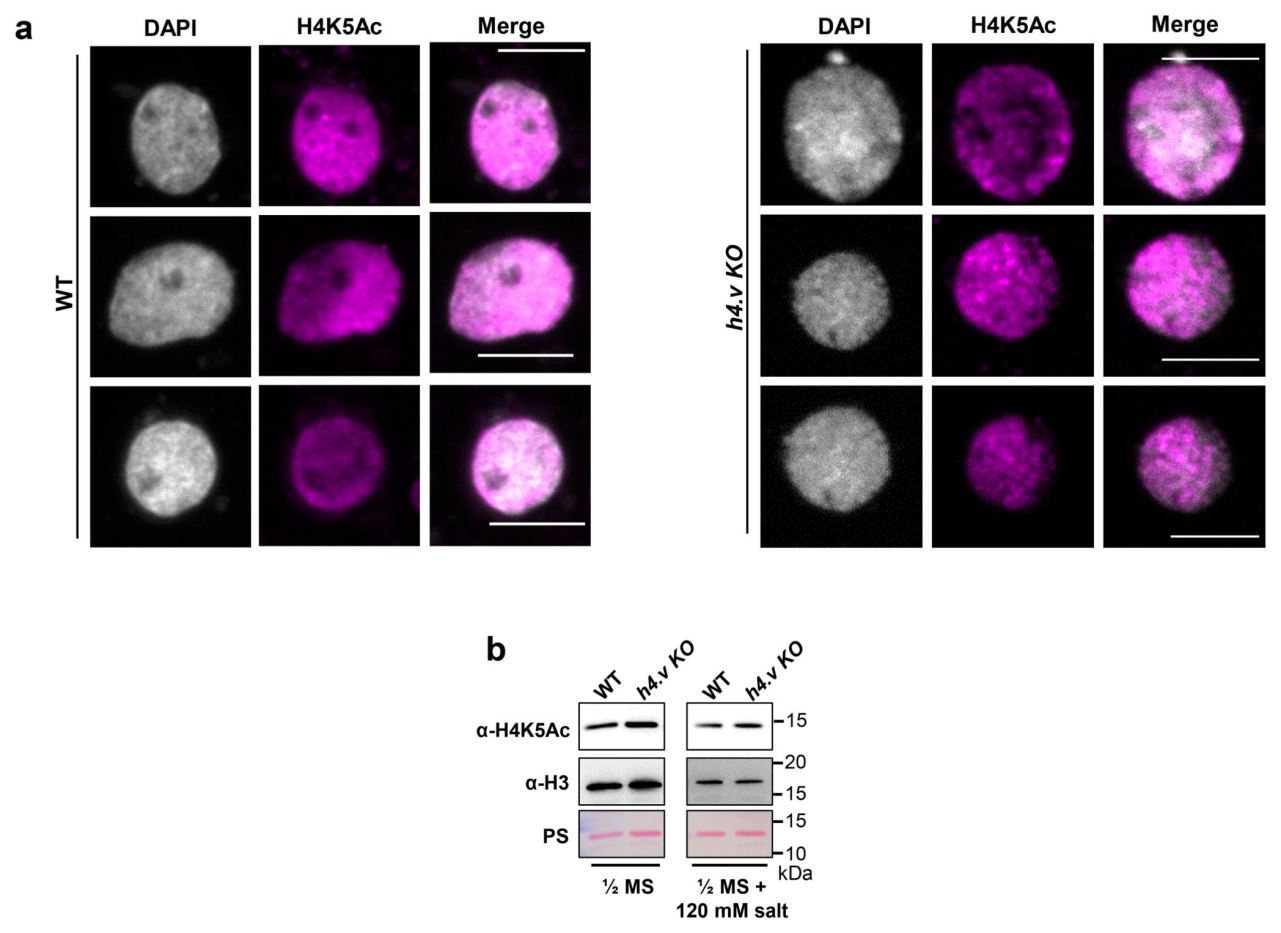
Perturbation of H4 variant does not lead to global changes in H4K5Ac levels. (a) IFL images showing occupancy of H4K5Ac marks in WT and *h4.v KO* seedlings nuclei. Scale: 5 μm. (b) Immunoblots showing the global levels of H4K5Ac marks without and with salt stress. H3 levels and Ponceau stained blots served as loading controls.

## Supplementary Material

Extended Data / Supplementary figures

Supplementary Tables

Supplementary Information

## Data Availability

All raw and processed sequencing data generated in this study have been submitted to the NCBI Gene Expression Omnibus (GEO; https://www.ncbi.nlm.nih.gov/geo/) under accession number GSE229604. Coordinates and the cryo-EM maps for the rice H4 NCP and H4.V_S_ NCP have been deposited in the Protein Data Bank (PDB) and Electron Microscopy Data Bank (EMDB). Accession codes are: H4 NCP (PDB:8Q15, EMDB: EMD-18060) and H4.V_S_ NCP (PDB:8Q16, EMDB: EMD-18061).

## In Brief

*Oryza* genera specific histone H4 variant predisposes chromatin for stress responses
