# Supplementary material for: Oryza genera-specific novel Histone H4 variant predisposes H4 Lysine5 Acetylation marks to modulate salt stress responses
Source: Nat Plants. Author manuscript; Available in PMC 2025 May 14. (PMC7617672; doi:10.1038/s41477-025-01974-2)
Supplement: Extended Data / Supplementary figures [file EMS204895-supplement-Extended_Data___Supplementary_figures.pdf]

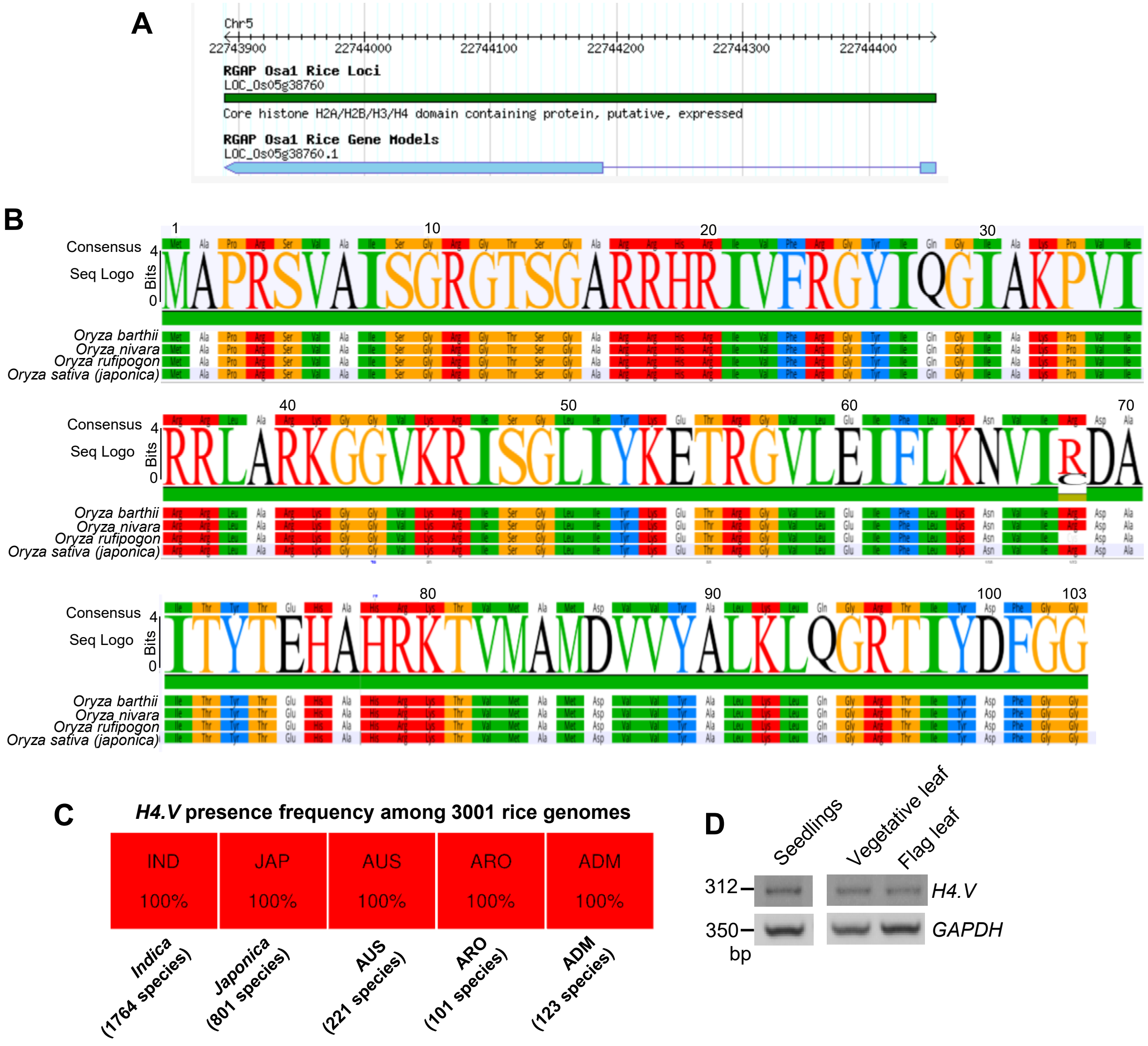

Extended Data Fig. 1. H4.V is conserved among *Oryza* genera members.

(A) Gene model of the H4.V from RAPDB (https://rapdb.dna.affrc.go.jp/).

(B) Seq-logo showing conservation of the H4.V among rice species.

(C) H4.V presence frequency among the 3001-rice genome project from rice pan-genome browser (https://cgm.sjtu.edu.cn/3kricedb/index.php).

(D) RT-PCR amplification of the H4.V transcript across three rice tissues. *GAPDH* served as control.

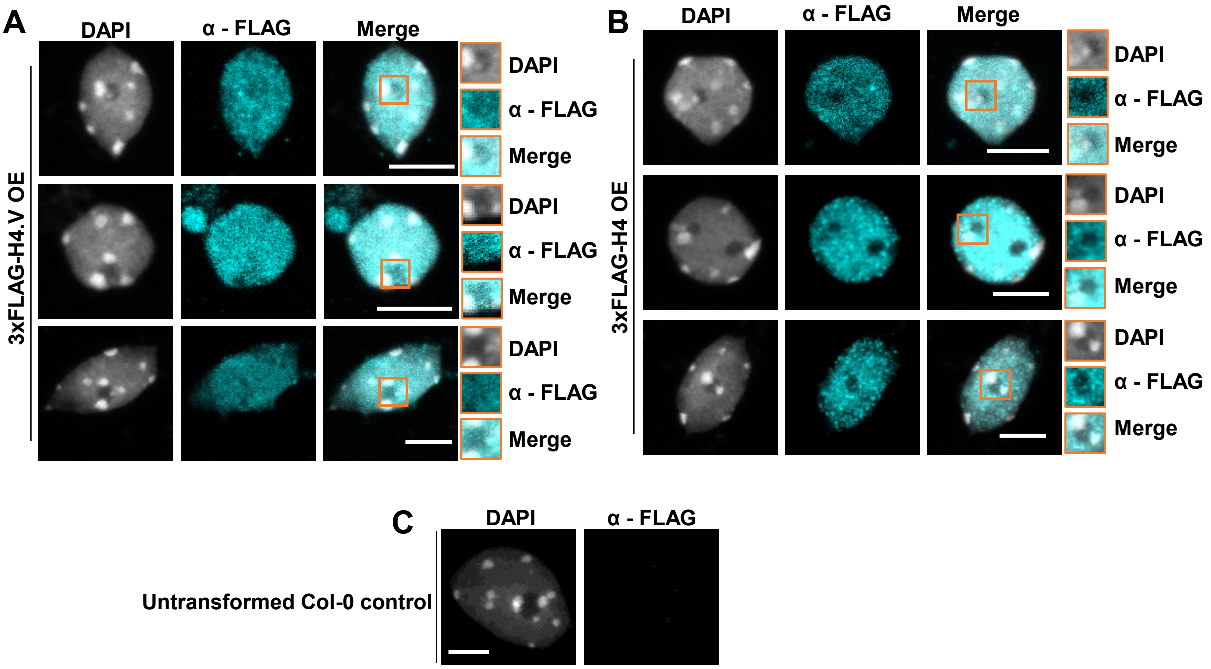

Extended Data Fig. 2. H4.V can be incorporated into the *Arabidopsis* chromatin.
(A-C) Immunostaining of nuclei from *Arabidopsis* Col-0 plants heterologously over-expressing (CaMV 35S promoter) H4.V (A) or H4.V_S (B). Nucleolar regions are shown as insets. (C) Untransformed Col-0 control shows specificity of α – FLAG antibody in immunofluorescence imaging. Scale: 5 µm.

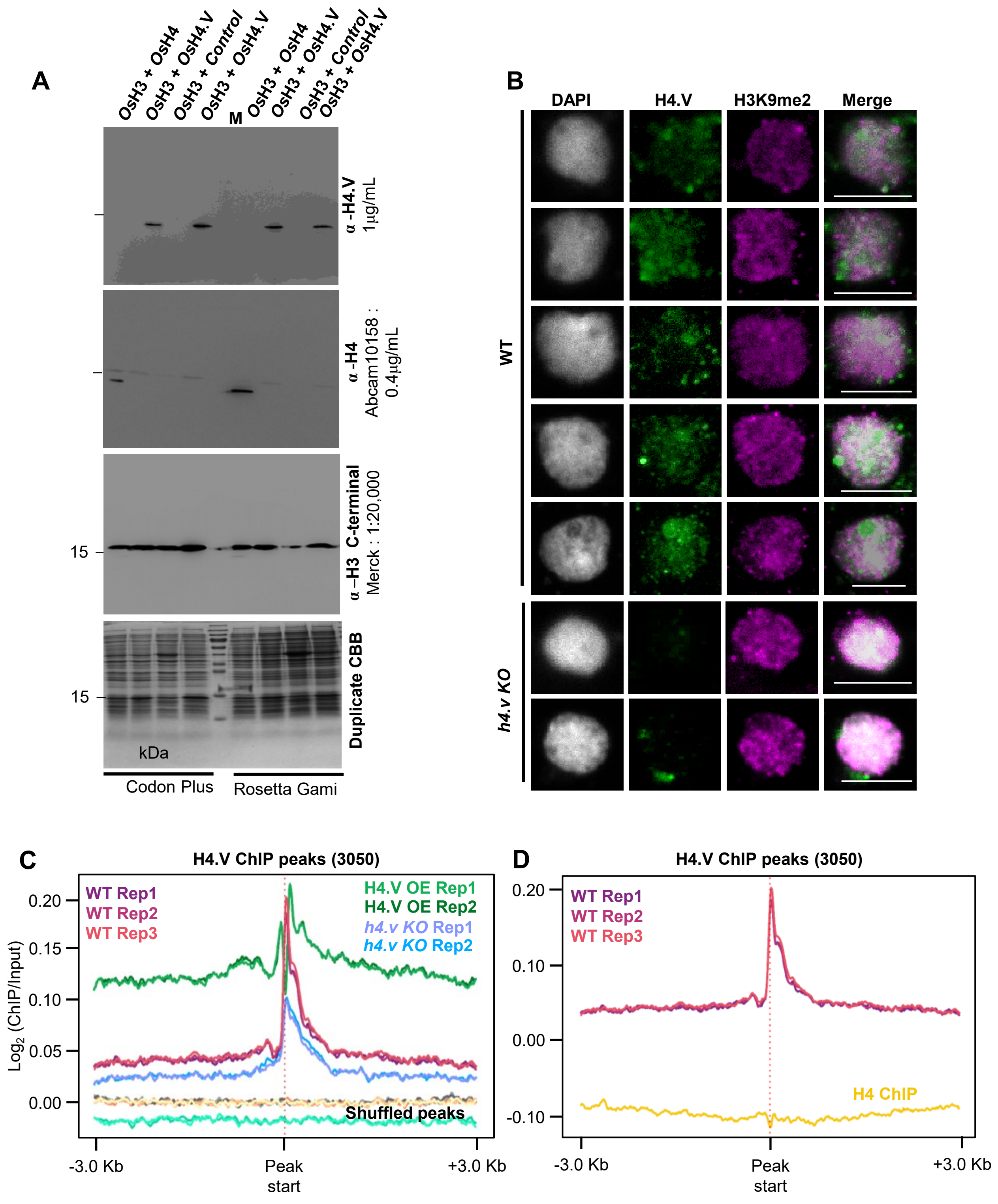

Extended Data Fig. 3. H4.V antibody is specific and does not cross react with H4.
(A) Immunoblots showing reactivity of α-H4.V and α-H4 specifically to their epitopes. Total bacterial lysates from two different *E. coli* strains are shown. Blots were stripped and re-hybridized. CBB stained duplicate gel served as loading control. Both H3 and H4/H4.V were co-expressed in the same plasmid (Supplemental Table S5). M: size marker.
(B) IFL images of nuclei from WT and *h4.v KO* plants stained using α-H4.V and α-H3K9me2. Scale: 5 μm.
(C) ChIP enrichment profiles of H4.V at H4.V peaks from WT, OE-H4.V and *h4.v KO*. Enrichment profiles at shuffled H4.V peaks served as control.
(D) ChIP enrichment profiles at H4.V peaks from H4.V ChIP-seq and H4 ChIP-seq.

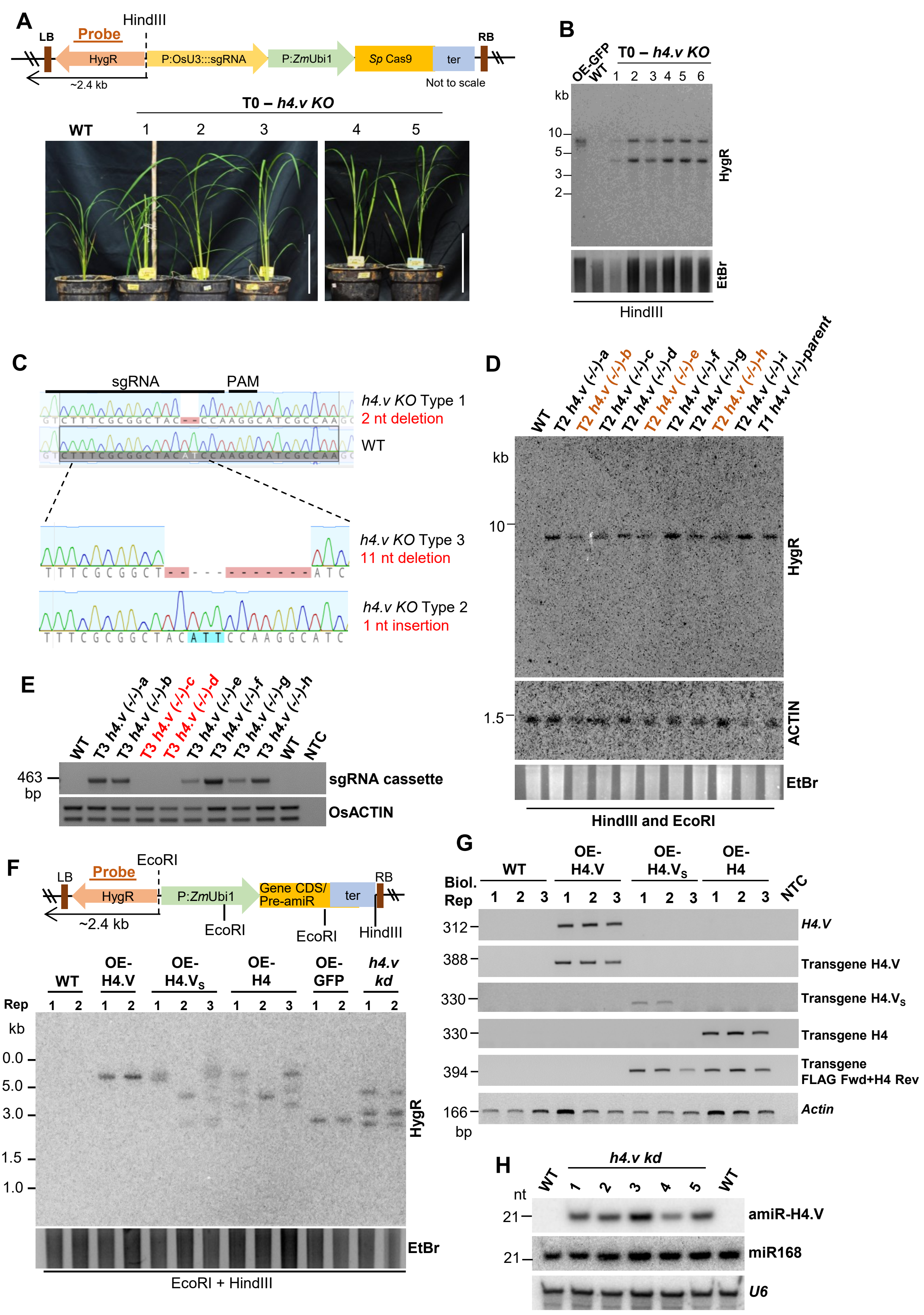
A
LB
Probe
HindIII
HygR
P:OsU3:::sgRNA
P:ZmUbi1
Sp Cas9
ter
RB
~2.4 kb
Not to scale
T0 – h4.v KO
WT
1
2
3
4
5
B
OE-GFP
WT
T0 – h4.v KO
1 2 3 4 5 6
kb
10
5
3
2
HygR
EtBr
HindIII
C
sgRNA
PAM
h4.v KO Type 1
2 nt deletion
WT
h4.v KO Type 3
11 nt deletion
h4.v KO Type 2
1 nt insertion
D
WT
T2 h4.v (-/-)-a
T2 h4.v (-/-)-b
T2 h4.v (-/-)-c
T2 h4.v (-/-)-d
T2 h4.v (-/-)-e
T2 h4.v (-/-)-f
T2 h4.v (-/-)-g
T2 h4.v (-/-)-h
T2 h4.v (-/-)-i
T1 h4.v (-/-)-parent
kb
10
1.5
HygR
ACTIN
EtBr
HindIII and EcoRI
E
WT
T3 h4.v (-/-)-a
T3 h4.v (-/-)-b
T3 h4.v (-/-)-c
T3 h4.v (-/-)-d
T3 h4.v (-/-)-e
T3 h4.v (-/-)-f
T3 h4.v (-/-)-g
T3 h4.v (-/-)-h
WT
NTC
463 bp
sgRNA cassette
OsACTIN
F
LB
Probe
EcoRI
HygR
P:ZmUbi1
Gene CDS/ Pre-amiR
ter
RB
~2.4 kb
EcoRI
EcoRI
HindIII
WT
OE-H4.V
OE-H4.Vs
OE-H4
OE-GFP
h4.v kd
Rep
1 2 1 2 1 2 3 1 2 3 1 2 1 2
kb
10.0
5.0
3.0
1.5
1.0
HygR
EtBr
EcoRI + HindIII
G
Biol. Rep
WT
OE-H4.V
OE-H4.Vs
OE-H4
1 2 3 1 2 3 1 2 3 1 2 3
NTC
312
388
330
330
394
166
bp
H4.V
Transgene H4.V
Transgene H4.Vs
Transgene H4
Transgene FLAG Fwd+H4 Rev
Actin
H
WT
h4.v kd
1 2 3 4 5
WT
nt
21
21
amiR-H4.V
miR168
U6

Extended Data Fig. 4. Generation of *h4.v KO* and H4.V perturbed lines in rice.
(A) T-DNA map of the Cas9 construct used for generating the *h4.v KO*. T0 *h4.v KO* images of 5-weeks old plants are shown. Scale: 10 inches.
(B) Southern blots showing the junction fragment profiles of T0 *h4.v KO* lines. OE-GFP served as positive control.
(C) Mutation profiles of T1 *h4.v KO* plants compared to WT. Three types of mutations were obtained and the type 1 mutation was taken for the analysis.
(D) Junction fragment Southern analysis of the homozygous (-/-) T1 *h4.v KO* parent (that segregated as single copy transgene) and its T2 progeny. Plants marked in brown are putative parents that are heterozygous for the transgene.
(E) Genomic DNA PCR showing the T3 segregants of the T2 parents (marked brown in (D)). Actin was used a loading control and sgRNA region as transgene marker. (A, B and D) Probe (HygR) used for T-DNA junction fragment Southern blots is marked (brown). EtBr-stained gel served as loading control.
(F) T-DNA map and the junction fragment (arrow) Southern analyses of transgenic plants overexpressing H4.V, H4.V_S and H4 or GFP (3x-FLAG tagged at the N-terminal) or the precursor of amiR. HygR was used as probe (brown bar). EtBr-stained gel is the loading control.
(G) RT-PCR (semi-quantitative) gels showing the OE of the transgenes in leaves using specific primers. NTC-no template control. Actin was used as loading control.
(H) sRNA northern blots showing the accumulation of the amiR targeting H4.V in *h4.v kd* plants. *U6* served as control.

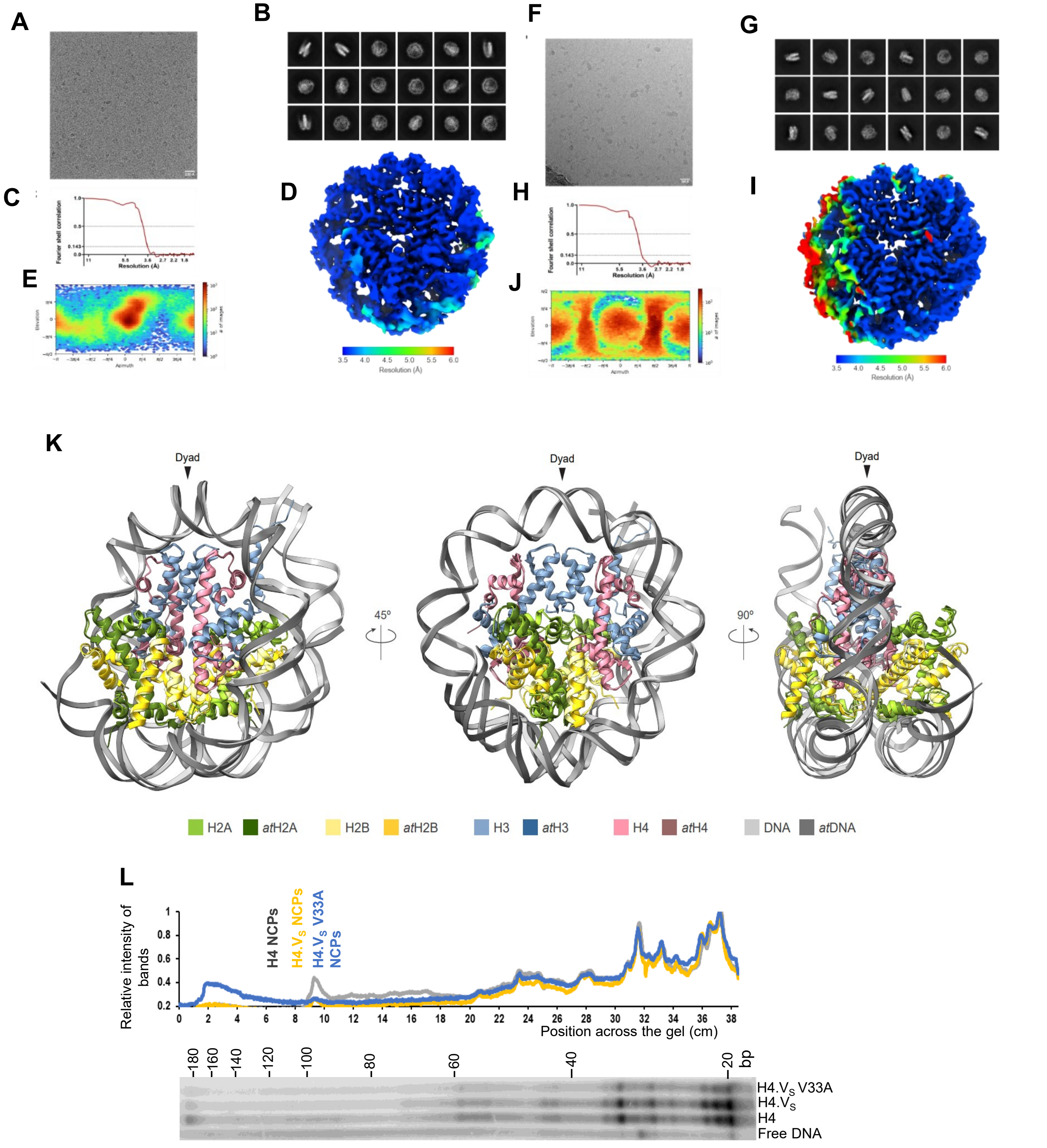

Extended Data Fig. 5. Cryo-EM data processing of NCPs and DNA interactions in rice NCPs.
(A-J) Data collection and refinement for rice canonical NCP (A-E) and H4.Vs NCP (F-J). Representative cryo-EM micrographs (A, F), representative 2D classes (B, G), Fourier Shell Correlation curves (C, H) showing the resolution estimation of the maps, local resolution maps (D, J) and angular distribution of the particles (E,J) for the NCPs were depicted.
(K) Overlay images of *Arabidopsis* and rice (H4) NCP depicting that the atypical DNA accessibility in plant nucleosomes is due to variations in the H2B. Magnified images depict the residue necessary for the variable DNA wrapping.
(L) DNase foot-printing assay of the 188 bp NCPs with the corresponding band intensity profile showing no major distinction in the way DNA is protected by the octamers within the core of the NCPs..

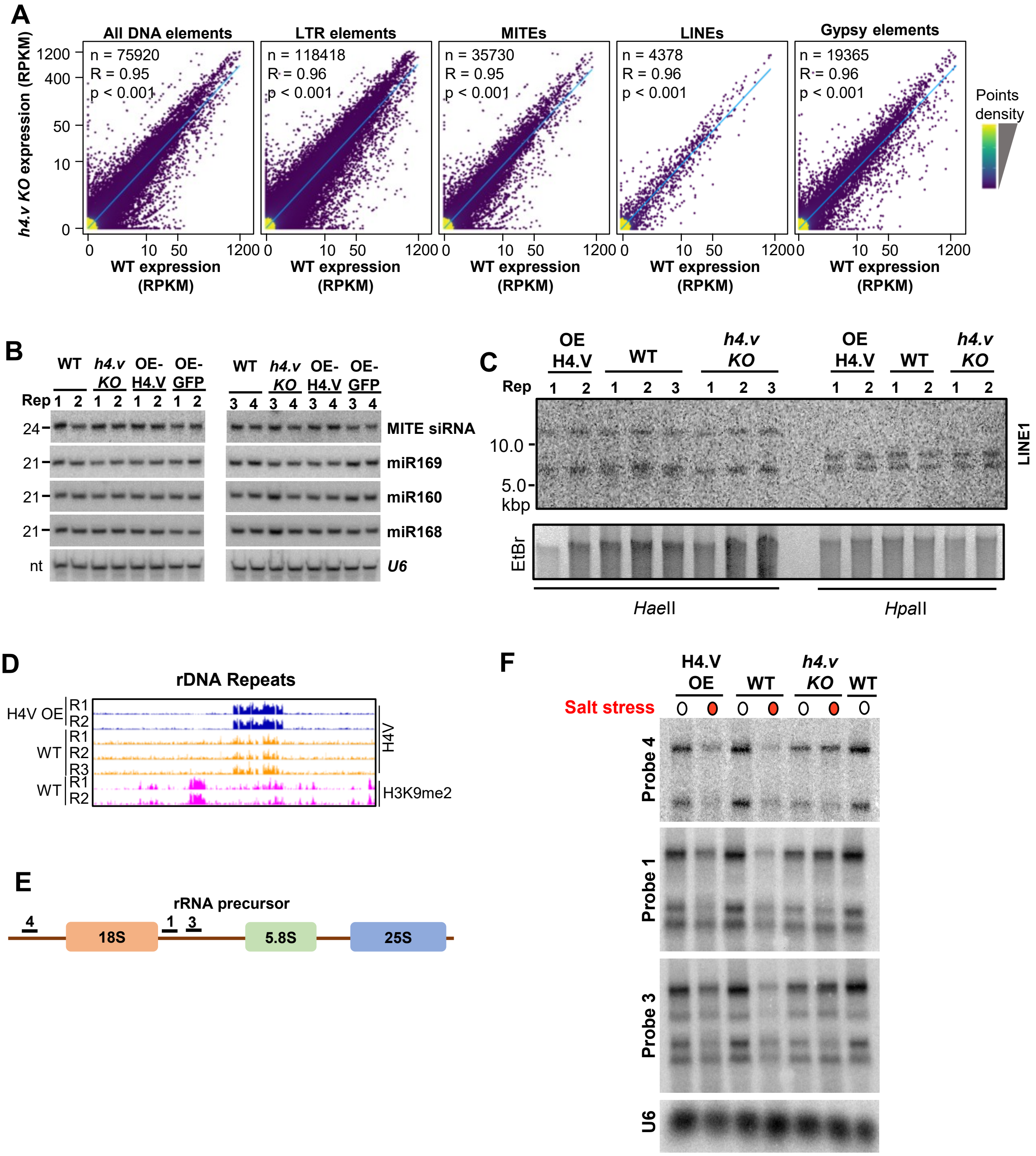

Extended Data Fig. 6. H4.V perturbation does not affect the silencing of transposons but mis-regulate rDNA expression.

(A) Density scatter plots showing expression difference of different categories of transposons. Pearson correlation coefficient (R) and *p*-values are mentioned. Points density are mentioned with colour gradient.

(B) sRNA northern blots showing abundance of repeat-derived sRNAs or other miRNAs. *U6* was the loading control.

(C) Methylation sensitive Southern blot hybridised with LINE1 probe. EtBr-stained gel served as loading control.

(D) IGV screenshots showing occupancy of H4.V at rDNA arrays in chromosome 1.

(E - F) RNA blots showing the expression of rRNA precursor regions (precursor structure and probe regions marked in (E)). *U6* served as loading control.

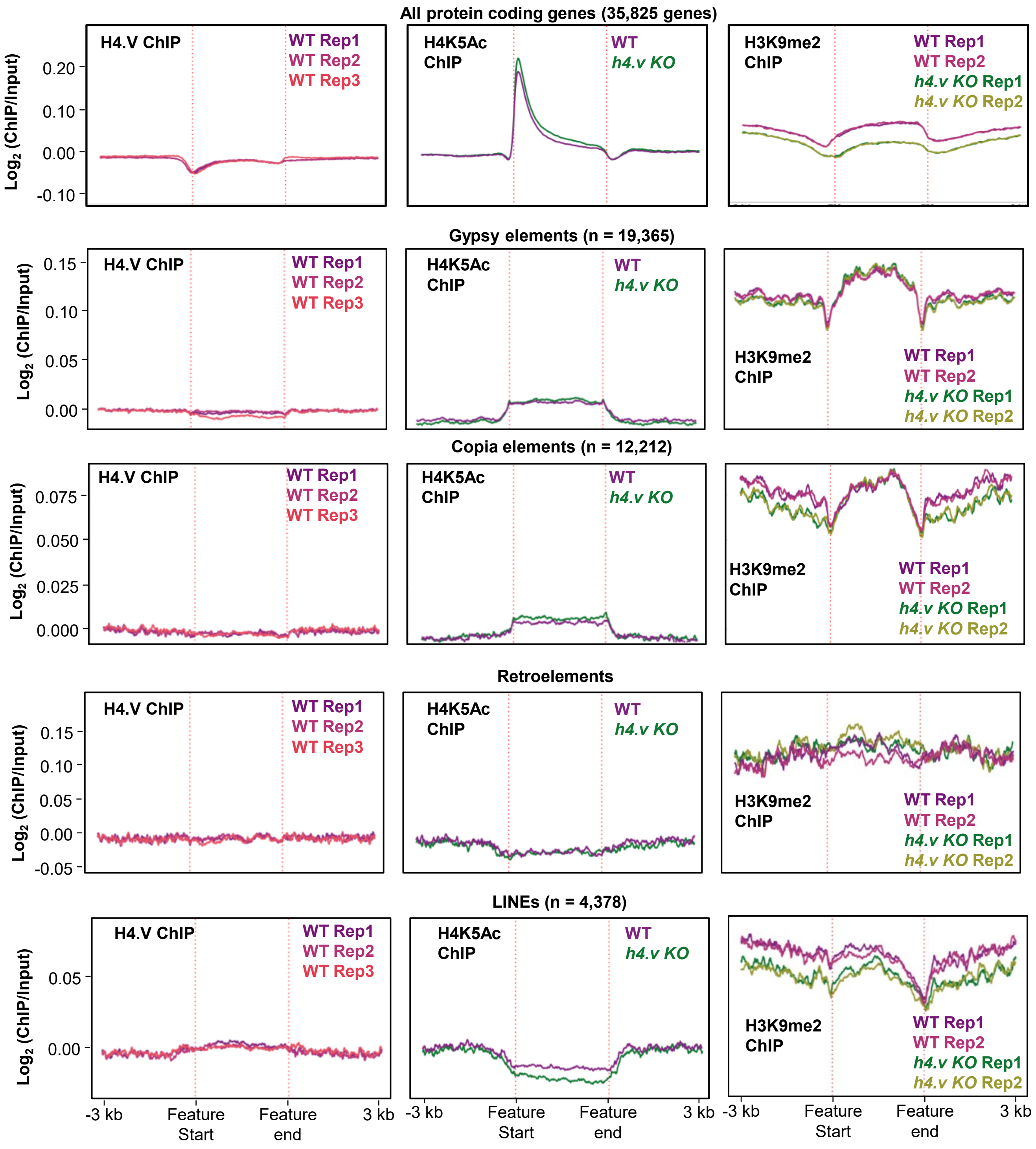

Extended Data Fig. 7. Occupancy of H4.V over protein coding genes and repeats
Metaplots showing the occupancy of H4.V, H4K5Ac and H3K9me2 marks over the protein coding genes and other annotated transposons and repeats.

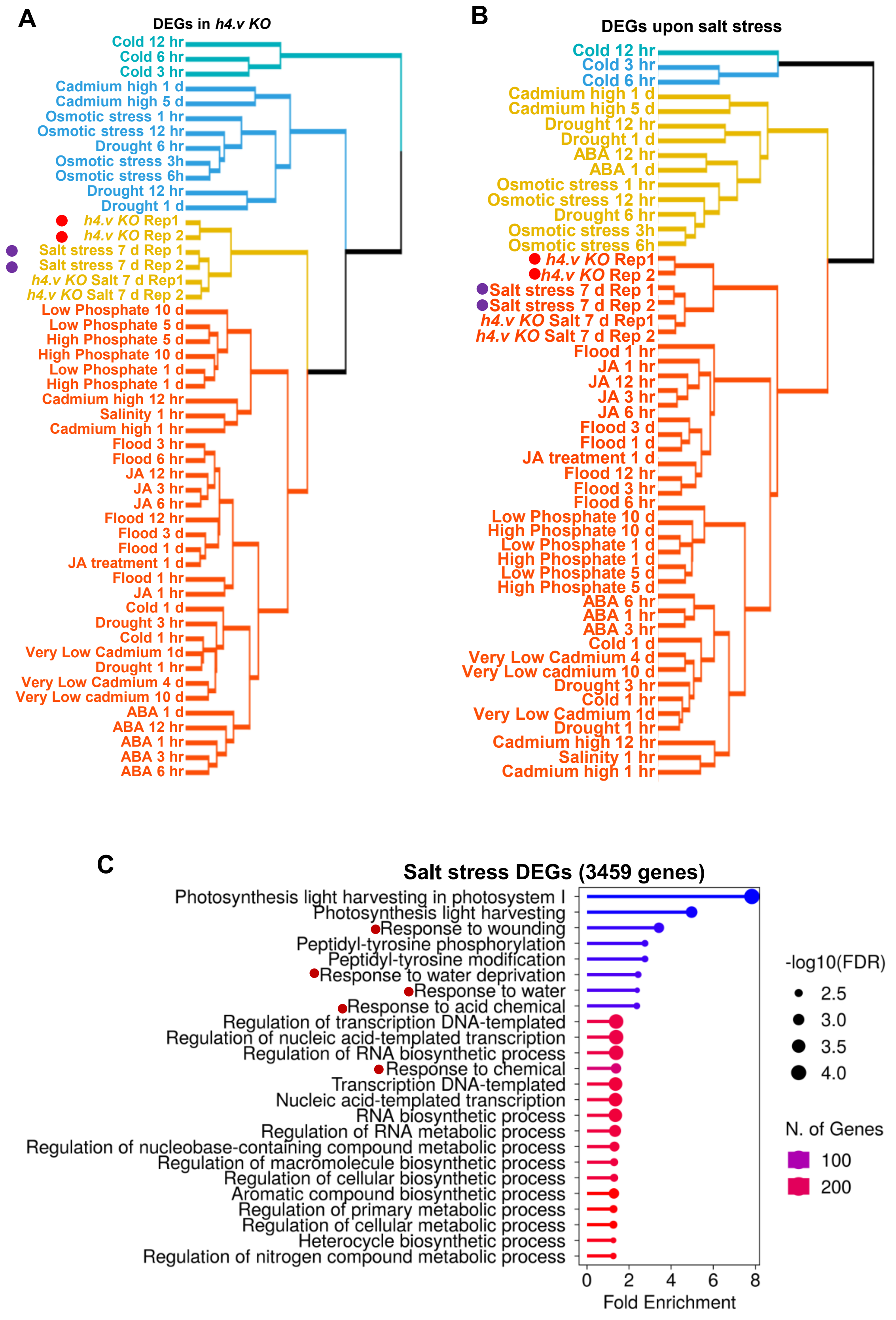

Extended Data Fig. 8. KO of H4.V exhibits a transcriptome similar to salt stressed state.
(A-B) Clustered dendrograms comparing transcriptomes across different stress conditions from TENOR datasets, analyzed for the *h4.v KO* DEGs (A), or the salt stress DEGs (B). Red dots highlight the *h4.v KO* profiles and purple dots represent salt stress profiles. Figures 5C and D depict representative examples and replicates are shown here. The tree is clustered into four hierarchical types.
(C) Gene Ontology enrichment analysis of salt stress DEGs. Red dots represent categories associated with salt stress responses.

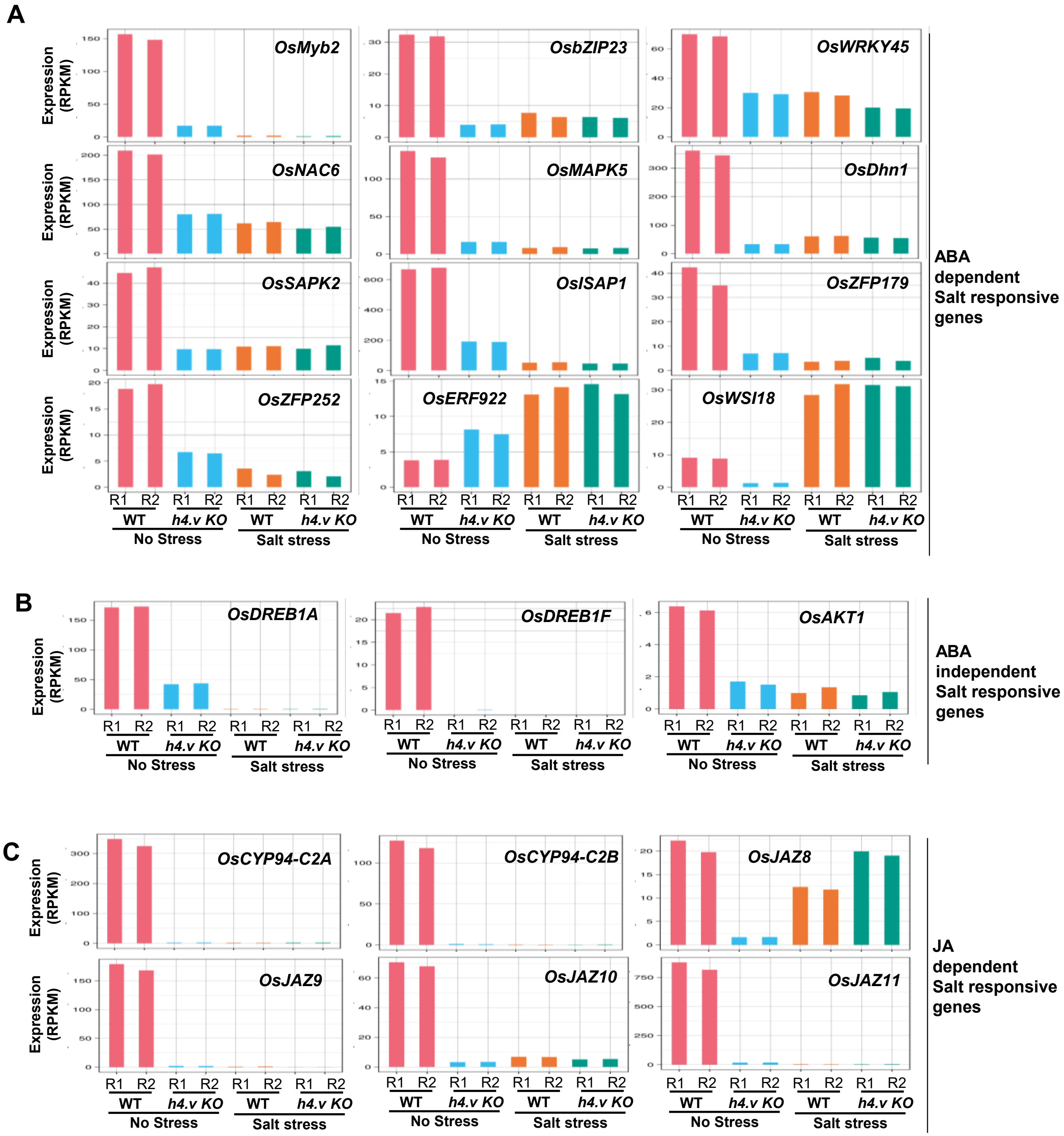

Extended Data Fig. 9. Knockout of H4.V results in misregulation of phytohormonal pathway genes responsive to salt stress.
(A-C) Gene expression levels in WT and *h4.v KO* with and without salt stress. Gene names are mentioned.

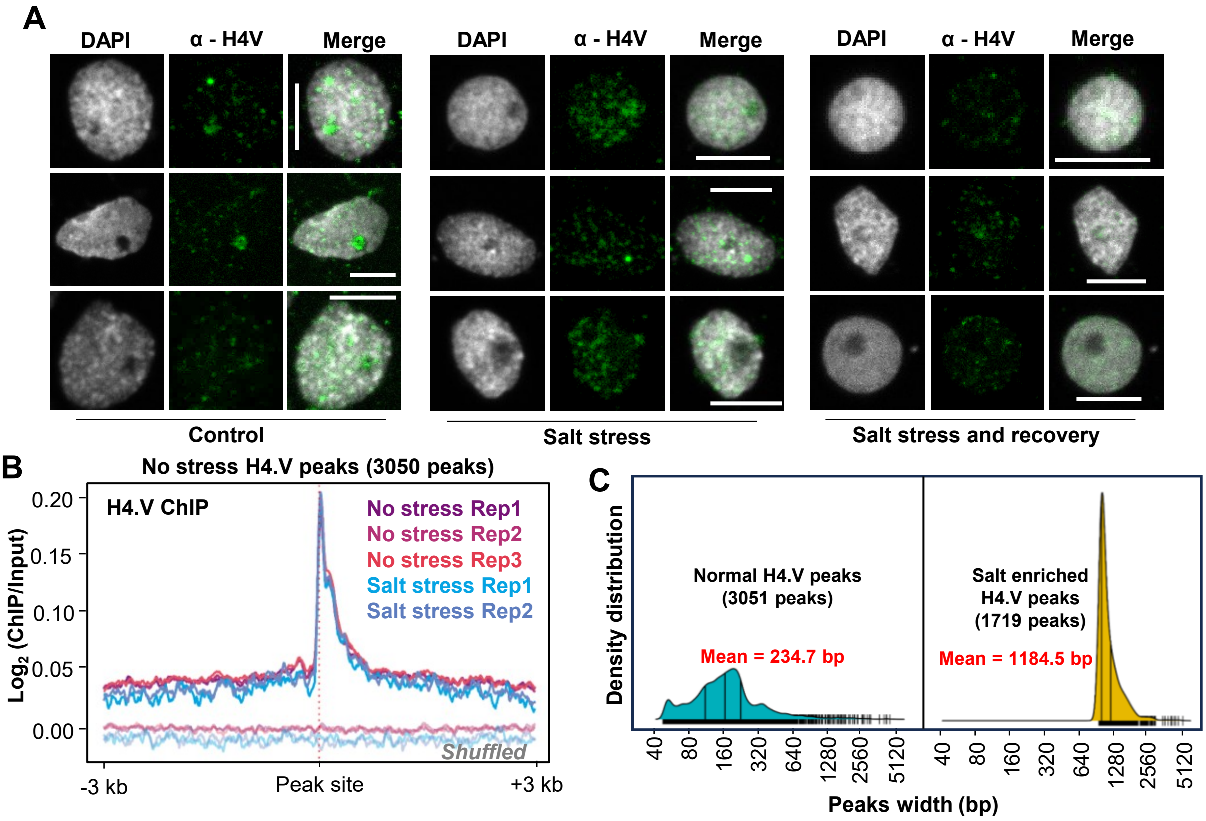

Extended Data Fig. 10. Salt-stress dependent variations in H4.V.

(A) Immunostaining of H4.V showing the persistent occupancy of the H4.V upon salt stress followed by recovery. Salt stress is administered for 14 days followed by recovery. Scale: 5 µm.

(B) Metaplots comparing the occupancy of H4.V with or without stress over the peaks identified without salt stress (3050 peaks). Enrichment over the shuffled set of peaks are shown in grey shade.

(C) Density distribution plots of the H4.V peak widths. Mean peak width is shown in red and quartiles of size distribution is marked in black.

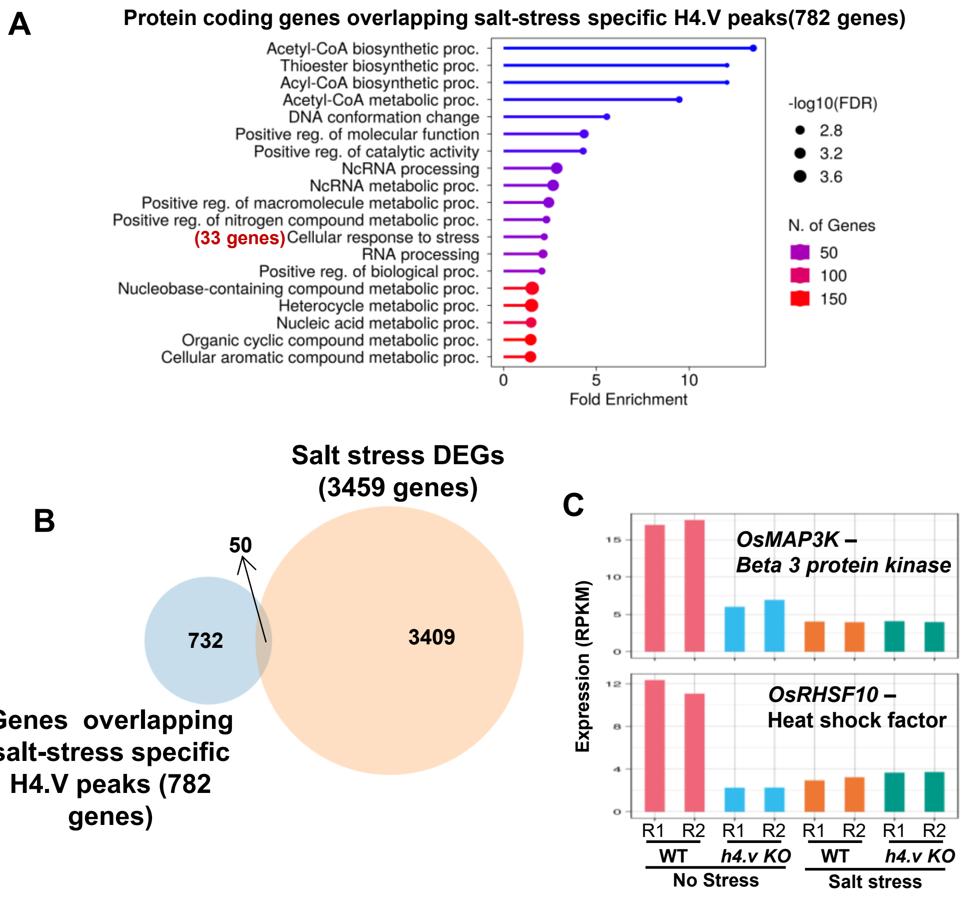

Extended Data Fig. 11. Salt-stress specific H4.V peaks occupy protein coding genes.
(A) Gene Ontology enrichment analysis of salt stress DEGs.
(B) Venn diagram representing the overlap of salt stress DEGs and the genes that are occupied by H4.V upon salt stress.
(C) Gene expression levels in WT and *h4.v KO* with and without salt stress. Gene names are mentioned. These are representative genes that are master-regulators of stress responses that are occupied by H4.V upon salt stress.

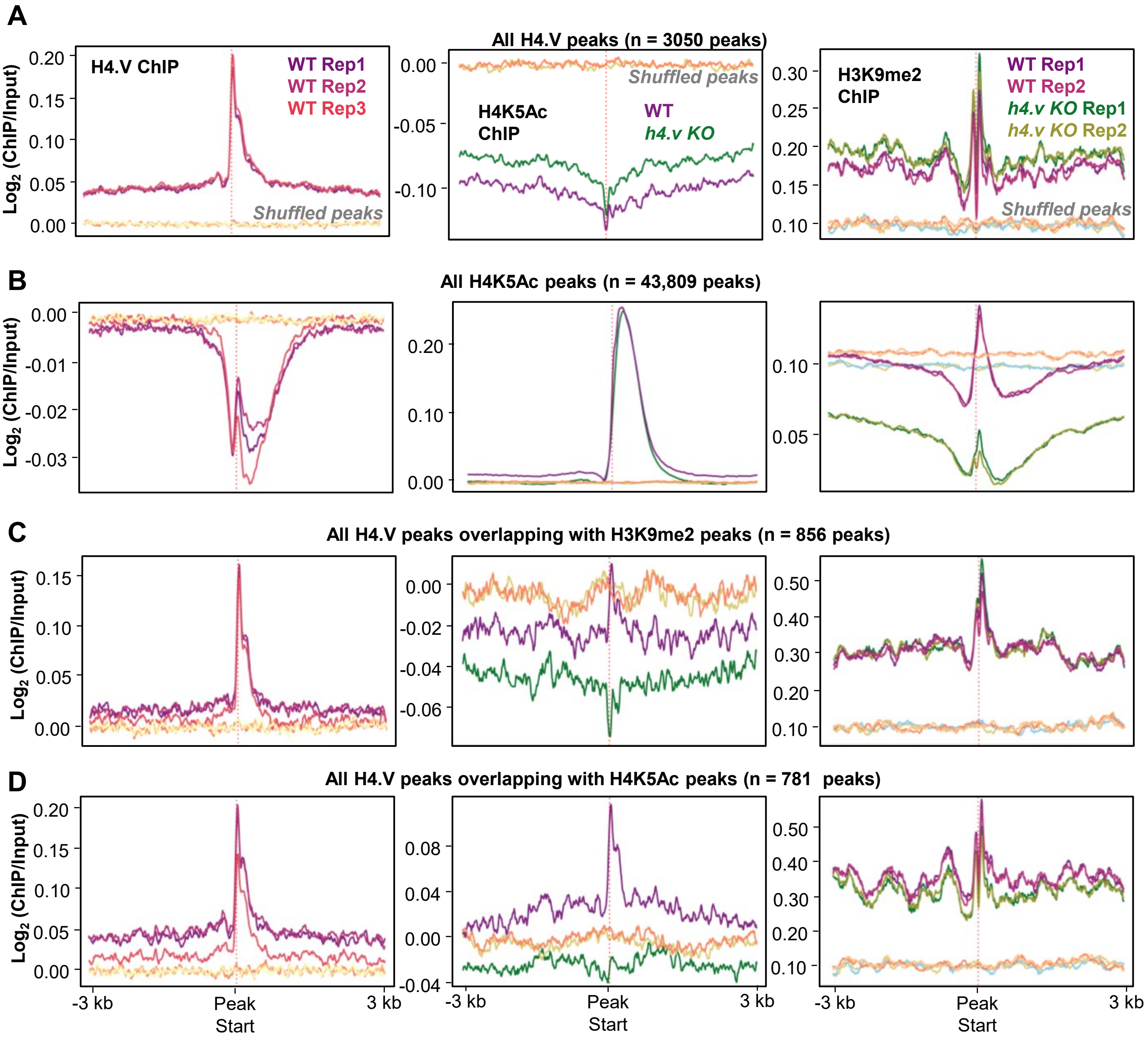

Extended Data Fig. 12. Co-occupancy and regulation of H4.V and other histone marks
(A-D) Metaplots showing the occupancy of histone marks (H4.V, H4K5Ac and H3K9me2) over all H4.V peaks (A), all H4K5Ac peaks (B), H4.V peaks overlapping with H3K9me2 peaks (C) and H4.V peaks overlapping with H4K5Ac peaks (D). Enrichment over the shuffled set of peaks are shown as control for background enrichment.

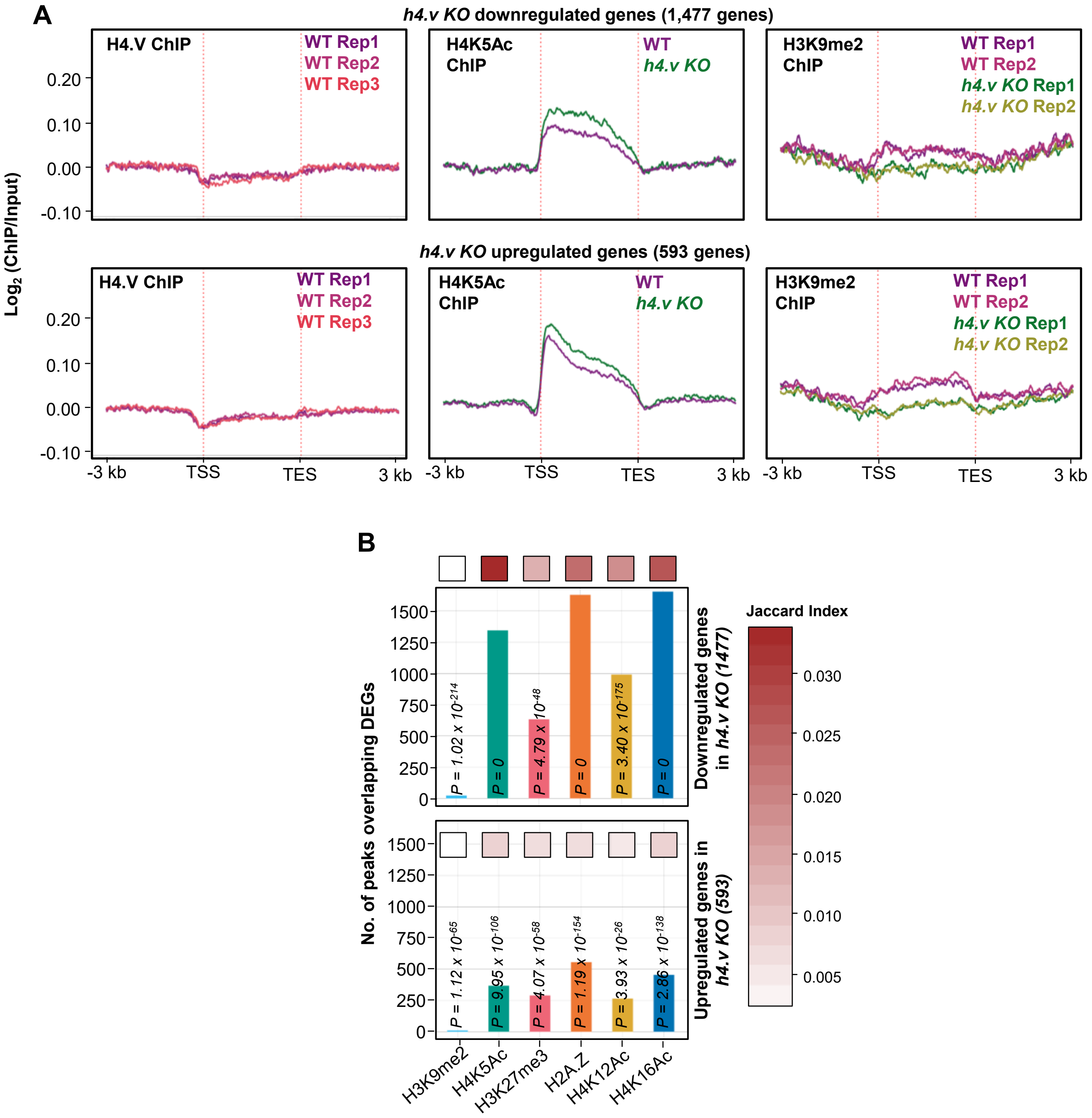

Extended Data Fig. 13. ***h4.v KO*** **DEGs are not directly occupied by H4.V.**
(A) Metaplots showing the occupancy of H4.V, H4K5Ac and H3K9me2 marks over the *h4.v KO* DEGs.
(B) Bar plots showing number of overlaps of *h4.v KO* DEGs with peak sets of other histone marks. Significance of overlap was tested using hyper-geometric test and p-values are mentioned. Jaccard index (shown as heatmap) represents the strength of overlap.

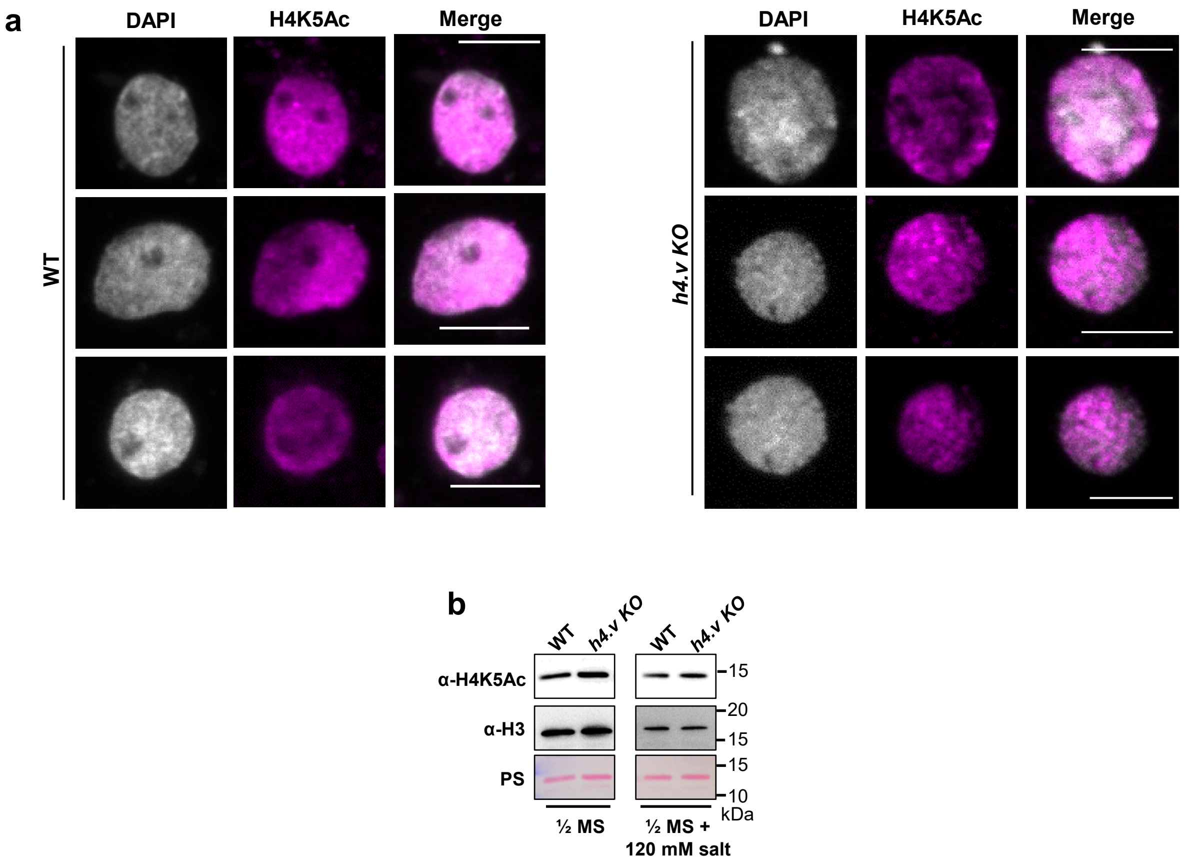

Extended Data Fig. 14. Perturbation of H4 variant does not lead to global changes in H4K5Ac levels.
(a) IFL images showing occupancy of H4K5Ac marks in WT and *h4.v KO* seedlings nuclei. Scale: 5 µm.
(b) Immunoblots showing the global levels of H4K5Ac marks without and with salt stress. H3 levels and Ponceau stained blots served as loading controls.
