# Supplementary material for: Oryza genera-specific novel Histone H4 variant predisposes H4 Lysine5 Acetylation marks to modulate salt stress responses
Source: Nat Plants. Author manuscript; Available in PMC 2025 May 14. (PMC7617672; doi:10.1038/s41477-025-01974-2)
Supplement: Supplementary Tables [file EMS204895-supplement-Supplementary_Tables.pdf]

Supplementary Table 1. Histone H4 sequences from different species.

>XP_015610672.1 histone H4 [Oryza sativa Japonica Group
MSGRGKGGKGLGKGGAKRHRKVLRDNIQGITKPAIRRLARRGGVKRISGLIYEETRGVLKIFLENVIR
DAVTYTEHARRKTVTAMDVVYALKRQGRTLYGFGG
>Os_H4_variant
MAPRSVAISGRGTSGARRHRIVFRGYIQGIAKPVIRRLARKGGVKRISGLIYKETRGVLEIFLKNVIR
DAITYTEHAHRKTVMAMDVVYALKLQGRTIYDFGG
>NP_001027284.1 histone H4 [Drosophila melanogaster]
MTGRGKGGKGLGKGGAKRHRKVLRDNIQGITKPAIRRLARRGGVKRISGLIYEETRGVLKVFLENVIR
DAVTYTEHAKRKTVTAMDVVYALKRQGRTLYGFGG
>NP_001029249.1 histone H4 [Homo sapiens]
MSGRGKGGKGLGKGGAKRHRKVLRDNIQGITKPAIRRLARRGGVKRISGLIYEETRGVLKVFLENVIR
DAVTYTEHAKRKTVTAMDVVYALKRQGRTLYGFGG
>ACG31227.1 histone H4 [Zea mays]
MSGRGKCGKGLGKGGAKRHRKVLRDNIQGITKPAIRRLARRGGVKRISGLIYEETRGVLKIFLENVIR
DAVTYTEHARRKTVTAMDVVYALKRQGRTLYGFGG
>AAI11814.1 Hist4h4 protein, partial [Mus musculus]
MSGRGKGGKGLGKGGAKRHRKVLRDNIQGITKPAIRRLARRGGVKRISGLIYEETRGVLKVFLENVIR
DAVTYTEHAKRKTVTAMDVVYALKRQGRTLYGFGG
>NP_009563.1 histone H4 [Saccharomyces cerevisiae S288C]
MSGRGKGGKGLGKGGAKRHRKILRDNIQGITKPAIRRLARRGGVKRISGLIYEEVRAVLKSFLESVIR
DSVTYTEHAKRKTVTSLDVVYALKRQGRTLYGFGG
>NP_001332089.1 Histone superfamily protein [Arabidopsis thaliana]
MSGRGKGGKGLGKGGAKRHRKVLRDNIQGITKPAIRRLARRGGVKRISGLIYEETRGVLKIFLENVIR
DAVTYTEHARRKTVTAMDVVYALKRQGRTLYGFGG
>AFK45965.1 unknown [Medicago truncatula]
MSGRGKGGKGLGKGGAKRHRKVLRDNIQGITKPAIRRLARRGGVKRISGLIYEETRGVLKVFLENVIR
DAVTYTEHARRKTVTAMDVVYALKRQGRTLYGFGG
>XP_024359910.1 histone H4 [Physcomitrium patens]
MSGRGKGGKGLGKGGAKRHRKVLRDNIQGITKPAIRRLARRGGVKRISGLIYEETRSVLKIFLENVIR
DAVTYTEHARRKTVTAMDVVYALKRQGRTLYGFGG
>atr:18424547 K11254 histone H4 | (RefSeq) histone H4 (A) [Amborella trichopoda]
MSGRGKGGKGLGKGGAKRHRKVLRDNIQGITKPAIRRLARRGGVKRISGLIYEETRGVLK
IFLENVIRDAVTYTEHARRKTVTAMDVVYALKRQGRTLYGFGG
>PTQ27186.1 hypothetical protein MARPO_0214s0009 [Marchantia polymorpha]
MSGRGKGGKGLGKGGAKRHRKVFRDNIQGITKPAIRRLARRGGVKRISGLIYEETRGVLKIFLENVIR
DAVTYTEHARRKTVTAMDVVYALKRQGRTLYGFGG
>NP_003538.1 histone H4-like protein type G [Homo sapiens]
MSVRGKAGKGLGKGGAKCHRKVLSDNIQGITKCTIRRLARHGGVKRILGLIYEETRRVFKVFLENVIW
YAVTNTEHAKRKTVTAMAVVYVLKRQGRTL
>XP_044458683.1 histone H4-like [Triticum aestivum]
MSGRGKGGKGLGKGGTKRHRKVLRDNIQGITKPAIRRLARRGGVKRISGLIYEETRGVLKIFLENVIR
DAVTYTEHARRKTVTAMDVVYALKRQGRTLYGFGG
>XP_003557745.1 histone H4 [Brachypodium distachyon]
MSGRGKGGKGLGKGGAKRHRKVLRDNIQGITKPAIRRLARRGGVKRISGLIYEETRGVLKIFLENVIR
DAVTYTEHARRKTVTAMDVVYALKRQGRTLYGFGG
>XP_009103711.2 histone H4 [Brassica rapa]
MSGRGKGGKGLGKGGAKRHRKVLRDNIQGITKPAIRRLARRGGVKRISGLIYEETRGVLKIFLENVIR
DAVTYTEHARRKTVTAMDVVYALKRQGRTLYGFGG

Supplementary Table 2. Details of high-throughput genomics data generated in this study

Sl. No.	Dataset type	Genotype	Replicate	Source tissue	GSM number	GSE number	Sequencing mode
1	RNA-seq	WT	Rep1	Seedlings without stress. 14 days old.	GSM7166152	GSE229601	Paired end-100bp
2	RNA-seq	WT	Rep2		GSM7166153	GSE229601	Paired end-100bp
3	RNA-seq	*h4.v KO*	Rep1		GSM7166148	GSE229601	Paired end-100bp
4	RNA-seq	*h4.v KO*	Rep2		GSM7166149	GSE229601	Paired end-100bp
5	RNA-seq	WT	Rep1	Seedlings with 120 mM salt stress. 14 days old.	GSM7166154	GSE229601	Paired end-100bp
6	RNA-seq	WT	Rep2		GSM7166155	GSE229601	Paired end-100bp
7	RNA-seq	*h4.v KO*	Rep1		GSM7166150	GSE229601	Paired end-100bp
8	RNA-seq	*h4.v KO*	Rep2		GSM7166151	GSE229601	Paired end-100bp
9	RNA-seq	WT	Rep1	Mature Leaves from 60 days old plants.	GSM7166156	GSE229601	Paired end-100bp
10	RNA-seq	WT	Rep2		GSM7166157	GSE229601	Paired end-100bp
11	RNA-seq	OE-H4.V	Rep1		GSM7166158	GSE229601	Paired end-100bp
12	RNA-seq	OE-H4.V	Rep2		GSM7166159	GSE229601	Paired end-100bp
13	RNA-seq	OE-H4.V$_S$	Rep1		GSM7166162	GSE229601	Paired end-100bp
14	RNA-seq	OE-H4.V$_S$	Rep2		GSM7166163	GSE229601	Paired end-100bp
15	RNA-seq	OE-H4	Rep1		GSM7166160	GSE229601	Paired end-100bp
16	RNA-seq	OE-H4	Rep2		GSM7166161	GSE229601	Paired end-100bp

17	ChIP-seq with α-H4.V	WT	Rep1	Seedlings without stress. 14 days old.	GSM7166118	GSE229599	Single end-50bp
18	ChIP-seq with α-H4.V	WT	Rep2		GSM7166119	GSE229599	Single end-50bp
19	ChIP-seq with α-H4.V	*WT*	Rep3		GSM7166120	GSE229599	Single end-50bp
20	ChIP-seq with α-H4.V	*h4.v KO*	Rep1		GSM7166107	GSE229599	Single end-50bp
21	ChIP-seq with α-H4.V	*h4.v KO*	Rep2		GSM7166107	GSE229599	Single end-50bp
22	ChIP-seq with α-H4.V	WT	Rep1	Seedlings with 120 mM salt stress. 14 days old.	GSM7166121	GSE229599	Single end-50bp
23	ChIP-seq with α-H4.V	WT	Rep2		GSM7166122	GSE229599	Single end-50bp
24	ChIP-seq with α-H4K5Ac	WT	Rep1	Seedlings without stress. 14 days old.	GSM7166114	GSE229599	Single end-50bp
25	ChIP-seq with α-H4K5Ac	WT	Rep2		GSM7166115	GSE229599	Single end-50bp
26	ChIP-seq with α-H4K5Ac	*h4.v KO*	Rep1		GSM7166103	GSE229599	Single end-50bp
27	ChIP-seq with α-H4K5Ac	*h4.v KO*	Rep2		GSM7166104	GSE229599	Single end-50bp
28	ChIP-seq with α-H4K5Ac	WT	Rep1	Seedlings with 120 mM salt stress. 14 days old.	GSM7166116	GSE229599	Single end-50bp
29	ChIP-seq with α-H4K5Ac	WT	Rep2		GSM7166117	GSE229599	Single end-50bp
30	ChIP-seq with α-H4K5Ac	*h4.v KO*	Rep1		GSM7166105	GSE229599	Single end-50bp
31	ChIP-seq with α-H4K5Ac	*h4.v KO*	Rep2		GSM7166106	GSE229599	Single end-50bp
32	ChIP-seq with α-H4K5Ac	OE-H4.V	Rep1	Seedlings without stress. 14 days	GSM7166109	GSE229599	Single end-50bp
33	ChIP-seq with α-H4K5Ac	OE-H4.V	Rep2		GSM7166110	GSE229599	Single end-50bp

34	ChIP-seq with α-H4.V	OE-H4.V	Rep1	old.	GSM7166111	GSE229599	
35	ChIP-seq with α-H4.V	OE-H4.V	Rep2		GSM7166112	GSE229599	
36	ChIP-seq with α-H4C	WT	Rep1		GSM7166113	GSE229599	Single end-50bp
37	ChIP-seq with α-H4C	*h4.v KO*	Rep1		GSM7166102	GSE229599	Single end-50bp
38	MNase sensitivity on NCPs	Undigested Input DNA	Rep1	Synthetic NCPs	GSM7166165	GSE229602	Paired end-100bp
39	MNase sensitivity on NCPs	H4 NCPs	Rep1	Synthetic NCPs	GSM7166164	GSE229602	Paired end-100bp
40	MNase sensitivity on NCPs	H4.V NCPs	Rep1	Synthetic NCPs	GSM7166166	GSE229602	Paired end-100bp

Supplementary Table 3. Details of high-throughput genomics data obtained from publicly available datasets

Sl. No.	Dataset type	Genotype	Source tissue	SRA number	GSE number	Reference
1	ChIP-seq with α-H3K9me2 Rep1	WT	seedlings	SRX7426641	GSE142462	(Zhao *et al*, 2020)
2	ChIP-seq with α-H3K9me2 Rep2	WT	seedlings	SRX7426642	GSE142462	(Zhao *et al*, 2020)
3	ChIP-seq with α-H2A.Z Rep1	WT	Seedlings	SRX2860628	GSE155269	(Du *et al*, 2020)
4	ChIP-seq with α-H2A.Z Rep2	WT	Seedlings	SRX2860589	GSE155269	(Du *et al*, 2020)
5	ChIP-seq with α-H2A.Z Rep3	WT	Seedlings	SRX1883882	GSE155269	(Du *et al*, 2020)
6	ChIP-seq with α-H3K27me3 Rep1	WT	Seedlings	SRX1131812	GSE71640	(Zhou *et al*, 2016)
7	ChIP-seq with α-H3K27me3 Rep2	WT	Seedlings	SRX2004187	GSE71640	(Zhou *et al*, 2016)
8	ChIP-seq with α-H4K12Ac Rep1	WT	Seedlings	SRX038893	GSE26734	(Zhang *et al*, 2012)
9	ChIP-seq with α-H4K16Ac Rep1	WT	Seedlings	SRX1044780	GSE69426	(Lu *et al*, 2015)
10	Input_ChIP-seq_Rep1	WT	Seedlings	SRX10085735	GSE166665	(Pachamuthu *et al*, 2022)
11	Input_ChIP-seq_Rep2	WT	Seedlings	SRX10085736	GSE166665	(Pachamuthu *et al*, 2022)

References

Du K, Luo Q, Yin L, Wu J, Liu Y, Gan J, Dong A & Shen W-H (2020) OsChz1 acts as a histone chaperone in modulating chromatin organization and genome function in rice. *Nat Commun* 11: 5717

Lu L, Chen X, Sanders D, Qian S & Zhong X (2015) High-resolution mapping of H4K16 and H3K23 acetylation reveals conserved and unique distribution patterns in Arabidopsis and rice. *Epigenetics* 10: 1044–1053

Pachamuthu K, Hari Sundar G V, Narjala A, Singh RR, Das S, Avik Pal HCY & Shivaprasad PV (2022) Nitrate-dependent regulation of miR444-OsMADS27 signalling cascade controls root development in rice. *J Exp Bot* 73: 3511–3530

Zhang W, Wu Y, Schnable JC, Zeng Z, Freeling M, Crawford GE & Jiang J (2012) High-resolution mapping of open chromatin in the rice genome. *Genome Res* 22: 151–162

Zhao L, Xie L, Zhang Q, Ouyang W, Deng L, Guan P, Ma M, Li Y, Zhang Y, Xiao Q, *et al* (2020) Integrative analysis of reference epigenomes in 20 rice varieties. *Nat Commun* 11: 2658

Zhou S, Liu X, Zhou C, Zhou Q, Zhao Y, Li G & Zhou D-X (2016) Cooperation between the H3K27me3 chromatin Mark and non-CG methylation in epigenetic regulation. *Plant Physiol* 172: 1131–1141

Supplementary Table 4. List of oligos and probes used in this study

Oligo Name	Oligo ID	Oligo sequence (5' – 3')	Purpose	Reference
*h4.v-kd*_miR-s I	-	agtagacaatccgatcgtgcctacaggagattcagtttga	amiR:*h4.v-kd* construct	This study
*h4.v-kd*_miR-a II	-	tgtaggcacgatcggattgtctactgctgctgctacagcc	amiR:*h4.v-kd* construct	This study
*h4.v-kd*_miR*s III	-	cttaggctcgaacggattgtctattcctgctgctaggctg	amiR:*h4.v-kd* construct	This study
*h4.v-kd*_miR*a IV	-	aatagacaatccgttcgagcctaagagaggcaaaagtgaa	amiR:*h4.v-kd* construct	This study
OsH4.V_CDS_F	1360	atggcgcctcgttcggtagc	RT-qPCR and RT-PCR	This study
OsH4.V_CDS_R	1361	tcaaccgccgaagtcatagatggtg	RT-qPCR and RT-PCR	This study
Widom601_146bp_amp_F	4712	acaggatgtatatatgtgacacg	Widom 601 146bp NPS amplification	1
Widom601_146bp_amp_R	4713	ctggagaatcccggtgcc	Widom 601 146bp NPS amplification	1
Widom601_188bp_amp_F	4685	tatgaatttcgcgacacaaggcctggatgtatatatgtgacacgtgcc	Widom 601 188bp NPS amplification	This study
Widom601_188bp_amp_R	4686	ggaccctatcgcgagccaggcctgagaatcccggtgccgaggccgctcaat	Widom 601 188bp NPS amplification	This study
H4.V_Prom_F	1497	ggactgcagccgacagtggtgcatggtttttccaatttggagtttg	3xFLAG-H4.V-GFP construct	This study
H4.V_Prom_R	1498	ctcgtcgacatcatcggctactgcgccgacggcgaccagcggctc	3xFLAG-H4.V-GFP construct	This study
Transgene_Flag_F	1463	ggactataaggaccacgacggag	OE-transgene validation	This study
Transgene H4 and H4.V reverse	3358	ttacccgggctagcctccgaagccgtacag	OE-H4-H4.V transgene validation	This study
Transgene H4 Fwd	3359	ggaactagtatgtccggcagaggaaaggg	OE-H4 transgene validation	This study
Transgene H4.V_S Fwd	3357	ttaactagtatggcaccgcgtagcgttgc	OE-H4.V_S transgene validation	This study
HygR_F	415	aaagcctgaactcaccgc	Southern probe	This study
HygR_R	416	ggtttccactatcggcga	Southern probe	This study
OsGAPDH_F	3873	gggtattctgggttacgttgaggag	RT-qPCR	This study
OsGAPDH_R	3874	acggatcaggtcaacaacgcgagag	RT-qPCR	This study
OsActin1_F	1786	gctatgtacgtcgccatccagg	RT-qPCR and RT-PCR	This study
OsActin1_R	1787	tgagatcacgcccagcaagg	RT-qPCR and RT-PCR	This study

Actin_F	2049	tccatcttggcatctctcag	Southern probe	This study
Actin_R	2050	gtaccctcatcaggcatctg	Southern probe	This study
SB_probe_LINE-1_F	2344	tctctggacgagcctgttccaa	Southern probe	[2]
SB_probe_LINE-1_R	2345	ggctaagtcgtcagttgaatgc	Southern probe	[2]
miRNA168	32	gtcgccgagaagatcctccatc	sRNA northern	This study
U6_probes	13 and 14	ggccatgctaatcttctctgtatcgtt and ccaattttatcggatgtccccgaagggac	sRNA northern	This study
amiR_*h4.v-kd*_probe	3180	taggcacgatcggattgtcta	sRNA northern	This study
miRNA160	2225	tggcatacagggagccaggca	sRNA northern	This study
miRNA156	3094	gtgctcactctcttctgtcaa	sRNA northern	This study
MITE siRNA	3430	ggtcccacctgtcatacacacact	sRNA northern	[3]
miRNA169	2199	caggcaagtcatccttggcta	sRNA northern	This study
rRNA S7A and S7B (Region marked as 1)	3478 and 3479	tgttttggtcagggtcacgacaatgatcct and gcggtctgttttggtcagggtcacg	rRNA Northern	[4]
rRNA p42 (Region marked as 3)	3480	Gcctcgcgcgcgagcgctcggcgggcaggggtga	rRNA Northern	[4]
rRNA p22 (Region marked as 4)	3477	Ctgtcgtcttccgagagcatct	rRNA Northern	[4]

1. Abad, M.A., Ruppert, J.G., Buzuk, L., Wear, M., Zou, J., Webb, K.M., Kelly, D.A., Voigt, P., Rappsilber, J., Earnshaw, W.C., et al. (2019). Borealin-nucleosome interaction secures chromosome association of the chromosomal passenger complex. J. Cell Biol. *218*, 3912–3925.

2. Cui, X., Jin, P., Cui, X., Gu, L., Lu, Z., Xue, Y., Wei, L., Qi, J., Song, X., Luo, M., et al. (2013). Control of transposon activity by a histone H3K4 demethylase in rice. Proc. Natl. Acad. Sci. U. S. A. *110*, 1953–1958.

3. Hari Sundar G, V., Swetha, C., Basu, D., Pachamuthu, K., Raju, S., Chakraborty, T., Mosher, R.A., and Shivaprasad, P.V. (2023). Plant polymerase IV sensitizes chromatin through histone modifications to preclude spread of silencing into protein-coding domains. Genome Res. *33*, 715–728.

4. Hang, R., Wang, Z., Deng, X., Liu, C., Yan, B., Yang, C., Song, X., Mo, B., and Cao, X. (2018). Ribosomal RNA biogenesis and its response to chilling stress in Oryza sativa. Plant Physiol. *177*, 381–397.

Supplementary Table 5. Details of *in vitro* reconstitution of NCPs using rice histones

Sl. No.	Histone or DNA source	Sequence	E. coli expression conditions and/or remarks
1	Widom 601 NPS 146 bp	5′-<br>CTGGAGAATCCCGGTGCCGAGGCCGCTCAATTGGTCGTAG<br>ACAGCTCTAGCACCGCTTAAACGCACGTACGCGCTGTCCC<br>CCGCGTTTTAACCGCCAAGGGGATTACTCCCTAGTCTCCA<br>GGCACGTGTCACATATATACATCCTG-3′	N/A
2	Widom 601 NPS 188 bp	5′-<br>GGACCCTATCGCGAGCCAGGCCTGAGAATCCCGGTGCCGA<br>GGCCGCTCAATTGGTCGTAGACAGCTCTAGCACCGCTTAA<br>ACGCACGTACGCGCTGTCCCCCGCGTTTTAACCGCCAAGG<br>GGATTACTCCCTAGTCTCCAGGCACGTGTCACATATATAC<br>ATCCAGGCCTTGTGTCGCGAAATTCATA-3′	N/A
3	H2A (Plasmid RFF4, pVHS85)	**DNA Sequence: (Cloned into pET28a, NcoI and NotI)**<br>**ATGGCAGGTCGTGGTAAAGCAATTGGTAGCGGTGCAGCAA**<br>**AAAAAGCAATGAGCCGTAGCAGCAAAGCAGGTCTGCAGTT**<br>**TCCGGTTGGTCGTATTGCACGTTTTCTGAAAGCAGGTAAA**<br>**TATGCAGAACGTGTTGGTGCGGGTGCACCGGTTTATCTGG**<br>**CAGCAGTTCTGGAATACCTGGCAGCCGAAGTGCTGGAACT**<br>**GGCAGGTAATGCAGCACGTGATAACAAAAAAACCCGTATT**<br>**GTTCCGCGTCATATTCAGCTGGCAGTTCGTAATGATGAAG**<br>**AACTGAGCCGTCTGCTGGGCACCGTTACCATTGCAAGCGG**<br>**TGGTGTTATGCCGAATATTCATAATCTGCTGCTGCCGAAA**<br>**AAAGCCGGTGGTAGCGCAAAAGCAGCAGCCGGTGATGATG**<br>**ATAATTAA**<br>**Protein Sequence:**<br>MAGRGKAIGSGAAKKAMSRSSKAGLQFPVGRIARFLKAGK<br>YAERVGAGAPVYLAAVLEYLAAEVLELAGNAARDNKKTRI<br>VPRHIQLAVRNDEELSRLLGTVTIASGGVMPNIHNLLLPK<br>KAGGSAKAAAGDDDN	Expressed in *E. coli* Rosetta Gami pLysS. Induced with 0.2 mM IPTG at 0.5 OD and grown for 3 h at 37 ^{o}C.
4	H2B (Plasmid code RFR, pVHS107)	**DNA Sequence: (Cloned into pET28a, NcoI and NotI)**<br>ATGGCGCCTAAAGCAGAAAAAAAACCGGCAGCCAAAAAAC<br>CTGCAGAAGAAGAACCTGCCGCAGAAAAAGCCGAAAAAGC<br>TCCGGCAGGTAAAAAACCGAAAGCGGAAAAACGTCTGCCT<br>GCCGGTAAAGCTGAAAAAGGTAGCGGTGAAGGCAAAAAAG<br>CAGGTCGTAAAAAAGCTAAAAAAAGCGTGGAAACCTACAA<br>AATCTATATCTTTAAGGTGCTGAAACAGGTGCATCCGGAT<br>ATTGGTATTAGCAGCAAAGCAATGAGCATCATGAACAGCT<br>TTATCAACGACATCTTTGAAAAACTGGCAGGCGAAAGCGC<br>CAAACTGGCACGTTATAACAAAAAACCAACCATTACCAGC<br>CGTGAAATTCAGACCAGCGTTCGTCTGGTTCTGCCTGGTG<br>AACTGGCAAAACATGCAGTTAGCGAAGGCACCAAAGCAGT<br>TACCAAATTTACCAGCGCATAA<br>**Protein Sequence:**<br>**MAPKAEKKPAAKKPAEEEPAAEKAEKAPAGKKPKAEKRLP**<br>**AGKAEKGSGEGKKAGRKKAKKSVETYKIYIFKVLKQVHPD**<br>**IGISSKAMSIMNSFINDIFEKLAGESAKLARYNKKPTITS**<br>**REIQTSVRLVLPGELAKHAVSEGTKAVTKFTSA**	Expressed in *E. coli* Rosetta Gami pLysS. Induced with 0.2 mM IPTG at 0.5 OD and grown for 3 h at 37 ^{o}C.

5	H3<br>(Plasmid code REY2, pVHS80)	**DNA Sequence: (Cloned into pET21a, NdeI, AvrII)**<br>5’-<br>ATGGCCCGCACCAAGCAGACGGCGAGGAAGTCCACCGGCG<br>GCAAGGCGCCGAGGAAGCAGCTGGCGACGAAGGCGGCGCG<br>CAAGTCGGCCCCGGCCACCGGCGGCGTGAAGAAGCCCCAC<br>CGCTTCCGCCCCGGCACCGTCGCGCTCCGGGAGATCCGCA<br>AGTACCAGAAGAGCACCGAGCTGCTGATCCGCAAGCTGCC<br>GTTCCAGCGCCTGGTGCGGGAGATCGCGCAGGACTTCAAG<br>ACCGACCTCCGCTTCCAGAGCTCCGCCGTCGCCGCGCTGC<br>AGGAGGCGGCCGAGGCCTACCTCGTCGGGCTCTTCGAGGA<br>CACCAACCTCTGCGCCATCCACGCCAAGCGCGTCACCATC<br>ATGCCCAAGGACATCCAGCTCGCCCGCCGCATCCGTGGCG<br>AGAGGGCCTAG-3’<br>**Protein Sequence:**<br>MARTKQTARKSTGGKAPRKQLATKAARKSAPATGGVKKPH<br>RFRPGTVALREIRKYQKSTELLIRKLPFQRLVREIAQDFK<br>TDLRFQSSAVAALQEAAEAYLVGLFEDTNLCAIHAKRVTI<br>MPKDIQLARRIRGERA	Expressed in *E. coli* Rosetta Gami pLysS. Induced with 0.2 mM IPTG at 0.5 OD and grown for 3 h at 37 ^{o}C.
6	H4+H3 Co-expressed in the same plasmid (Plasmid:RGR, pVHS106)	**H3 DNA Sequence: (Cloned into pRSFDuet1, NdeI and AvrII)**<br>5’-<br>ATGGCCCGCACCAAGCAGACGGCGAGGAAGTCCACCGGCG<br>GCAAGGCGCCGAGGAAGCAGCTGGCGACGAAGGCGGCGCG<br>CAAGTCGGCCCCGGCCACCGGCGGCGTGAAGAAGCCCCAC<br>CGCTTCCGCCCCGGCACCGTCGCGCTCCGGGAGATCCGCA<br>AGTACCAGAAGAGCACCGAGCTGCTGATCCGCAAGCTGCC<br>GTTCCAGCGCCTGGTGCGGGAGATCGCGCAGGACTTCAAG<br>ACCGACCTCCGCTTCCAGAGCTCCGCCGTCGCCGCGCTGC<br>AGGAGGCGGCCGAGGCCTACCTCGTCGGGCTCTTCGAGGA<br>CACCAACCTCTGCGCCATCCACGCCAAGCGCGTCACCATC<br>ATGCCCAAGGACATCCAGCTCGCCCGCCGCATCCGTGGCG<br>AGAGGGCCTAG-3’<br>**H3 Protein Sequence:**<br>MARTKQTARKSTGGKAPRKQLATKAARKSAPATGGVKKPH<br>RFRPGTVALREIRKYQKSTELLIRKLPFQRLVREIAQDFK<br>TDLRFQSSAVAALQEAAEAYLVGLFEDTNLCAIHAKRVTI<br>MPKDIQLARRIRGERA<br><br>**H4 DNA Sequence: (Cloned into pRSFDuet1, NcoI and HindIII sites)**<br>ATGggatccATGAGCGGTCGTGGTAAAGGTGGCAAAGGTT<br>TAGGTAAAGGCGGTGCAAAACGTCATCGTAAAGTTCTGCG<br>TGATAATATTCAGGGTATTACCAAACCGGCAATTCGTCGT<br>CTGGCACGTCGTGGTGGTGTTAAACGTATTAGCGGTCTGA<br>TTTATGAAGAAACCCGTGGTGTTCTGAAAATCTTTCTGGA<br>AAATGTTATTCGTGACGCCGTTACCTATACCGAACATGCA<br>CGTCGTAAAACCGTTACCGCAATGGATGTTGTTTATGCAC<br>TGAAACGTCAGGGTCGTACCCTGTATGGTTTTGGTGGTTA<br>A<br>**H4 Protein Sequence:**<br>MGSMSGRGKGGKGLGKGGAKRHRKVLRDNIQGITKPAIRR<br>LARRGGVKRISGLIYEETRGVLKIFLENVIRDAVTYTEHA	Expressed in *E. coli* Rosetta Gami pLysS. Induced with 0.2 mM IPTG at 0.5 OD and grown for 3 h at 37 ^{o}C.

		RRKTVTAMDVVYALKRQGRTLYGFGG	
7	H4.V+H3 Coexpressed in the same plasmid (RGP, pVHS104)	**H3 DNA Sequence: (Cloned into pRSFDuet1, NdeI and AvrII sites)**<br>5'-<br>ATGGCCCGCACCAAGCAGACGGCGAGGAAGTCCACCGGCG<br>GCAAGGCGCCGAGGAAGCAGCTGGCGACGAAGGCGGCGCG<br>CAAGTCGGCCCCGGCCACCGGCGGCGTGAAGAAGCCCCAC<br>CGCTTCCGCCCCGGCACCGTCGCGCTCCGGGAGATCCGCA<br>AGTACCAGAAGAGCACCGAGCTGCTGATCCGCAAGCTGCC<br>GTTCCAGCGCCTGGTGCGGGAGATCGCGCAGGACTTCAAG<br>ACCGACCTCCGCTTCCAGAGCTCCGCCGTCGCCGCGCTGC<br>AGGAGGCGGCCGAGGCCTACCTCGTCGGGCTCTTCGAGGA<br>CACCAACCTCTGCGCCATCCACGCCAAGCGCGTCACCATC<br>ATGCCCAAGGACATCCAGCTCGCCCGCCGCATCCGTGGCG<br>AGAGGGCCTAG-3'<br>**H3 Protein Sequence:**<br>MARTKQTARKSTGGKAPRKQLATKAARKSAPATGGVKKPH<br>RFRPGTVALREIRKYQKSTELLIRKLPFQRLVREIAQDFK<br>TDLRFQSSAVAALQEAAEAYLVGLFEDTNLCAIHAKRVTI<br>MPKDIQLARRIRGERA<br><br>**H4.V DNA Sequence: (Cloned into pRSFDuet1, NcoI and NotI sites)**<br>ATGGCACCGCGTAGCGTTGCAATTAGCGGTCGTGGCACCA<br>GCGGTGCACGTCGTCATCGTATTGTTTTTCGTGGTTATAT<br>TCAGGGTATTGCCAAACCGGTTATTCGTCGTCTGGCACGT<br>AAAGGTGGTGTTAAACGTATTAGTGGCCTGATCTACAAAG<br>AGACCCGCGGTGTGCTGGAAATTTTCCTGAAAAATGTGAT<br>TCGCGACGCAATTACCTACACCGAGCACGCCCATCGCAAG<br>ACCGTCATGGCGATGGATGTGGTGTACGCGCTCAAGCTGC<br>AGGGGCGCACCATTTACGATTTCGGAGGCTAA<br>**H4.V Protein Sequence:**<br>MAPRSVAISGRGTSGARRHRIVFRGYIQGIAKPVIRRLAR<br>KGGVKRISGLIYKETRGVLEIFLKNVIRDAITYTEHAHRK<br>TVMAMDVVYALKLQGRTIYDFGG	Expressed in *E. coli* Rosetta Gami pLysS. Induced with 0.2 mM IPTG at 0.5 OD and grown for 3 h at 37 ^{o}C.
8	H4.V_S (Plasmid code RFG, pVHS90)	**H4.V_S DNA Sequence: (Cloned into pET28a, NcoI and NotI sites, Gibson assembly)**<br>5'-<br>ATGGCACCGCGTAGCGTTGCAATTAGCGGTCGTGGCACCA<br>GCGGTGCACGTCGTCATCGTATTGTTTTTCGTGGTTATAT<br>TCAGGGTATTGCCAAACCGGTTATTCGTCGTCTGGCACGT<br>AAAGGTGGTGTTAAACGTATTAGTGGCCTGATCtacgagg<br>agacccgcggtgtgctgaaggtgttcctggagaatgtgat<br>tcgggacgcagtcacctacaccgagcacgccaagcgcaag<br>accgtcacagccatggatgtggtgtacgcgctcaagcgcc<br>aggggcgcaccctgtacggcttcggaggctag-3'<br>**H4.V_S Protein Sequence:**<br>MAPRSVAISGRGTSGARRHRIVFRGYIQGIAKPVIRRLAR<br>KGGVKRISGLIYEETRGVLKVFLENVIRDAVTYTEHAKRK<br>TVTAMDVVYALKRQGRTLYGFGG	Expressed in *E. coli* Rosetta Gami pLysS. Induced with 0.2 mM IPTG at 0.5 OD and grown for 3 h at 37 ^{o}C.
9	H4.V_S V33A (Plasmid code RHN,	**H4.V_S V34A DNA Sequence: (Cloned into pET28a, NcoI and NotI sites, Gibson assembly)**	Expressed in *E. coli* Rosetta Gami Induced with 0.25

	pVHS122)	5'-<br>ATGGCACCGCGTAGCGTTGCAATTAGCGGTCGTGGCACCA<br>GCGGTGCACGTCGTCATCGTATTGTTTTTCGTGGTTATAT<br>TCAGGGTATTGCCAAACCGGcgATTCGTCGTCTGGCACGT<br>AAAGGTGGTGTTAAACGTATTAGTGGCCTGATCtacgagg<br>agacccgcggtgAgctgaaggtgttcctggagaatgtgat<br>tcgggacgcagtcacctacaccgagcacgccaagcgcaag<br>accgtcacagccatggatgtggtgtacgcgctcaagcgcc<br>aggggcgcaccctgtacggcttcggaggctag-3'<br><br>**H4.V_S V34A Protein Sequence:**<br>MAPRSVAISGRGTSGARRHRIVFRGYIQGIAKPAIRRLAR<br>KGGVKRISGLIYEETRGELKVFLENVIRDAVTYTEHAKRK<br>TVTAMDVVYALKRQGRTLYGFGG	mM IPTG at 0.5 OD and grown for 2 h at 37 °C.
10	6xHis-H4can (Plasmid PE1170)	**6xHis-H4 DNA sequence: (Cloned into pET vector)**<br>5'-<br>ATGGGCAGCAGCCATCATCATCATCATCACAGCAGCGGCC<br>TGGTGCCGCGCGGCAGCCATATGTCAGGAAGAGGAAAAGG<br>AGGAAAAGGGTTAGGCAAAGGAGGAGCAAAGAGACACAGA<br>AAGGTTCTAAGAGACAACATTCAAGGAATCACAAAGCCAG<br>CGATTCGTCGTCTTGCTCGTAGAGGAGGTGTGAAGAGAAT<br>CAGTGGATTGATCTATGAAGAAACGAGAGGTGTGTTGAAG<br>ATTTTTCTGGAGAATGTGATTAGAGATGCTGTTACTTACA<br>CTGAGCATGCGAGGAGGAAGACGGTGACTGCTATGGATGT<br>TGTTTATGCCTTGAAGAGACAAGGAAGAACTCTATATGGA<br>TTTGGTGGTTAA-3'<br>**6xHis-H4 protein sequence:**<br>MGSSHHHHHHSSGLVPRGSHMSGRGKGGKGLGKGGAKRHR<br>KVLRDNIQGITKPAIRRLARRGGVKRISGLIYEETRGVLK<br>IFLENVIRDAVTYTEHARRKTVTAMDVVYALKRQGRTLYG<br>FGG	Expressed in *E. coli* Rosetta Gami Induced with 0.25 mM IPTG at 0.5 OD and grown for 2 h at 37 °C.

Supplementary Table 6: Details of cryo-EM data collection and processing.

	H4 NCP	H4.Vs NCP
Number of grids used	1	1
Grid type	Quantifoil R2/1 200 mesh + 2 nm carbon	Quantifoil R2/1 200 mesh
Microscope/detector	Titan Krios / Falcon4i	Titan Krios / Falcon4i
Voltage	300 kV	300 kV
Magnification	165k	165k
Recording mode	Counting mode	Counting mode
Dose rate	1 e^-/ Å^2/frame	1 e^-/ Å^2/frame
Defocus	-0.5 µm to -2.6 µm (step size 0.3 µm)	-0.5 µm to -2.6 µm (step size 0.3 µm)
Pixel size	0.727	0.727
Total dose	60 e^-/Å^2	60 e^-/Å^2
Number of frames/movie	60	60
Total exposure time	Adjusted to keep total dose stable	Adjusted to keep total dose stable
Number of micrographs	10,395	31,174
Number of micrographs used	10,139	23,019
Number of particles used	140,877	148,217
PDB	8Q15	8Q16
EMDB	EMD-18060	EMD-18061
Map resolution (FSC 0.143)	3.6	3.6
**Refinement (Phenix)**		
Resolution (Å)	4.0	3.6
Map CC	0.79	0.84
Mean B factor (Å^2)	17.81 (protein) 88.23 (DNA)	43.02 (protein) 129.02 (DNA)
**Validation**		
All atom clashscore	3.75	3.69
Rotamer outliers (%)	1.27	0.50
MolProbity score	1.27	1.32
**Ramachandran plot**		
Favored (%)	97.86	97.07
Outliers (%)	0.00	0.00
**RMS deviation**		
Bond length (Å)	0.003	0.004
Bond angle (°)	0.520	0.544
