# Supplementary material for: Oryza genera-specific novel Histone H4 variant predisposes H4 Lysine5 Acetylation marks to modulate salt stress responses
Source: Nat Plants. Author manuscript; Available in PMC 2025 May 14. (PMC7617672; doi:10.1038/s41477-025-01974-2)
Supplement: Supplementary Information [file EMS204895-supplement-Supplementary_Information.pdf]

For all the analyses and phenotyping at least 30 plants were used. Seeds obtained from genotype-confirmed T2 plants were sterilised using ethanol, bleach and 0.1% $HgCl_2$ and germinated on ½ MS media with 0.3% Phytagel (Sigma Aldrich) and for salt stress 120 mM NaCl was supplemented. Seedlings were germinated in dark for 5 days and then grown in light for further 14 days before phenotyping.

NCPs stability assay

NCP stability assay using SYPRO orange dye was performed as described earlier [105]. Fluorescence was measured on a BioRad CFX96 real time system in a FRET mode following manufacturer's protocol and the dF/dT computation was normalised to base fluorescence at 26 ^{o}C.

Immunoblotting

Total nuclear protein from 0.4 g rice seedlings were extracted as described earlier [106]. The proteins were electrophoresed on a 14% SDS-PAGE gel and blotted onto Protran supported nitrocellulose membrane as described [108]. The membrane was hybridised with α-H4K5Ac (Merck 07-327, 1:2500), α-H3 (Merck 07-10254, 1:20000), α-H4 (Abcam ab10158, 1:2500) or α-H4.V (Custom generated, 1:1000) in a 5% milk containing 1x TBST buffer. For the bacterial proteins, 25 µl culture of 2.0 OD induced cells were lysed in equal volume 2x Laemmli buffer.

Multi-stress transcriptome dataset analyses

Gene expression datasets were downloaded from TENOR [50] website (https://tenor.dna.affrc.go.jp/). Fold changes in seedling-shoot gene expression was calculated for all kinds of stresses by normalising to the corresponding control dataset. The fold change matrix was subset for the DEGs of interest, merged with the similarly processed *h4.v KO* and salt stress datasets and the non-zero entries were taken for principal component analyses using prcomp package in R. The data visualisation was done using the tools of factoextra package.

Supplementary Information

Supplementary Table 1. Histone H4 sequences from different species.

Supplementary Table 2. Details of high-throughput genomics data generated in this study.

Supplementary Table 3. Details of high-throughput genomics data obtained from publicly available datasets.

Supplementary Table 4. List of oligos and probes used in this study.

Supplementary Table 5. Details of *in vitro* reconstitution of NCPs using rice histones.

Supplementary Table 6. Details of cryo-EM data collection and processing.

Supplementary Dataset 1. Lists of ChIP peaks identified.

Supplementary Dataset 2. List of DEGs identified upon salt stress and in genotypes.

Declaration of competing interests

The authors declare that they have no known competing financial interests or personal relationships that could have appeared to influence the work reported in this paper.

Data availability

All raw and processed sequencing data generated in this study have been submitted to the NCBI Gene Expression Omnibus (GEO; https://www.ncbi.nlm.nih.gov/geo/) under accession number GSE229604. Coordinates and the cryo-EM maps for the rice H4 NCP and H4.V_S NCP have been deposited in the Protein Data Bank (PDB) and Electron Microscopy Data Bank (EMDB). Accession codes are: H4 NCP (PDB:8Q15, EMDB: EMD-18060) and H4.V_S NCP (PDB:8Q16, EMDB: EMD-18061).

To review GEO accession GSE229604:

Go to https://www.ncbi.nlm.nih.gov/geo/query/acc.cgi?acc=GSE229604

Enter token **mdujicuypvsbfij** into the box

Acknowledgements

We thank Prof. K. Veluthambi for *Agrobacterium* strains, rice seeds, and binary plasmids. We thank NGGF, CIFF, IT, radiation, cryo-EM, greenhouse and laboratory-kitchen facilities at the NCBS. We would like to thank Dr Katja Lammens
